# Synthesis of a Series of Trimeric Branched Glycoconjugates and Their Applications for Supramolecular Gels and Catalysis

**DOI:** 10.3390/molecules28166056

**Published:** 2023-08-14

**Authors:** Jonathan Bietsch, Anji Chen, Dan Wang, Guijun Wang

**Affiliations:** Department of Chemistry and Biochemistry, Old Dominion University, Norfolk, VA 23529, USA; jbietsch@odu.edu (J.B.); achen@odu.edu (A.C.); d2wang@odu.edu (D.W.)

**Keywords:** glycoconjugates, glycoclusters, catalysis, click chemistry, triazoles, green synthesis, gelators, organogelators, hydrogelators, metallogels

## Abstract

Carbohydrate-derived molecular gelators have found many practical applications as soft materials. To better understand the structure and molecular gelation relationship and further explore the applications of sugar-based gelators, we designed and synthesized eight trimeric branched sugar triazole derivatives and studied their self-assembling properties. These included glucose, glucosamine, galactose, and maltose derivatives. Interestingly, the gelation properties of these compounds exhibited correlations with the peripheral sugar structures. The maltose derivative did not form gels in the tested solvents, but all other compounds exhibited gelation properties in at least one of the solvents. Glucose derivatives showed superior performance, followed by glucosamine derivatives. They typically formed gels in toluene and alcohols; some formed gels in ethanol-water mixtures or DMSO water mixtures. The glycoclusters **9** and **10** demonstrated rate acceleration for the copper-catalyzed azide-alkyne cycloaddition (CuAAC) reactions. These were further studied for their metallogels formation properties, and the copper metallogels from compound **9** were successfully utilized to catalyze click reactions. These metallogels were able to form a gel column, which was effective in converting the reactants into the triazole products in multiple cycles. Moreover, the same gel column was used to transform a second click reaction using different reactants. The synthesis and characterization of these compounds and their applications for catalytic reactions were discussed.

## 1. Introduction

The molecular self-assemblies formed by carbohydrate derivatives have exhibited many useful properties and applications. Using carbohydrate-based building blocks, supramolecular functional materials with desired properties can be designed and synthesized. Carbohydrate-based self-assembling systems, especially those that form supramolecular gels, are especially important due to their broad applications. For example, sugar-based low molecular weight gelators (LMWGs) have demonstrated utility in many different research areas including environmental remediation and in the biomedical fields [1,2,3]. Different classes of LMWGs have been discovered and demonstrated in applications in biomedicines and drug delivery [4,5], and in environmental remediation for pollutants removal [6,7]. Another promising area for the applications of molecular gelators is for organic synthesis and catalysis [8,9,10,11,12]. Gels formed from LMWGs have been reported to catalyze several aldol addition types of reactions [13,14,15]. In addition to organic small-molecule-based gelators, metallogels formed by small molecules and metal ions have been extensively studied. These metallogels exhibit properties that are useful in smart materials, metal recognition, sensing, and catalysis [16,17,18,19,20]. Several metallogels containing polymeric or oligomeric catalytic groups have been prepared and these gels are used as catalysts [21,22].

The copper-catalyzed azide-alkyne cycloaddition (CuAAC) reaction, “click” chemistry, has played an important role in the construction of various carbohydrate-based self-assembling systems and glycoconjugates [23,24,25]. Using click chemistry, many triazole-containing glycoconjugates have been prepared and exhibited molecular gelation properties [26,27,28]. In addition, due to the importance of click chemistry, many methods and experimental tools have been developed to catalyze the reactions. The use of triazole-based ligands has especially exhibited great promise in the rate acceleration of reactions [29,30]. Several groups have reported triazole-based gelators as ligands that can accelerate CuAAC reactions [31,32,33].

Multiple branched glycoclusters or glycodendrimers have shown importance due to the formation of well-organized assemblies with practical and theoretical applications [34,35]. Branched glycoconjugates have been synthesized and their formation of self-assembling systems has led to applications in medicinal chemistry and bioorganic chemistry [36,37,38]. Glycodenrimersomes have been shown to have unique self-assembling properties and they have also been found to bind to proteins such as lectins and galectins [39]. Branched or dendritic compounds containing appropriate functional groups have been explored for the formation of LMWGs [40]. There are certain advantages of using the dendritic design for building molecular self-assembling systems due to the multiple sites of interactions and the ability to use identical proximal functional groups that have predictable interactions with the solvents or other moieties. For instance, we have shown that increasing the number of branches in triazole-linked glycoclusters can lead to the formation of effective molecular gelators [26]. Several non-gelator sugar derivatives were tethered to a central pentaerythritol core and the gelation properties versus the number of branches were analyzed. We found that, typically, three or four units of sugar triazoles produced molecules with enhanced gelation properties. In the formation of effective gelators, the optimal repeating branches seem to be three or four, and hexamers were typically insoluble in most solvents [33]. The clusters with fewer branches were found to be effective supramolecular gelators and the hexameric glycoclusters were found to efficiently accelerate copper-sulfate-mediated cycloaddition reactions. The creation of glycoconjugates with well-defined structures and sizes in between small molecules and polymers is a pathway for discovering new materials with desirable physical and mechanical properties. In this research, we aim to design and synthesize new sugar derivatives and analyze their gelation properties. Due to the fact that the trimeric branched molecules that we previously explored exhibited good gelation properties, different sugar azides are used to form trimeric glycoclusters in a one-step reaction. The products are evaluated for their gelation and catalysis properties for click reactions.

## 2. Results and Discussions

**Figure 1:**
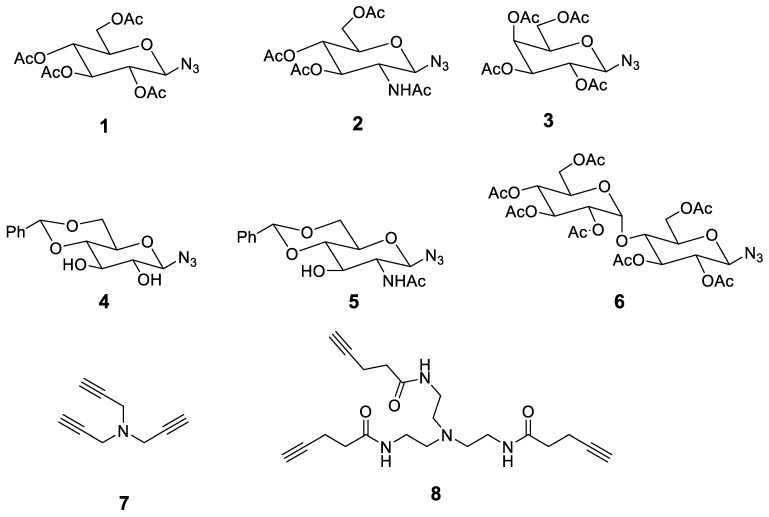
Structures of the building blocks **1**–**8** for the preparation of glycoconjugates.

**Figure 2:**
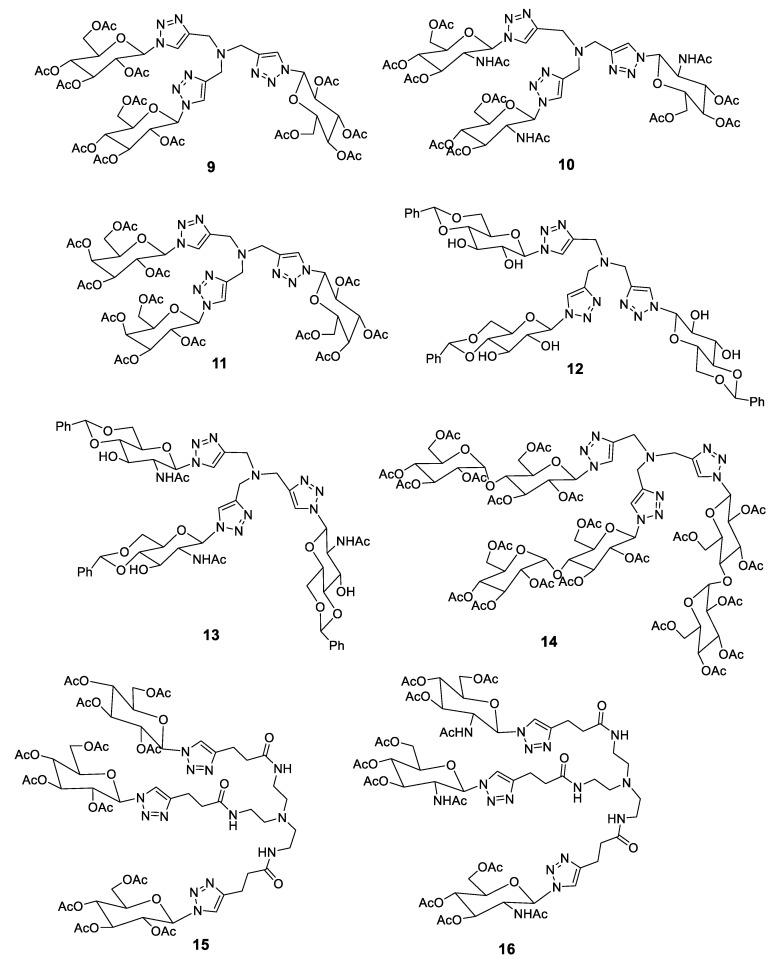
Structures of glycoclusters **9**–**16** with three arms of sugar triazoles.

Using click chemistry, we synthesized a series of trimeric branched glycoconjugates. Figure 1 shows the structures of sugar azides **1**–**6** and alkyne building blocks **7**–**8**. The sugar azides were prepared by procedures in the literature, these included the per-acetylated glucose, glucosamine, and galactose anomeric azides **1**–**3** [41], 4, 6-*O*-protected glucose and glucosamine anomeric azides **4**, **5** [42], as well as the per-acetylated maltose azide **6** [43]. Using these azides and their reactions with two trimeric branched alkynes **7**–**8** through click reaction, we synthesized eight glycoclusters **9**–**16**, as shown in Figure 2. After these compounds were obtained, their gelation properties were screened in several solvents and the results are shown in Table 1.

**Figure 3:**
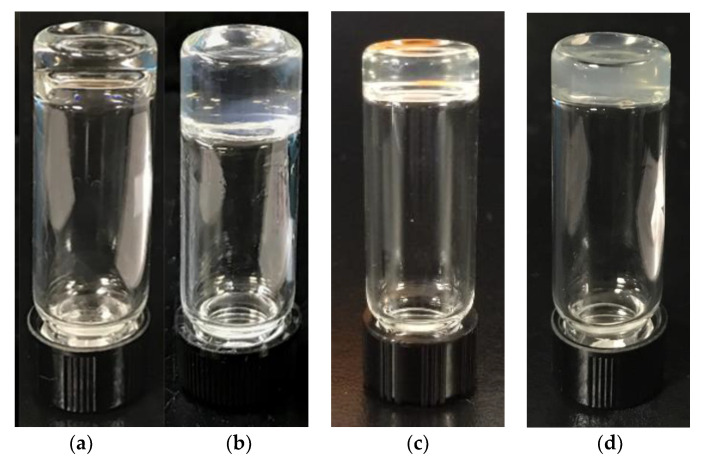
(**a**) A clear gel formed in toluene by compound **9** at 4.0 mg/mL; (**b**) a clear/translucent gel formed by compound **10** in *n*-PrOH at 3.3 mg/mL, (**c**) compound **15** in *n*-butanol at 5.0 mg/mL, (**d**) compound **15** in *i*-PrOH at 4.0 mg/mL.

The gelation test results indicate that effective LMWGs were obtained through the rational design of multimeric glycoconjugates. Among the solvents tested, all monosaccharide derivatives formed gels in at least one of the solvents. The gelation and self-assembling properties of these derivatives depend on the structures of the sugar azide. The galactose derivative **11** only formed a gel in glycerol at a 20 mg/mL concentration, and the disaccharide sugar derivative **14** did not form gels in any of the tested solvents. The best-performing compound and most versatile gelator in the series was the glucose derivative compound **9**. Compound **9** formed gels in ten different solvents, including gels in toluene and alcohols at relatively low concentrations. In contrast, when the glucose was replaced with glucosamine as in compound **10**, a less effective gelator was obtained. Compound **10** formed gels in *n*-propanol and *n*-butanol, but not in toluene and ethanol. The benzylidene acetal derivatives **12** and **13** formed gels in a few solvent systems. The glucosamine derivative **13** was a more efficient gelator between the two compounds, forming gels in six solvents at relatively lower concentrations. Using the tri-alkyne building block compound **8**, glycoclusters **15** and **16** were prepared. In these glycoclusters, the triazole ring was further away from the central nitrogen and the compound has an additional amide functional group. Extending the distance between the peripheral sugar group and the central nitrogen provides the structure with additional flexibility. These two compounds similarly performed as gelators for alcohols; however, the glucose derivative **15** was a better-performing gelator, forming gels at lower concentrations than the glucosamine derivative **16.** Compound **15** also formed organogels in toluene. The organogels formed by these clusters in alcohols appeared mostly transparent or translucent; a few examples of the gels are shown in Figure 3.

**Figure 4:**
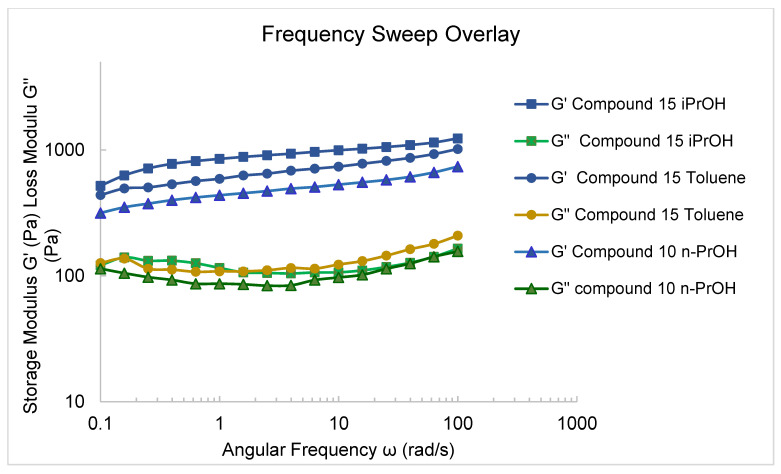
Rheological properties of the organogels formed by compounds **10** and **15**. Compound **10** in *n*-PrOH at 3.3 mg/mL, compound **15** in toluene at 3.3 mg/mL, and compound **15** in *i*-PrOH at 4.0 mg/mL. All experiments were carried out at 1% strain.

The rheological properties of a few organogels have been characterized by rheology. The rheological properties of the organogels formed by compounds **10** and **15** are shown in Figure 4 and Figures S1–S3. The gels all have greater storage moduli G′s than their loss moduli G″s (Tables S1–S3), which indicates that the gels are mechanically stable as semisolid/liquid. All three gels have relatively low G′ values, these indicate that the gels are not stiff, the strength of the gels can be increased by making the concentration higher than the MGCs.

**Figure 5:**
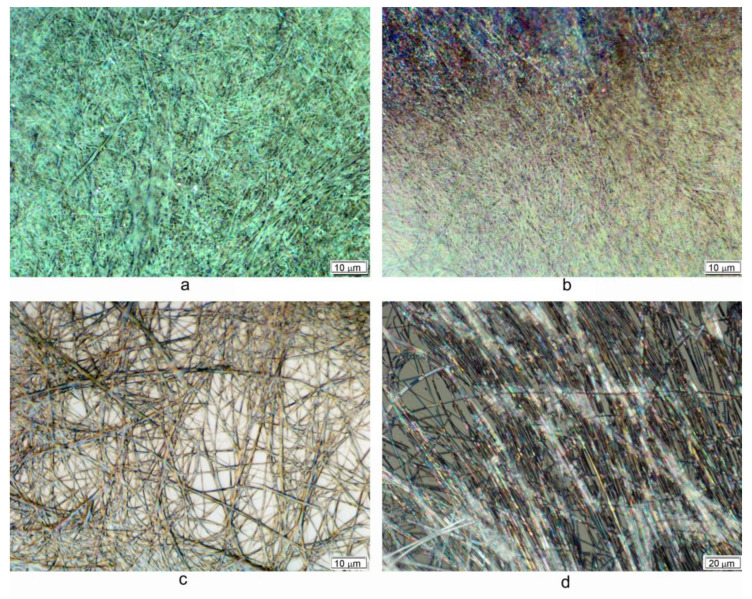
Optical micrographs of the gels: (**a**) compound **9** in toluene at 4.0 mg/mL; (**b**) compound **9** in EtOH at 4.0 mg/mL; (**c**) compound **9** in *i*-PrOH at 3.3 mg/mL; (**d**) compound **10** in EtOH:H_2_O (*v*/*v*, 1:2) at 2.8 mg/mL.

The morphology of the gels was characterized using optical microscopy; a few selected images are included in Figure 5. Compound **9** formed stable gels in toluene, ethanol, and isopropanol. The toluene gel showed a densely packed, curved, and long fibrous network (Figure 5a). The morphology of the gelator assemblies in ethanol appeared as uniform intertwined fibers with diameters of about 0.3 μm (Figure 5b). In isopropanol, the gel formed a similar morphology, but with some fibers bundled together appearing as a range of thin to thick fibers with diameters from 0.3 μm and many of them clustered together (Figure 5c). The gel formed by compound **10** in EtOH:H_2_O (*v*/*v*, 1:2) exhibited long uniform and birefringent tubules or cylindrical fibers, these fibers were typically over 500 μm in length (Figure 5d).

**Figure 6:**
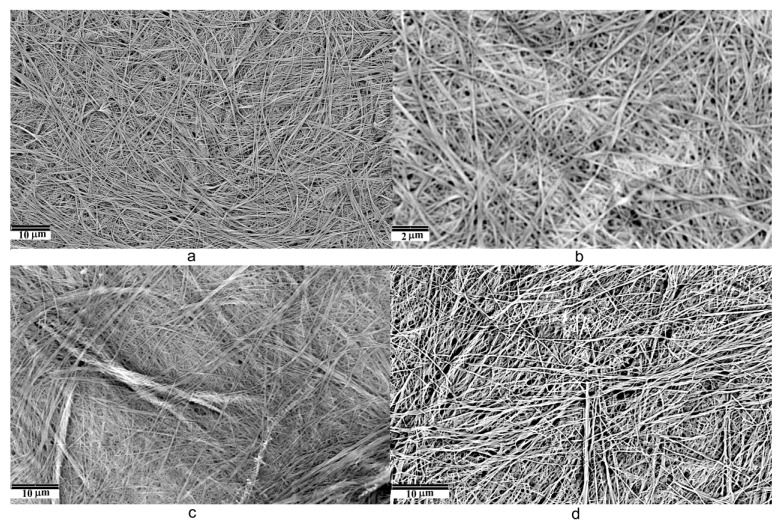
Scanning electron micrographs of the gels formed by compound **9** in different solvents. (**a**) In EtOH at 4.0 mg/mL; (**b**) in *n*-propanol at 3.3 mg/mL; (**c**) in EtOH:H_2_O (*v*/*v* 1:2) at 6.7 mg/mL; (**d**) compound **9** and CuSO_4_ (1:1 molar ratio) in EtOH:H_2_O (*v*/*v* 1:2) at 6.7 mg/mL.

The scanning electron micrographs (SEM) of compound **9** are shown in Figure 6. Typically, these gels formed long and uniform fibrous networks. Compared to the OM images, the SEMs revealed more detailed fibrous features that were more resolved. The gel in EtOH at 4.0 mg/mL exhibited a uniform long fibrous network (Figure 6a). In *n*-propanol, the gel exhibited similar long and narrow intertwined fibers with estimated diameters of less than 0.2 μm (Figure 6b). The gel in EtOH:H_2_O (*v*/*v* 1:2) at 6.7 mg/mL exhibited a densely packed long uniform fibrous network (Figure 6c). Certain areas of the fibrous networks were bundled together and appeared as braided ropes. The same gel in the presence of Cu (II) exhibited more refined fibers with smaller diameters in comparison to Figure 6c. The addition of the copper ions seemed to stabilize the fibrous network to a certain extent. The fibers were curved and, in certain areas, the rods were twisted in the formation of helical ribbons (Figure 6d).

### Metallogels Formation and Applications as Catalytic Gels

**Scheme 1:**
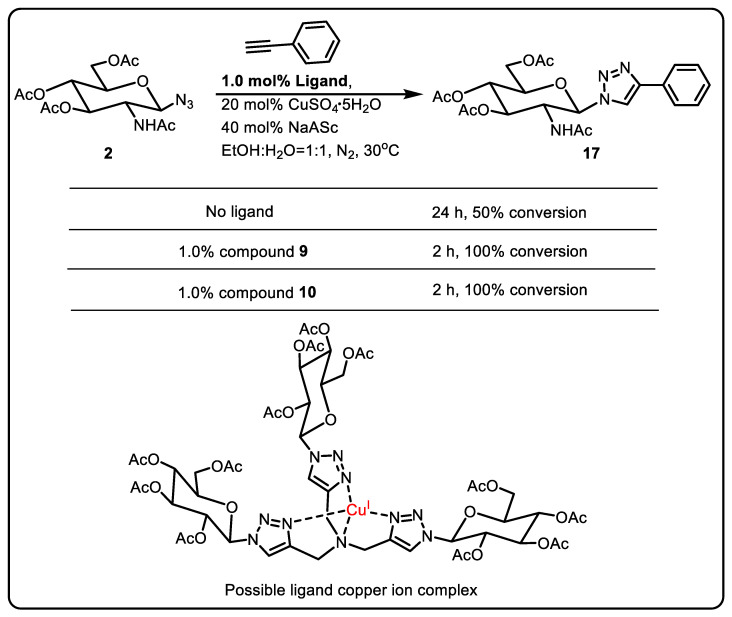
The acceleratory effects of the trimeric ligand in the click reaction and the possible ligand copper ion interaction as the active catalyst.

With the versatile gelator compounds **9** and **10** in hand, we next studied their potential applications for supramolecular catalysis. Previously, we demonstrated that certain trimeric and hexameric glycoclusters were able to form metallogels with CuSO_4_ and the resulting gels were useful in catalyzing click reactions. The trimeric gelators obtained in this study were easy to prepare and the two compounds were tested for their properties in catalyzing click reactions. As shown in Scheme 1, the click reaction of compound **2** with phenyl acetylene slowly proceeded at 30 °C, reaching only 50% conversion at 24 h. However, with the addition of 1.0% compounds **9** or **10**, the reactions proceeded with 100% conversion in less than 2 h. Therefore, these gelators showed significant reaction rate acceleration, which indicates that we can utilize the metallogels for click reactions. We expect that the other tris triazolyl derivatives **11**–**14** also have similar rate accelerating effects for the click reaction. Further studies were focused on compounds **9** and **10**, since they exhibited superior gelation properties compared to the rest. The possible ligand and copper ion interactions are shown in the scheme, based on several reports in the literature on the mechanisms of tris(triazolyl methyl)-amine-type ligands [44,45,46,47].

To further explore the applications of the gelators for catalysis, compounds **9** and **10** were tested for gelation in the presence of CuSO_4_ in EtOH/H_2_O (*v*/*v* 1:2). The GluNAc derivative **10** formed an opaque gel at 5.0 mg/mL, which was prepared using 2.0 mg compound **10** in 0.4 mL of solvent. To this gel, 0.01 mL of CuSO_4_ stock solution (0.24 mg/mL) was added. The copper (II) ion to gelator ratio was 0.6 equiv. A control gel sample was also prepared, and 0.01 mL of water was added to the gel instead. Interestingly, the gel exposed to CuSO_4_ became unstable after 1 h and more liquid-like at 24 h but was still cloudy, and turned into a clear solution at 36 h. The control, however, was stable at 36 h and no visible decomposition of the gel was observed (Figure S5).

The glucose derivative **9** was also tested and it formed stable gels in the presence of CuSO_4_ in EtOH/H_2_O (*v*/*v* 1:2), as shown in Figure S6. This was therefore explored for catalysis for the click reaction. First, we ran test reactions in suspension/solution and then on a metallogel. The reaction shown in Scheme 1 to prepare compound **17** was utilized as a model click reaction. ^1^H NMR spectroscopy was utilized to monitor reaction conversion. The reaction was monitored at 30 min, 1 h, 2 h, and 3 h. The ^1^H NMR spectra of the reaction run as a suspension at different times are shown in Figures S7–S12. At 3 h, ^1^H NMR indicated 95.8% conversion. The reaction was removed from the shaker and the solvent was removed under vacuum to afford the crude product, which was purified by column chromatography using pure DCM to 3% MeOH/DCM to give a white solid (54.2 mg, 85.1%) as the desired product.

For the reaction on the metallogel, full conversion was observed at 2 h and the reaction mixture was removed from the shaker. The reaction details are shown in Figures S7 and S13–S16. The solvent was removed under vacuum to afford the crude product, which was purified by column chromatography using pure DCM to 3% MeOH/DCM to give a white solid (59.6 mg, 93.6%) as the desired product. In comparison to the suspension, the reaction on the gel exhibited faster conversion, with 48% conversion at 30 min, 87% conversion at 1 h, and 100% conversion at 2 h. The reaction carried out in suspension showed 19% conversion at 30 min, 57% conversion at 1 h, and 84% conversion at 2 h. This result indicates that using gel for the click reaction is feasible and more efficient than using compound **9** as a ligand in the solution.

**Scheme 2:**
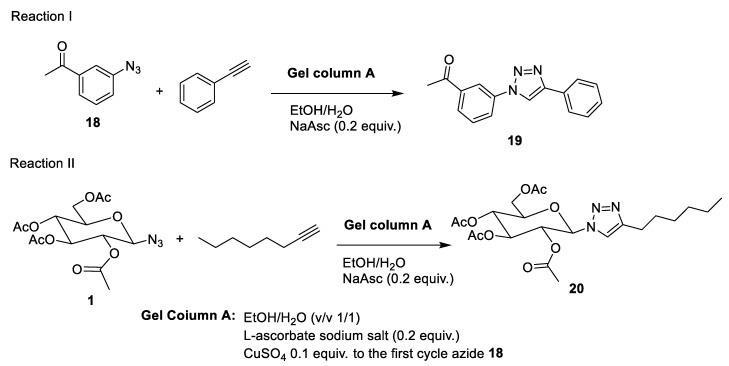
The preparation of compounds **19** and **20** using the metallogel column A formed by compound **9**.

The metallogels of compound **9** exhibited excellent rate acceleration for the click reaction. This gel was then evaluated for whether it could be used as a catalyst for the stationary phase in a gel column and we carried out the reaction by passing reactants through the gel column. We analyzed and tested the feasibility of using the gel to continuously catalyze click reactions. Two click reactions were selected to analyze the applicability of the gel column, as shown in Scheme 2.

**Figure 7:**
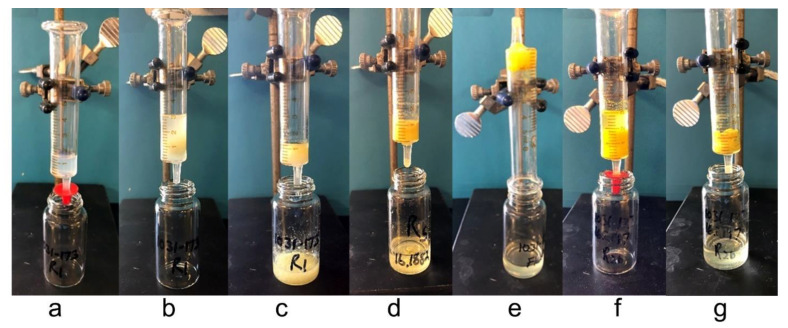
(**a**) A gel formed by compound **9** in EtOH/H_2_O (*v*/*v* 1:2) and CuSO_4_ pentahydrate in a syringe/column (gel column A) with a plug. (**b**) A solution of phenylacetylene, compound **18**, and NaAsc dissolved in 1 mL of EtOH/H_2_O (v 1:2) was added on top of the gel column. (**c**) After the gel column was flushed, the gel inside the column was ready for another round of reaction mixture addition. This process was repeated five times. (**d**) The gel column and eluted product after round 5, (**e**) the gel column after 3 mL ethanol wash, the column was ready for the next reaction. (**f**) In the second cycle of the synthesis of compound **20** using the gel column, reactants **1**, 1-octyne and NaAsc were loaded to the gel column, (**g**) the gel column after two cycles of the experiment.

The metallogel and gel column are shown in Figure 7 and Figure S17. Detailed reaction monitoring and ^1^H NMR spectra are included in Figures S18–S44. A gel was prepared using compound **9** (10 mg, 0.008 mmol) inside a one-dram vial with EtOH/H_2_O (v 1:2) 1.5 mL and CuSO_4_ pentahydrate (6 mg, 0.022 mmol, 0.1 equiv.), as shown in Figure S17a,b. The gel was reheated and transferred to a syringe/column with a stopper (red plug for the syringe tip), and the gel successfully reformed after cooling (Figure 7a). Then, the reactants were added on top of the gel. A solution of phenylacetylene (30 μL, 0.27 mmol, 1.2 equiv.), azido derivative **18** (35 mg, 0.22 mmol, 1 equiv.), and L-ascorbate sodium salt (NaAsc) (9 mg, 0.044 mmol, 0.2 equiv.) dissolved in 1 mL of EtOH/H_2_O (*v*/*v* 1:2) was added on top of the gel column. The reaction mixture was allowed to sit on top of the gel column for 1 h, at which point the cap was removed and elution occurred over a 15-min period of time (Figure 7b). After elution was completed, 1 mL of EtOH/H_2_O (*v*/*v* 2:1) was added to the gel column followed by 1 mL of EtOH/H_2_O (*v*/*v* 1:1) to elute residual product.

After the gel column was flushed, the gel inside the column was ready for another round of reaction mixture addition (Figure 7c). This same process was repeated five times with reagent loading, eluting, and flushing. The gel columns and NMR spectra of product **19** of each cycle are included in Figures S18–S35. After the fifth cycle, 3 mL of EtOH was added on top of the gel column to flush the residual product from the column (Figure 7d). The total loaded amount of azide **18** was 35 mg × 5 = 175 mg, and the total final product **19** obtained was 276.9 mg in 96% overall yield.

The gel column after the ethanol wash is shown in Figure 7e. The same gel column was then re-used for the second reaction of sugar azide **1** (100 mg) and 1-octyne. The reaction was similarly carried out. The gel columns with the reactants are shown in Figure 7f,g. The reaction mixture dissolved in EtOH/H_2_O was added on top of the gel column. This reactant solution (2 mL) contained sugar azide **1** (50 mg, 0.134 mmol, 1.0 equiv.), 1-octyne (12 μL, 0.161 mmol, 1.2 equiv.), and L-ascorbate sodium salt (6 mg, 0.027 mmol, 0.2 equiv.). The reaction mixture was allowed to sit on top of the gel column for 1 h. The cap was then removed, and elution occurred over 15 min. After the elution was complete, 1 mL of EtOH/H_2_O (*v*/*v* 1:1) was added to the top of the gel to flush the product off the gel column, this was followed by another 0.5 mL of EtOH/H_2_O (*v*/*v* 2:1), and an additional 0.5 mL of EtOH/H_2_O (*v*/*v* 1:1) to prepare the column for the next round of reaction. The second cycle of the reaction was carried out in the same manner as the first round. After the second experiment, 5 mL of pure EtOH was used to flush the gel column. The crude product was collected in scintillation vials and the solvent was removed under reduced pressure. The crude product was taken up in 1% MeOH/DCM and pushed through a silica gel plug to create the pure product and no additional purification was needed. The total amount of product **20** obtained was 122.2 mg in 94.3% yield. The NMR spectra of the crude and pure products are included in Figures S36–S44.

## 3. Experimental Section

### 3.1. General Methods

All solvents and chemicals were purchased from commercial sources and used without additional purification. Purifications were carried out via column chromatography using 230–400 mesh silica gel utilizing gradient solvent systems. Compound characterization was carried out using ^1^H NMR and ^13^C NMR on a Bruker 400 MHz NMR spectrometer. The NMR samples were prepared in DMSO-*d*_6_ or CDCl_3_. The solvent peaks were utilized as internal standards for reporting chemical shifts. CDCl_3_/DMSO-*d*_6_ peaks were calibrated at 7.26/2.50 ppm for ^1^H NMR and 77.00/39.50 ppm for ^13^C NMR. 2D NMR, and experiments (HSQC, COSY) were also conducted for a few compounds. A Fisher Jones melting point apparatus was utilized to obtain melting point measurements. An Agilent LC1260 HPLC system coupled with a 6120B single quad mass spectrometer was utilized to obtain molecular mass measurements.

Gelation Test: This was carried out in a similar way as previously reported. Typically, 2.0 mg of the compound was weighed out in a one-dram vial. Then, 0.1 mL of the selected solvent was added to the vial, giving the initial concentration of 20 mg/mL. The sample was heated and/or sonicated to produce a homogenous solution. Once dissolved, the sample was allowed to cool for at least 15 min for gel formation to occur. The vial inversion method was used to determine the status of the sample; if a gel formed after cooling and no solvent flow was observed after the vial was inverted, the sample was recorded as a gel. This process was repeated until a stable gel no longer formed. The last concentration at which a stable gel was observed was recorded as the minimum gelation concentration (MGC).

Optical microscopy: An Olympus BX60M optical microscope was used to study the morphology of the gels. The microscope was equipped with a DP73-1-51 high performance Peltier cooled 17MP digital camera and the software used was CellSens 1.11. Samples were prepared by transferring small amounts of gel to a clean microscope glass slide that was allowed to air dry before analysis.

### 3.2. Scanning Electronic Microscopy

SEM imaging was carried out on Thermo Scientific Phenom XL G2 Desktop SEM instrument (Waltham, MA, USA). Small aliquots of gel were transferred onto an SEM pin mount specimen holder via glass capillary tubes or spatulas. The specimen holders were then placed in a closed container for 5–7 days. After the samples were sufficiently dried, they were sputtered with silver and imaged.

### 3.3. Synthesis and Characterization of Compounds ***8***–***16***

Synthesis of tris-alkyne **8** [48] 4-pentynoic acid (125 mg, 1.28 mmol, 1 equiv.) was dissolved in DCM (anhydrous, 3 mL) in a 50 mL RBF and reaction cooled to 0 °C. Oxalyl chloride (0.15 mL, 1.78mmol, 1.4 equiv.) was added to the reaction mixture over a period of 10 min. A drop of DMF (anhydrous) was added to the reaction mixture. The reaction was continuously stirred for 2 h from 0 °C to room temperature. ^1^H NMR analysis indicated that the reaction was a full conversion. Tris(2-amino ethyl)amine (0.06 mL, 0.41 mmol, 0.33 equiv.), pyridine (0.20 mL, 2.55 mmol, 2 equiv.), and K_2_CO_3_ (352 mg, 2.55 mmol, 2 equiv.) were added to another flask with 3 mL of DCM. The above-prepared acyl chloride mixture was added to the reaction mixture at 0 °C over a period of 10 min. The reaction was stirred at room temperature for 12 h. After completion, the reaction was quenched with 5% NaHCO_3_ (1 mL) and extracted with DCM (10 mL × 2). The crude product was dried over Na_2_SO_4_ and purified by column chromatography using eluent from pure DCM to 4% MeOH/DCM to afford a white solid (120 mg, 75%) as the desired product (R_f_ = 0.27 in 5% MeOH/DCM). m.p. 147.2–149.7 °C; ^1^H NMR (400 MHz, CDCl_3_) *δ* 6.70 (m, 3H), 3.36–3.30 (m, 6H), 2.61–2.47 (m, 18H), 2.00 (t, *J* = 2.6 Hz, 3H); ^13^C NMR (100 MHz, CDCl_3_) *δ* 171.7, 83.1, 69.3, 54.6, 37.9, 35.2, 14.9. LCMS m/z calculated for C_21_H_31_N_4_O_3_ [M + H]^+^ 387, found 387.

Synthesis of trimeric glycocluster **9**: Tripropargylamine **7** (0.02 mL, 0.139 mmol, 1 equiv.) was added to a dried and nitrogen-flushed 50 mL round-bottomed flask. Then, 6 mL of THF/H_2_O/*t*-BuOH (*v*/*v*/*v*, 1/1/1) was added to the flask, along with CuSO_4_∙5H_2_O (20.8 mg, 0.083 mmol, 0.6 equiv.) and azide **1** (170 mg, 0.457 mmol, 3.3 equiv.). Sodium ascorbate (NaAsc 32.8 mg, 0.167 mmol, 1.2 equiv.) was then added to start the reaction. The reaction mixture was stirred at room temperature for 24 h under a nitrogen atmosphere. The solvent was then removed under reduced pressure. The crude product was purified via column chromatography using 0–5% MeOH/DCM as the eluent to a white solid (130.0 mg, 75%) as the desired product. R_f_ = 0.41 in 3% MeOH/DCM. m.p. 137.0–139.0 °C; ^1^H NMR (400 MHz, CDCl_3_) *δ* 7.95 (s, 3H), 5.87 (d, *J* = 9.3 Hz, 3H), 5.54 (t, *J* = 9.4 Hz, 3H), 5.42 (t, *J* = 9.4 Hz, 3H), 5.24 (t, *J* = 9.4 Hz, 3H), 4.28 (dd, *J* = 12.8, 5.2 Hz, 3H), 4.16 (dd, *J* = 12.8, 2.0 Hz, 3H), 4.03–3.97 (m, 3H), 3.82–3.70 (m, 6H), 2.09–2.02 (m, 27H), 1.86 (s, 9H); ^13^C NMR (100 MHz, CDCl_3_) *δ* 170.5, 169.9, 169.3, 169.0, 145.2, 122.0, 85.8, 75.1, 72.7, 70.4, 67.8, 61.7, 47.2, 20.7, 20.51, 20.47, 20.1; LC-MS (ESI+) [M + H]^+^ m/z calculated for C_51_H_67_N_10_O_27_ 1251.4, found 1251.4.

Synthesis of trimeric glycocluster **10**: A stock solution (23 μL/mL in DCM) of tripropargylamine **7** (0.5 mL, 11.5 μL, 0.08 mol, 1 equiv.) was added to a dried and nitrogen-flushed 50 mL round-bottomed flask. Then, 2 mL of EtOH/H_2_O (*v*/*v*, 1/1) was added to the flask, along with CuSO_4_∙5H_2_O (13 mg, 0.05 mol, 0.6 equiv.) and azide **2** (100 mg, 0.27 mmol, 3.3 equiv.). Sodium ascorbate (19.8 mg, 0.1 mmol, 1.2 equiv.) was then added to start the reaction. The reaction was stirred at room temperature for 24 h under a nitrogen atmosphere. The solvent was removed from the reaction mixture under reduced pressure and CHCl_3_ (10 mL) was added and stirred, creating a yellow slurry. The slurry was then filtered, leaving a yellowing-green solid. A basic EDTA solution (10 mL, 0.01 M EDTA in 5% NaHCO_3_ solution) was added to the solid, creating a slurry. Upon this addition, the solution turned dark green. The slurry was stirred for 10 min and filtered by vacuum filtration. This process was repeated once with DI water (10 mL) to afford a light-yellow solid (83.3 mg, 84%) as the desired product. R_f_ = 0.51 in 7.5% MeOH/DCM. m.p. 295.8–298.4 °C; ^1^H NMR (400 MHz, *d*_6_-DMSO) *δ* 8.26 (s, 3H), 8.10 (d, *J* = 9.1 Hz, 2H), 6.09 (d, *J* = 9.7 Hz, 3H), 5.36 (t, *J* = 9.8 Hz, 3H), 5.11 (t, J = 9.7 Hz, 3H), 4.71 (q, *J* = 9.6 Hz, 3H), 4.32–4.01 (m, 9H), 3.63 (d, *J* = 14.3 Hz, 3H), 3.53 (d, *J* = 14.3 Hz, 3H), 2.02 (s, 9H), 2.00 (s, 9H), 1.97 (s, 9H), 1.58 (s, 9H); ^13^C NMR (100 MHz, *d*_6_-DMSO) *δ* 169.9, 169.6, 169.3, 169.2, 143.5, 122.8, 84.9, 73.4, 72.3, 68.1, 61.7, 52.0, 46.3, 22.2, 20.41, 20.35, 20.2; LC-MS (ESI+) [M + H]^+^ m/z calculated for C_51_H_70_N_13_O_24_ 1248.5, found 1248.5.

Synthesis of trimeric glycocluster **11**: A stock solution (23 μL/mL in THF) of tripropargylamine **7** (0.5 mL, 11.5 µL, 0.08 mol, 1 equiv.) was added to a dried and nitrogen-flushed 50 mL round-bottomed flask. Then, 2 mL of THF/H_2_O/*t*-BuOH (*v*/*v*/*v*, 1/1/1) was added to the flask, along with CuSO_4_∙5H_2_O (13 mg, 0.05 mol, 0.6 equiv.) and azide **3** (100 mg, 0.27 mmol, 3.3 equiv.). Sodium ascorbate (19.8 mg, 0.1 mmol, 1.2 equiv.) was then added to start the reaction. The reaction was stirred at room temperature for 16 h under a nitrogen atmosphere. The solvent was removed from the reaction mixture under reduced pressure. The crude reaction mixture was dissolved in DCM (10 mL) and washed with a basic EDTA solution (10 mL, 0.01 M EDTA in 5% NaHCO_3_ solution). The aqueous layer was then extracted with DCM (10 mL × 2). The organic layers were then washed with DI water (10 mL × 2). The organic layers were combined, dried with Na_2_SO_4_, and filtered, and the solvent was removed under reduced pressure. The crude product was purified via column chromatography using 0–3% MeOH/DCM as the eluent to afford a white solid (88.7 mg, 87%) as the desired product. R_f_ = 0.34 in 3% MeOH/DCM. m.p. 122.2–124.0 °C; ^1^H NMR (400 MHz, CDCl_3_) *δ* 8.00 (s, 3H), 5.82 (d, *J* = 9.3 Hz, 3H), 5.58 (t, *J* = 9.8 Hz, 3H), 5.54–5.50 (m, 3H), 5.24 (dd, *J* = 10.3, 3.5 Hz, 3H), 4.26–4.08 (m, 9H), 3.83–3.63 (m, 6H), 2.21 (s, 9H), 2.01 (s, 9H), 1.97 (s, 9H), 1.84 (s, 9H); ^13^C NMR (100 MHz, CDCl_3_) *δ* 170.2, 170.0, 169.7, 168.9, 144.6, 122.3, 86.2, 73.9, 70.8, 68.0, 66.8, 61.1, 46.8, 20.6, 20.5, 20.4, 20.1; LC-MS (ESI+) [M + H]^+^ m/z calculated for C_51_H_67_N_10_O_27_ 1251.4, found 1251.4.

Synthesis of trimeric glycocluster **12***:* A stock solution (28 μL/mL in EtOH) of tripropargylamine **7** (0.5 mL, 14 μL, 0.10 mol, 1 equiv.) was added to a dried and nitrogen-flushed 50 mL round-bottomed flask. Then, 2 mL of EtOH/H_2_O (*v*/*v*, 1/1) was added to the flask, along with CuSO_4_∙5H_2_O (15 mg, 0.06 mol, 0.6 equiv.) and the azide **4** (100 mg, 0.34 mmol, 3.3 equiv.). Sodium ascorbate (24 mg, 0.12 mmol, 1.2 equiv.) was then added to start the reaction. The reaction was stirred at room temperature for 40 h under a nitrogen atmosphere. The solvent was removed from the reaction mixture under reduced pressure. The crude product was purified via column chromatography using 5–15% MeOH/DCM as the eluent followed by recrystallization in ethanol-water 1:1 to afford a white solid (81.3 mg, 75%) as the desired product. R_f_ = 0.14 in 20% MeOH/DCM. m.p. 206.5–208.3 °C; ^1^H NMR (400 MHz, *d*_6_-DMSO) *δ* 8.32 (s, 3H), 7.53–7.46 (m, 6H), 7.44–7.37 (m, 9H), 5.79 (d, *J* = 9.2 Hz, 3H), 5.70 (d, *J* = 6.1 Hz, 3H), 5.66 (s, 3H), 5.63 (d, *J* = 5.1 Hz, 3H), 4.28–4.20 (m, 3H), 4.02–3.94 (m, 3H), 3.85–3.67 (m, 15H), 3.64 (t. *J* = 9.0 Hz, 3H); ^13^C NMR (100 MHz, *d*_6_-DMSO) *δ* 143.5, 137.6, 128.9, 128.0, 126.3, 123.2, 100.7, 87.6, 80.1, 73.2, 72.8, 68.5, 67.5, 46.9; LC-MS (ESI+) [M + H]^+^ m/z calculated for C_48_H_55_N_10_O_15_ 1011.4 found 1011.4.

Synthesis of trimeric glycocluster **13**: A stock solution (26 μL/mL in EtOH) of tripropargylamine **7** (0.5 mL, 13 µL, 0.09 mol, 1 equiv.) was added to a dried and nitrogen-flushed 50 mL round-bottomed flask. Then, 2 mL of EtOH/H_2_O (*v*/*v*, 1/1) was added to the flask, along with CuSO_4_∙5H_2_O (14 mg, 0.05 mol, 0.6 equiv.) and azide **5** (100 mg, 0.30 mmol, 3.3 equiv.). Sodium ascorbate (22 mg, 0.11 mmol, 1.2 equiv.) was then added to start the reaction. The reaction was stirred at room temperature for 36 h under a nitrogen atmosphere. The solvent was removed from the reaction mixture under reduced pressure. A basic EDTA solution (5 mL, 0.01 M EDTA in 5% NaHCO_3_ solution) was added to the flask, followed by sonication for 5 min. A yellow suspension was formed and then filtered, revealing a light-yellow solid. The filtered solid was washed with CHCl_3_ (10 mL × 2) to give 85.0 mg (83.3%) as the desired product (R_f_ = 0.14 in 20% MeOH/DCM). m.p. 297.6–299.3 °C; ^1^H NMR (400 MHz, *d*_6_-DMSO) *δ* 8.24 (s, 3H), 8.04 (d, *J* = 9.1 Hz, 3H), 7.53–7.46 (m, 6H), 7.43–7.36 (m, 9H), 5.90 (d, *J* = 9.9 Hz, 3H), 5.69 (s, 3H), 4.42–4.20 (m, 6H), 3.88 (t, *J* = 9.0 Hz, 3H), 3.82-3.69 (m, 9H), 3.65–3.49 (m, 6H), 1.65 (s, 9H); ^13^C NMR (100 MHz, *d*_6_-DMSO) *δ* 169.2, 143.3, 137.5, 128.9, 128.0, 126.3, 122.9, 100.8, 86.3, 80.7, 70.4, 68.8, 67.4, 55.1, 46.3, 22.6; LC-MS (ESI+) [M + H]^+^ m/z calculated for C_54_H_64_N_13_O_15_ 1034.5, found 1034.4.

Synthesis of trimeric glycocluster **14**: Tripropargylamine **7** (20 μL, 0.15 mol, 1 equiv.) was added to a dried and nitrogen-flushed 50 mL round-bottomed flask. Then, 4 mL of THF/H_2_O/*t*-BuOH (*v*/*v*/*v*, 1/1/1) was added to the flask, along with CuSO_4_∙5H_2_O (23 mg, 0.09 mol, 0.6 equiv.) and azide **6** (397 mg, 0.60 mmol, 4.0 equiv.). Sodium ascorbate (36 mg, 0.18 mmol, 1.2 equiv.) was then added to start the reaction. The reaction mixture was stirred at room temperature for 48 h under nitrogen atmosphere. The solvent was then removed under reduced pressure. EtOAc (10 mL) and basic EDTA solution (10 mL, 0.01 M EDTA in 5% NaHCO_3_ solution) were added to the flask and stirred for 10 min. The layers were separated and the aqueous layer was back extracted with EtOAc (10 mL × 2). The organic layers were combined, washed with DI water (10 mL × 2), dried with Na_2_SO_4_, filtered, and the solvent was removed under reduced pressure. The crude product was purified via column chromatography using 0–3% MeOH/DCM as the eluent to afford a white solid (244.0 mg, 82%) as the desired product. R_f_ = 0.19 in 3% MeOH/DCM. m.p. 144.7–147.3 °C; ^1^H NMR (400 MHz, CDCl_3_) *δ* 7.91 (s, 3H), 5.86 (d, *J* = 8.9 Hz, 3H), 5.52–5.42 (m, 6H), 5.41–5.31 (m, 6H), 5.07 (t, *J* = 9.9 Hz, 3H), 4.88 (dd, *J* = 10.5, 3.9 Hz, 3H), 4.52 (d, *J* = 11.4 Hz, 3H), 4.30–4.20 (m, 6H), 4.16 (t, *J* = 9.0 Hz, 3H), 4.10–4.03 (m, 3H), 4.02–3.94 (m, 6H), 3.68 (s, 6H), 2.13 (s, 9H), 2.10 (s, 9H), 2.06 (s, 9H), 2.04–1.98 (m, 27H), 1.83 (s, 9H); ^13^C NMR (100 MHz, CDCl_3_) *δ* 170.5, 170.4, 170.3, 169.9, 169.8, 169.4, 169.1, 144.4, 122.5, 95.9, 85.4, 75.4, 75.1, 72.4, 71.1, 70.0, 69.2, 68.8, 68.0, 62.4, 61.5, 46.7, 20.8, 20.7, 20.6, 20.54, 20.53, 20.2; LC-MS (ESI+) [M + H + Na]^2+^/2 m/z calculated for [C_87_H_115_N_10_O_51_Na]/2 1069.4, found 1069.7.

Synthesis of trimeric glycocluster **15:** Tris-alkyne **8** (55 mg, 0.14 mmol, 1 equiv.) was added to a dried and nitrogen-flushed 50 mL round-bottomed flask. Then, 4 mL of EtOH/H_2_O (*v*/*v*, 1/1) was added to the flask, along with CuSO_4_∙5H_2_O (21 mg, 0.084 mmol, 0.6 equiv.) and azide **1** (190 mg, 0.51 mmol, 3.6 equiv.). Sodium ascorbate (34 mg, 0.168 mmol, 1.2 equiv.) was then added to start the reaction. The reaction mixture was stirred at room temperature for 12 h under a nitrogen atmosphere. The solvent was then removed under reduced pressure. The crude product was purified via column chromatography using 0–5% MeOH/DCM as the eluent to afford an off-white solid (168.0 mg, 80%) as the desired product. R_f_ = 0.6 in 10% MeOH/DCM. m.p. 145.0–147.0 °C; ^1^H NMR (400 MHz, *d*_6_-DMSO) *δ* 8.08 (s, 3H), 7.78 (s, 3H), 6.29 (d, *J* = 9.0 Hz, 3H), 5.61 (t, *J* = 9.4 Hz, 3H), 5.54 (t, *J* = 9.3 Hz, 3H), 5.16 (t, *J* = 9.7 Hz, 3H), 4.39–4.32 (m, 3H), 4.12-4.04 (m, 6H), 3.18–3.04 (m, 6H), 2.85 (t, *J* = 7.3 Hz, 6H), 2.50–2.39 (m, 12H), 2.03 (s, 9H), 2.00 (s, 9H), 1.96 (s, 9H), 1.79 (s. 9H); ^13^C NMR (100 MHz, *d*_6_-DMSO) *δ* 170.9, 170.0, 169.5, 169.3, 168.4, 146.7, 120.9, 83.7, 73.2, 72.2, 70.0, 67.5, 61.7, 53.4, 37.0, 34.6, 21.1, 20.4, 20.3, 20.2, 19.8; LC-MS (ESI+) [M + H]^+^ m/z calculated for C_63_H_88_N_13_O_30_ 1506.6 found 1506.5.

Synthesis of trimeric glycocluster **16***:* Tris-alkyne **8** (55 mg, 0.14 mmol, 1 equiv.) was added to a dried and nitrogen-flushed 50 mL round-bottomed flask. Then, 4 mL of EtOH/H_2_O (*v*/*v*, 1/1) was added to the flask, along with CuSO_4_∙5H_2_O (21 mg, 0.084 mmol, 0.6 equiv.) and azide **2** (187 mg, 0.51 mmol, 3.6 equiv.). Sodium ascorbate (34 mg, 0.168 mmol, 1.2 equiv.) was then added to start the reaction. The reaction mixture was stirred at room temperature for 12 h under a nitrogen atmosphere. The solvent was then removed under reduced pressure. The crude product was purified via column chromatography using 0–5% MeOH/DCM as the eluent to afford an off-white solid (154.0 mg, 73%) as the desired product. R_f_ = 0.2 in 10% MeOH/DCM. m.p. 122.0–124.0 °C; ^1^H NMR (400 MHz, *d*_6_-DMSO) *δ* 8.04 (d, *J* = 9.1 Hz, 3H), 7.98 (s, 3H), 7.80 (t, *J* = 5.5 Hz, 3H), 6.07 (d, *J* = 9.9 Hz, 3H), 5.35 (t, *J* = 9.8 Hz, 3H), 5.08 (t, *J* = 9.7 Hz, 3H), 4.60–4.50 (m, 3H), 4.27–4.12 (m, 6H), 4.10–4.02 (m, 3H), 3.17–3.05 (m, 6H), 2.84 (t, *J* = 7.3 Hz, 6H), 2.50–2.37 (m, 12H), 2.01 (s, 9H), 2.00 (s, 9H), 1.95 (s, 9H), 1.60 (s. 9H); ^13^C NMR (100 MHz, *d*_6_-DMSO) *δ* 171.0, 170.0, 169.5, 169.34, 169.27, 146.2, 120.6, 84.5, 73.3, 72.4, 68.0, 61.7, 53.4, 52.1, 37.0, 34.6, 22.3, 21.2, 20.5, 20.4, 20.2; LC-MS (ESI+) [M + H]^+^ m/z calculated for C_63_H_91_N_16_O_27_ 1503.6, found 1503.5.

### 3.4. Synthesis of Compounds ***19*** and ***20*** by Catalytic Gel Column

Gel column preparation: Compound **9** (10 mg, 0.008 µmol, 0.03 equiv.) was added to a 1-dram vial. Then, 1.5 mL of EtOH/water (*v*/*v* 1:2) containing CuSO_4_ pentahydrate (6 mg, 0.022 mmol, 0.2 equiv.) was added and the vial was gently heated until the solids dissolved. The hot solution was then added to a 3 mL syringe (with the plunger removed and a syringe filter/stopper attached). The syringe was then sonicated for 3 min and, once cooled, a stable gel formed. When inverted, the gel in the syringe was stable.

Compound **19** (Round 1–5): A stock solution of phenylacetylene (150 μL, 1.35 mmol, 1.2 equiv.), 3-acetyl phenylazide (175 mg, 1.1 mmol, 1 equiv.), and L-ascorbate sodium salt (45 mg, 0.22 mmol, 0.2 equiv.) in 10 mL of EtOH/water (*v*/*v* 1:1) was prepared. Then, 2 mL of this solution, which contained phenylacetylene (30 μL, 0.27 mmol, 1.2 equiv.), 3-acetyl phenylazide (35 mg, 0.22 mmol, 1 equiv.), and L-ascorbate sodium salt (9 mg, 0.044 mmol, 0.2 equiv.), was added on top of the gel column. The reaction mixture was allowed to sit on top of the gel column for 1 h. The cap was then removed and elution occurred over 15 min. After the elution was complete, 1 mL of EtOH/water (*v*/*v* 2:1) was then added to the gel column, followed by 1 mL of EtOH/water (*v*/*v* 1:1) to flush the remaining product off the gel column. The crude product was collected in a scintillation vial and the solvent was removed under reduced pressure. Crude mixtures were dissolved in 1% MeOH/DCM and pushed through a SiO_2_ plug. For each sample, about 30 mL of 1% MeOH/DCM was required to fully obtain the sample off the SiO_2_ plug. The ^1^H NMR spectra of the crude product and the purified product by filtering through silica gel after each cycle are included in the Supplementary Materials.

Compound **20** (Round 1–2): To demonstrate the reusability of the catalytic gel column, the same gel column used for the synthesis of compound **19** was continued with the loading of different reactants to prepare compound **20**. A stock solution of sugar azide (100 mg, 0.268 mmol, 1.0 equiv.), 1-octyne (24 µL, 0.322 mmol, 1.2 equiv.), and L-ascorbate sodium salt (12 mg, 0.054 mmol, 0.2 equiv.) in ethanol/DI water 1:1 (4 mL) was prepared. Then, 2 mL of this solution, which contained sugar azide (100 mg, 0.268 mmol, 1.0 equiv.), 1-octyne (24 µL, 0.322 mmol, 1.2 equiv.), and L-ascorbate sodium salt (12 mg, 0.054 mmol, 0.2 equiv.), was added on top of the gel column. The reaction mixture was allowed to sit on top of the gel column for 1 h. The cap was then removed and elution occurred over 15 min. After the elution was complete, 1 mL of EtOH/water (*v*/*v* 2:1) was then added to the gel column, followed by 1 mL of EtOH/water (*v*/*v* 1:1) to flush the remaining product off the gel column. The crude product was collected in a scintillation vial and the solvent was removed under reduced pressure. Crude mixtures were dissolved in 1% MeOH/DCM and pushed through a SiO_2_ plug. For each sample, about 30 mL of 1% MeOH/DCM was required to fully obtain the sample off the SiO_2_ plug.

## 4. Conclusions

A series of eight new, branched glycoclusters containing various protected sugar moieties were designed and synthesized through click chemistry. These included per-acetylated glucose, glucosamine, galactose, and maltose derivatives and benzylidene acetal-protected glucose and glucosamine derivatives. We found that many of these trimeric branched compounds were effective gelators, with the per acetylated glucose and glucosamine derivatives performing the best as LMWGs. These compounds were able to form co-gels with copper sulfate. The metallogels were successfully packed into a syringe column and demonstrated catalytic activities for CuAAC reactions. The metallogels were also reusable for multiple cycles of reactants loading in an ethanol and water mixture. These carbohydrate-based gelators were useful as ligands for copper ions and were able to catalyze the click reaction in their aqueous solutions. We anticipate that this gel-based catalyst can be used for catalyzing water-soluble alkynes and azides that are biologically relevant. Further understanding of the structure and gelation relation of sugar templates will lead to the effective design of new functional soft materials. Future studies will focus on an analysis of the gelator-based ligands for other metal ions to optimize the conditions for other reactions.

## Figures and Tables

**Table 1:** Gelation properties of the glycoconjugates synthesized.

Compound	Tol	EtOH	*i*-PrOH	*n*-PrOH	*n*-BuOH	Glycerol	EtOH: H_2_O (1:1)	EtOH: H_2_O (1:2)	DMSO: H_2_O (1:1)	DMSO: H_2_O (1:2)	H_2_O	EG	TG
**9**	G_C_ 4.0	G_T_ 4.0	G_T_ 3.3	G_T_ 3.3	G_T_ 3.3	G_p_ 20.0	G_T_ 6.7	G_T_ 5.0	P	P	P	G_C_ 1.8	G_C_ 10.0
**10**	I	P	I	G_T_ 3.3	G_T_ 3.3	G_c_ 20.0	G_O_ 20.0	G_O_ 2.8	S	I	I	S	S
**11**	S	S	S	S	P	G_p_ 20.0	S	S	P	P	P	P	S
**12**	I	P	P	P	P	G_O_ 20.0	P	P	P	G_O_ 10.0	P	G_O_ 20.0	G_O_ 20.0
**13**	I	I	I	I	I	I	G_p_ 5.0	G_O_ 5.0	G_O_ 6.7	G_O_ 6.7	I	G_O_ 10.0	G_O_ 20.0
**14**	S	P	P	P	P	S	P	P	P	P	P	P	S
**15**	Gc 3.3	S	G_C_ 4.0	G_C_ 5.0	G_C_ 5.0	G_p_ 20.0	S	P	P	P	I	S	S
**16**	I	S	G_T,P_ 20.0	G_C_ 20.0	G_C_ 20.0	G_p_ 20.0	S	P	S	P	I	S	S

G, gel at room temperature; the numbers are the corresponding minimum gelation concentrations (MGCs) in mg/mL; G_C_ (C), clear or transparent gel; G_T_ (T), translucent gel; G_O_ (O), opaque gel; G_p_, unstable or partial gel; I, insoluble; P, crystallize or precipitate; S, soluble at ~20 mg/mL.

## Data Availability

Not applicable.

## Supplementary Materials

The following supporting information can be downloaded at: https://www.mdpi.com/article/10.3390/molecules28166056/s1, The ESI include data of (1) rheological experiment; (2) the trimer clusters as ligands for catalyzing click reactions; (3) ^1^H and ^13^C NMR spectra of compounds **8**–**16** and 2D NMR spectra of compounds **9**–**12** and **14**–**15**. Figure S1–S3, Rheological properties of amplitude sweep experiment for the gel of compound **10** and **15**; Tables S1–S3, Storage modulus (G′), loss modulus (G″) and G′/G″ values. Figure S4, Overlay of ^1^H NMR spectra of starting material and products using compounds **9** and **10** as ligand. Figure S5, Gels formed by compound **10** and response to copper. Figure S6, Suspension and gel preparation of compound **9**. Figure S7, Sample vials for the synthesis of compound **17** using compound **9** as the ligand. Figures S8–S16, ^1^H NMR spectra of the reaction monitoring and product **17** using either suspension or gels. Figure S17, Gel column preparation using compound **9**. Figures S18–S20, Photos of gel column A after the first cycle followed by ^1^H NMR spectra of product **19** before and after SiO_2_ plug treatment. Figures S21–S35, Photos of gel column A after other cycles followed by the ^1^H NMR spectra of product **19**. Figures S36–S44, Photos of gel column A for the synthesis of compound **20** and the ^1^H NMR spectra. Scheme S1, Synthesis of compound **17** at different conditions; Scheme S2, Synthesis of compound **17** using compound **9** at different conditions; Scheme S3, Synthesis of compound **19** using gel column A made by compound **9**; Scheme S4, Synthesis of compound **20** using the gel column A. Figures S45–S53, ^1^H and ^13^C NMR spectra of compounds **8**–**16**, Figures S54–S59, 2D (HSQC and COSY) NMR spectra of compounds **9**–**12** and **14**–**15**.
