# Supplementary material for: Synthesis of a Series of Trimeric Branched Glycoconjugates and Their Applications for Supramolecular Gels and Catalysis
Source: Molecules. 2023 Aug 14;28(16):6056. doi: 10.3390/molecules28166056 (PMC10459207; doi:10.3390/molecules28166056)
Supplement: Supplementary file 1 [file molecules-28-06056-s001.zip › molecules-2516349-supplementary.pdf]

Supplementary Information

Synthesis of a Series of Trimeric Branched Glycoconjugates and Their Applications for Supramolecular Gels and Catalysis

Jonathan Bietsch, Anji Chen, Dan Wang, Guijun Wang*

Department of Chemistry and Biochemistry, Old Dominion University, Norfolk VA 23529, g1wang@odu.edu

Table of Contents

I. Rheological properties of the gels…………..…………………………………….…....S2

II. The trimer clusters as ligands for catalyzing click reactions.……………………….S9

III. ^{1}H and ^{13}C NMR spectra of compounds **8-16**……………………..………………..S39

IV. 2D NMR spectra of compounds **9-12** and **14-15**…………………….…………..S48

I. Rheological properties of the gels

1. Amplitude sweep experiments

Amplitude sweep experiment was performed by HR-2 Discovery Hybrid Rheometer from TA instrument. All gels at their minimum gelation concentration were prepared in 1-dram vial in different solvent (or solvent mixtures) and they were left undisturbed on bench for 2 hrs. Sample (approximately 1 mL) was placed on the steel plate of the rheometer. The experimental temperature was 25 °C. The sample were subjected to amplitude sweep between 25-mm peltier plate and steel plate with a gap of 100 μm. Angular frequency was set as 10.0 rad/s. Operating and processing software is TRIOS. Results were expressed as the storage modules (G'), loss modules (G'') as function of oscillation strain in a range from 0.1% to 125%.

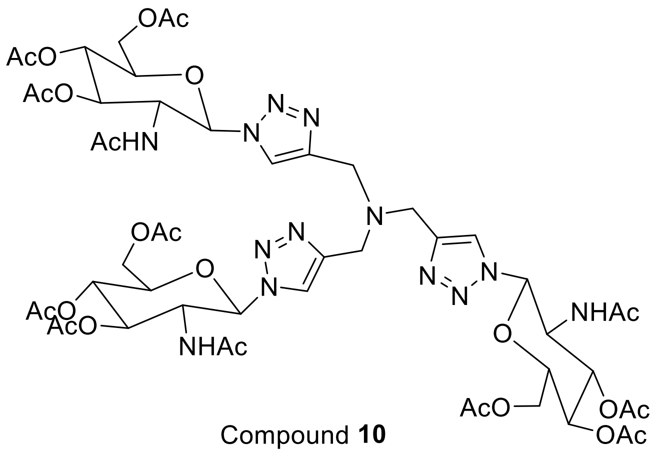

Compound **10**
n-PrOH (3.3 mg/mL)

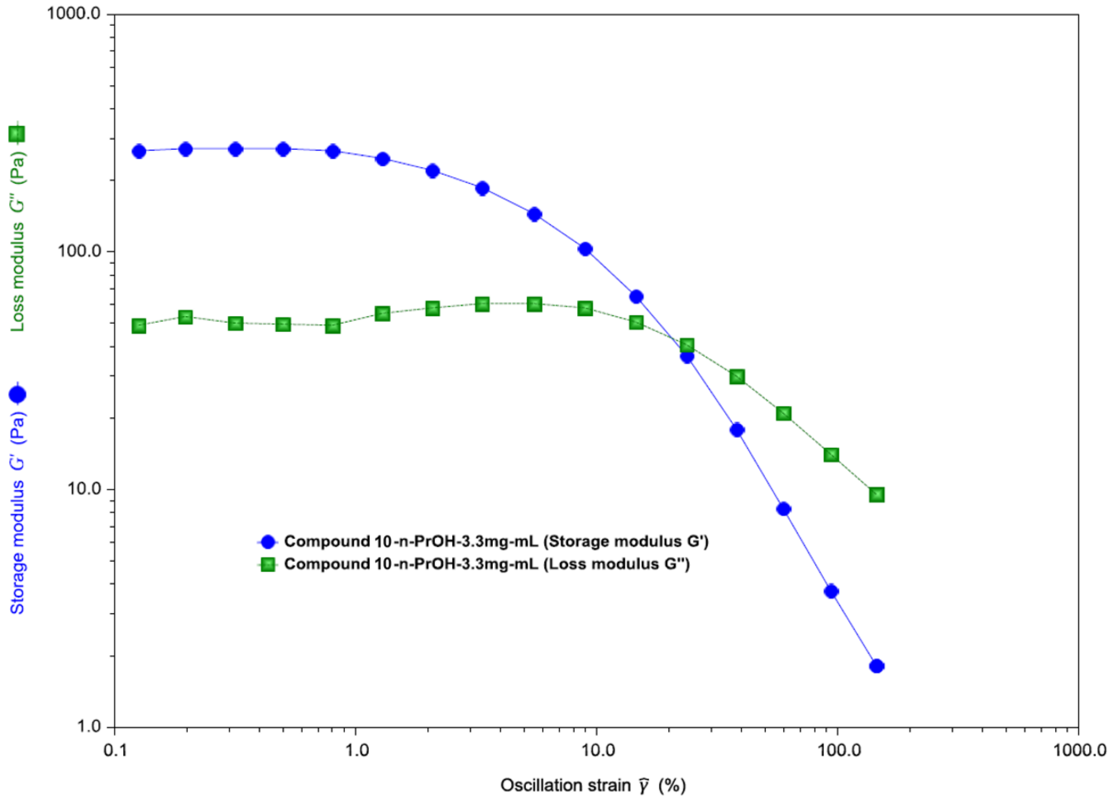

Figure S1. Rheological properties of amplitude sweep experiment for the gel of compound **10** (*n*-PrOH, 3.3 mg/mL).

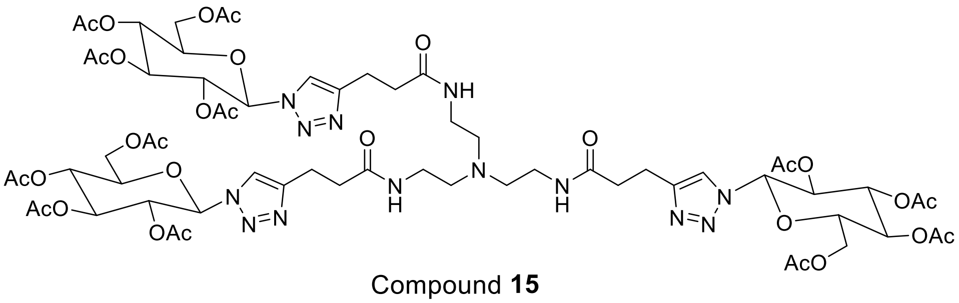

Compound **15**
Toluene (3.3 mg/mL)

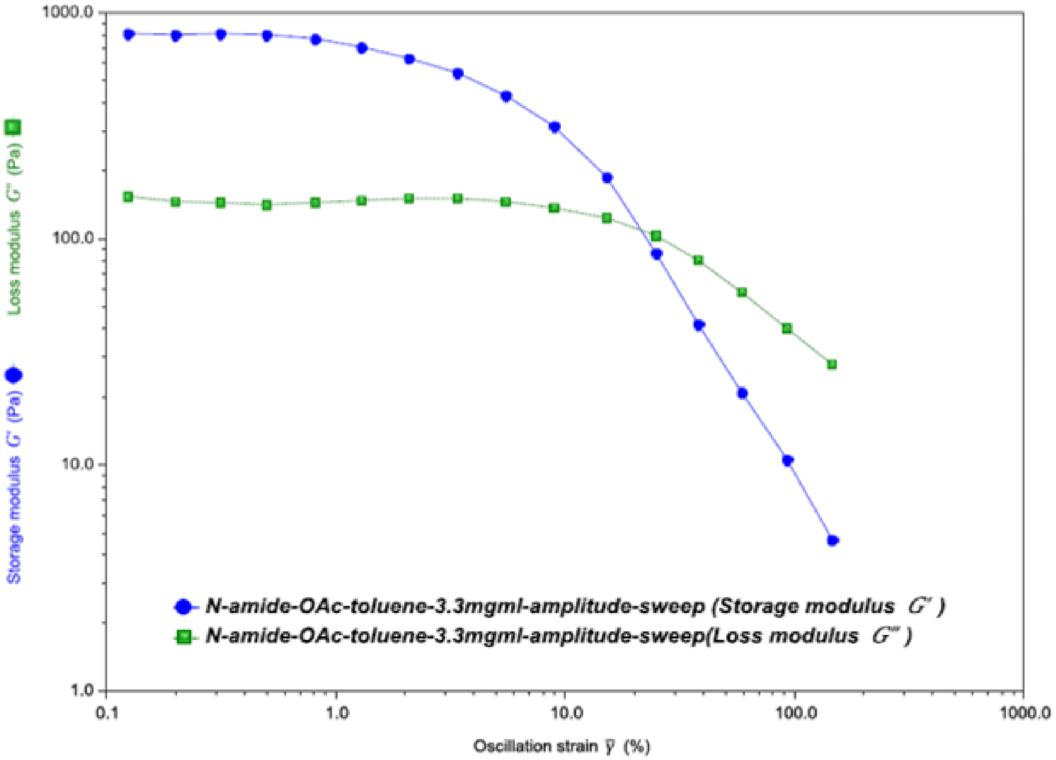

Figure S2. Rheological properties of amplitude sweep experiment for the gel of compound **15** (Toluene, 3.3 mg/mL).

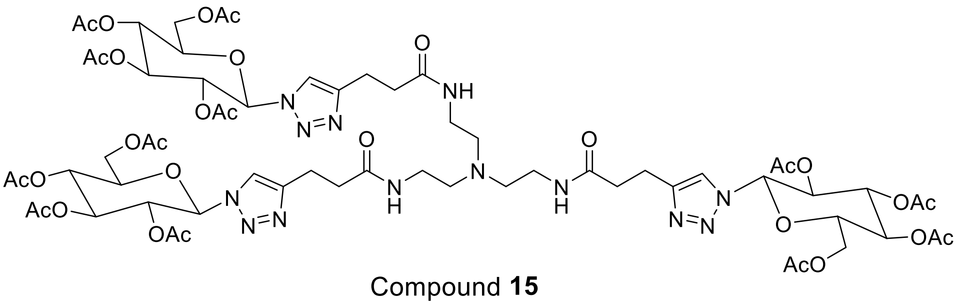

Compound **15**
i-PrOH (4.0 mg/mL)

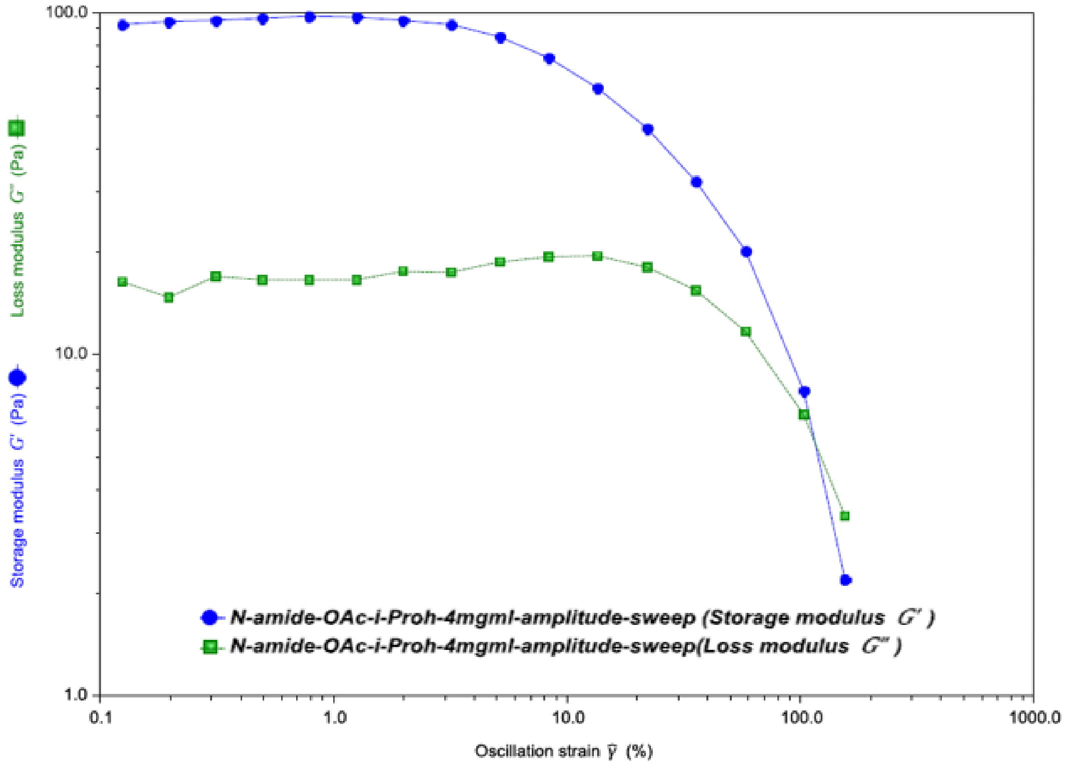

Figure S3. Rheological properties of amplitude sweep experiment for the gel of compound **15** (*i*-PrOH, 4.0 mg/mL).

2. Frequency sweep experimental data

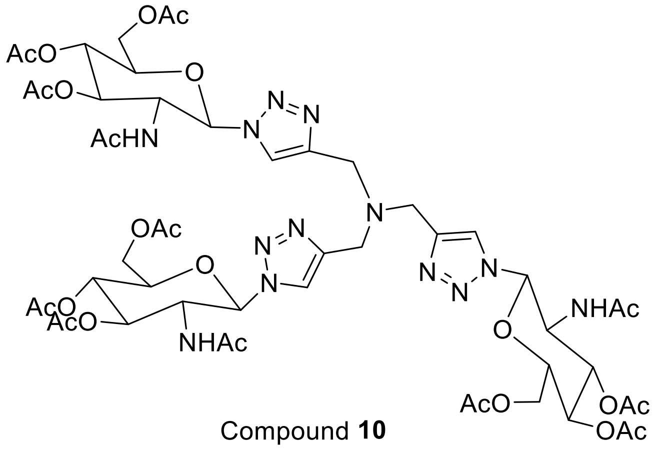

Compound **10**
n-PrOH (3.3 mg/mL)

Compound **10**<br>*n*-PrOH, 3.3 mg/mL			
**Angular frequency**	**Storage modulus (*G′*)**	**Loss modulus (*G″*)**	***G′/G″***
**rad/s**	**Pa**	**Pa**	
0.1	315.938	113.921	2.77
0.15849	350.349	105.028	3.34
0.251189	374.539	97.6609	3.84
0.398107	399.122	92.7615	4.30
0.630957	419.094	86.5942	4.84
1.0	436.698	86.6594	5.04
1.5849	453.448	85.6116	5.30
2.51189	471.293	83.5581	5.64
3.98105	493.152	84.0887	5.86
6.30957	508.222	92.6293	5.49
10.0001	530.761	97.1003	5.47
15.849	553.173	101.983	5.42
25.1188	577.889	113.797	5.08
39.8105	612.04	125.264	4.89
63.0957	663.235	141.954	4.67
100.0	736.974	156.113	4.72

Table S1. Storage modulus (G′), loss modulus (G″) and G′/G″ value for compound **10** (*n*-PrOH, 3.3 mg/mL) under different angular frequency.

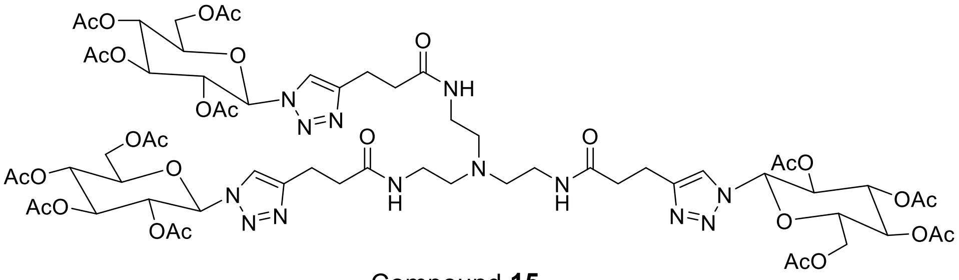

Compound **15**
Toluene (3.3 mg/mL)

Compound **15**<br>Toluene, 3.3 mg/mL			
**Angular frequency**	**Storage modulus (*G′*)**	**Loss modulus (*G″*)**	***G′*/*G″***
**rad/s**	**Pa**	**Pa**	
0.1	437.894	126.376	3.47
0.15849	494.211	136.932	3.61
0.251189	504.026	113.952	4.42
0.398107	534.858	111.966	4.78
0.630957	565.801	107.645	5.26
1.0	589.866	108.663	5.43
1.5849	626.185	108.537	5.77
2.51189	648.449	110.784	5.85
3.98105	683.876	115.596	5.92
6.30957	709.947	114.287	6.21
10.0001	737.619	123.038	6.00
15.849	777.286	131.07	5.93
25.1188	818.111	144.678	5.65
39.8105	864.363	162.64	5.31
63.0957	927.194	179.392	5.17
100.0	1016.75	208.578	4.87

Table S2. Storage modulus (G′), loss modulus (G″) and G′/G″ value for compound **15** (Toluene, 3.3 mg/mL) under different angular frequency.

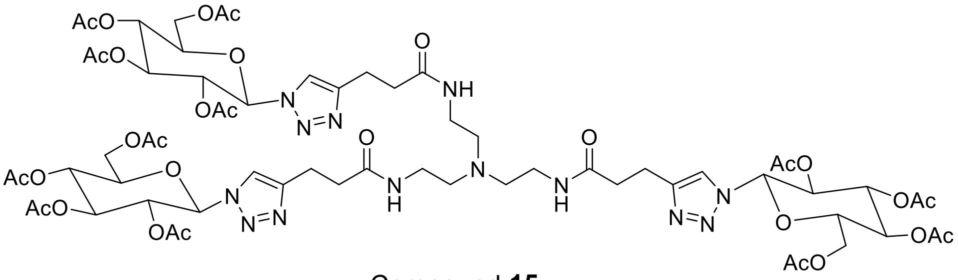

Compound **15**
i-PrOH (4.0 mg/mL)

Compound **15**<br>*i*-PrOH, 4.0 mg/mL			
**Angular frequency**	**Storage modulus (*G′*)**	**Loss modulus (*G″*)**	***G′*/*G″***
**rad/s**	**Pa**	**Pa**	
0.1	519.569	121.267	4.28
0.15849	627.784	140.105	4.48
0.251189	713.203	131.908	5.41
0.398107	775.211	131.98	5.87
0.630957	815.832	125.837	6.48
1.0	849.133	115.568	7.35
1.5849	879.879	106.609	8.25
2.51189	907.842	105.42	8.61
3.98105	933.398	104.475	8.93
6.30957	965.242	106.355	9.08
10.0001	994.931	106.655	9.33
15.849	1022.64	110.514	9.25
25.1188	1054.94	117.585	8.97
39.8105	1094.04	127.152	8.60
63.0957	1145.6	141.758	8.08
100.0	1234.55	164.667	7.50

Table S3. Storage modulus (G′), loss modulus (G″) and G′/G″ value for compound **15** (*i*-PrOH, 4.0 mg/mL) under different angular frequency.

II. The trimer clusters as ligands for catalyzing click reactions

1. Effects of glycoclusters **9** and **10** in CuAAC reaction acceleration study

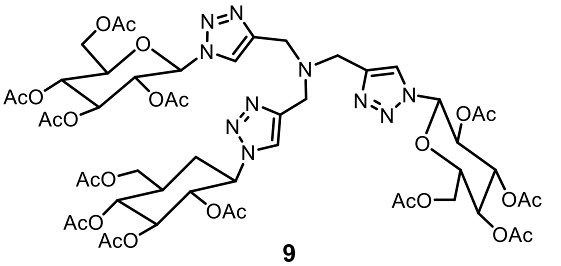

9

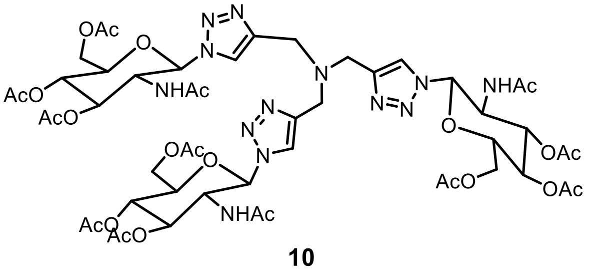

10

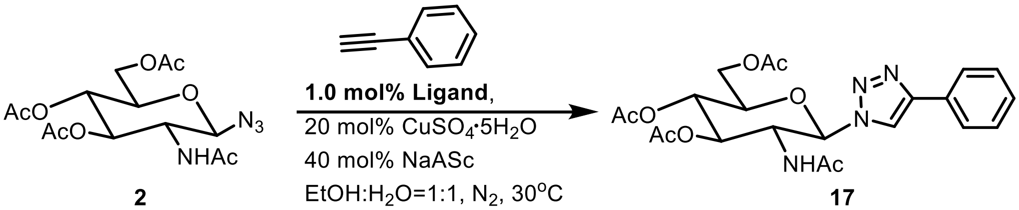

No ligand	24 h, 50% conversion
1.0% compound **9**	2 h, 100% conversion
1.0% compound **10**	2 h, 100% conversion

Scheme S1. Synthesis of compound **17** at different conditions

Sugar azide **2** (40 mg, 0.11 mmol, 1.0 equiv.), $CuSO_4$·$5H_2O$ (5.3mg, 0.02mmol, 0.2 equiv.), NaAsc (8.5 mg, 0.043 mmol, 0.4 equiv.), phenylacetylene (13.1 mg, 0.13 mmol, 1.2 equiv.), ligand (1.0 mol%) and 2 mL of EtOH/H_2O (v/v = 1/1) were added in the given order to the reaction vial. The mixture was then stirred with 500 rpm at 30 °C under N_2. The reaction conversion was determined by ^{1}H NMR. Figure S4 shows the reaction monitoring by ^{1}H NMR spectroscopy for: (a) starting material sugar azide **2**; (b) reaction monitoring at 2 hours using compound **9** (1 mol%) as ligand; and (c) reaction monitoring at 2 hours using compound **10** (1 mol%) as ligand, the arrows point to

the signals used for the calculation of conversion. The percentage conversion was calculated using the integration of anomeric proton of the product (δ 6.04 ppm) divided by the sum of integration of the anomeric proton of the product and the starting material (δ 4.78 ppm).

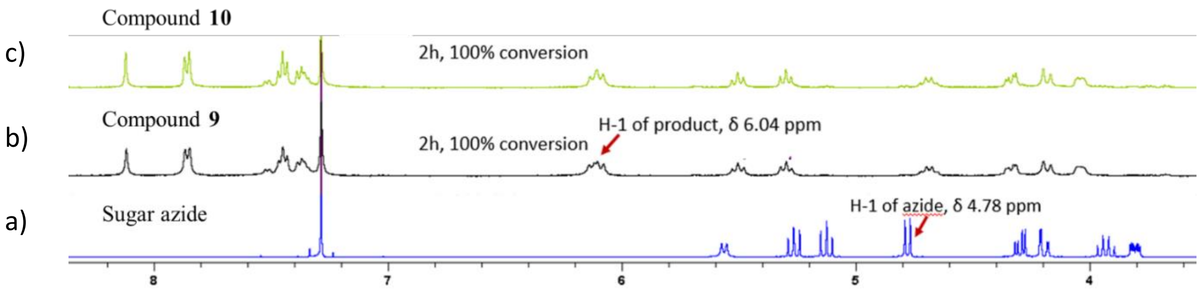

Figure S4. Overlay of ^{1}H NMR spectra of starting material and products using compounds **9** and **10** as ligand.

2. Effect of supramolecular assemblies on catalytic rates

2.1 Metallogel testing for compound **10**

2.0 mg gelator (1.6 µmol) was dissolved in 0.4 mL solvent by gentle heating, after it is cooled to rt, an opaque gel formed. To this gel, 10 µL water was added on top of the gel, this is shown in (a) and (b) labeled as control, the gel was stable after 5 h with small amount of solvent flowing (c), and at 36 h the gels appeared to be mostly stable (d). In contrast, (e-f) shows when 10 µL of $CuSO_4$ solution (0.24 mg/mL, copper (II) is 0.96 µmol) was added; after 1 h the gel became unstable and collapsed (g), the mixture appeared as cloudy suspension as time goes on and at 36 h the mixture turned to clear solution (h).

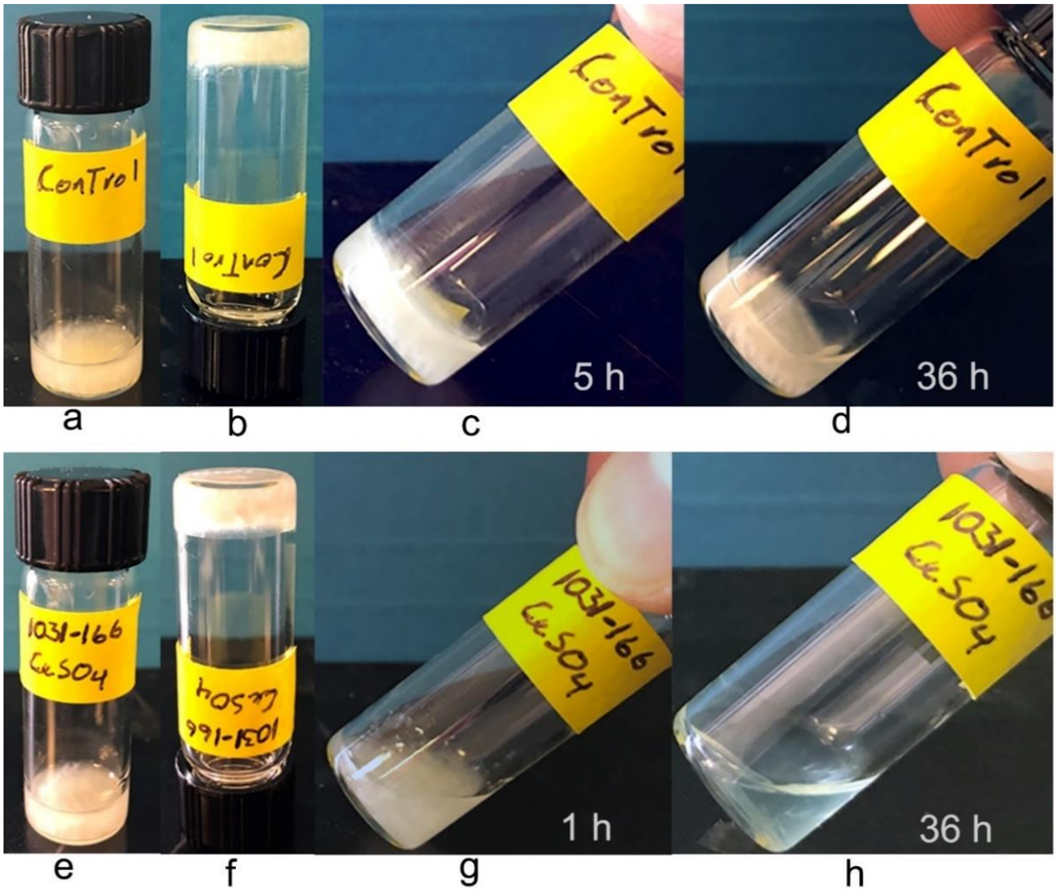

Figure S5. Gels formed by compound **10** and response to copper.

2.2 Suspension and gel preparation of compound **9** in EtOH/H_2O (v/v 1/2)

Suspension preparation: Compound **9** (3.2 mg, 0.0026 mmol, 0.02 equiv.) and $CuSO_4$ pentahydrate (6.5 mg, 0.026 mmol, 0.2 equiv.) was added to a scintillation vial. 0.5 mL of EtOH/H_2O (v/v = 1/2) was added and the mixture was sonicated for several minutes. The suspension formed has a concentration of 6.4 mg/mL. These are shown in Figure S6 a-b.

Gel preparation: Compound **9** (3.2 mg, 0.0026 mmol, 0.02 equiv.) and $CuSO_4$ pentahydrate (6.5 mg, 0.026 mmol, 0.2 equiv.) was added to a scintillation vial. 0.5 mL of EtOH/H_2O (v/v = 1/2) was added and the mixture was heated until the solid was completely dissolved. Upon cooling, stable gels formed. The gel formed has a concentration of 6.4 mg/mL. These are shown in Figure S6 c-e.

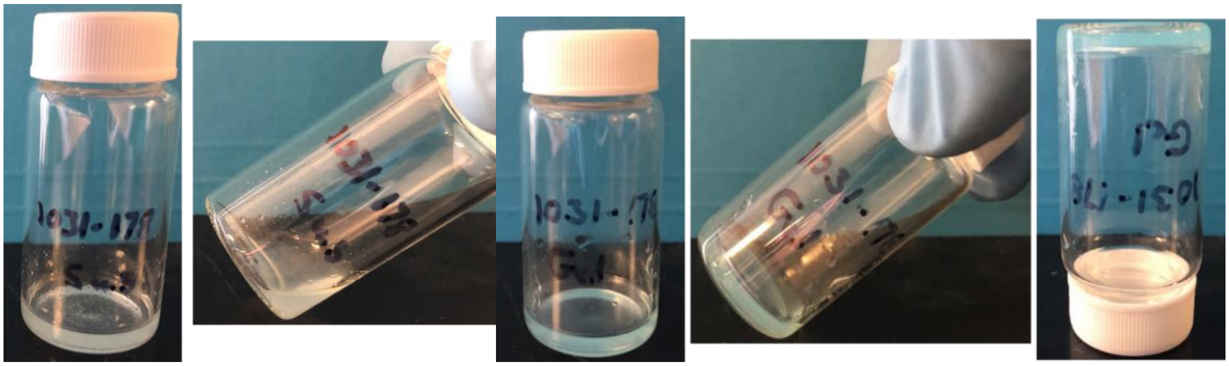

a b c d e

Figure S6. Suspension and gel preparation of compound **9**. a) Suspension made of compound **9** (3.2 mg) containing $CuSO_4$ pentahydrate (6.5 mg) in 0.5 mL of EtOH/H_2O (v/v = 1/2); b) tilted scintillation vial containing suspension. c) Gel made of compound **9** (3.2 mg) containing $CuSO_4$ pentahydrate (6.5 mg) in 0.5 mL of EtOH/H_2O (v/v = 1/2); d) tilted scintillation vial containing gel; e) inverted gel vial to show the stability of gel.

The experimental procedure of triazole **17** synthesis under suspension or gel condition followed by ^{1}H NMR spectra of the reaction monitoring and isolated final product are shown below:

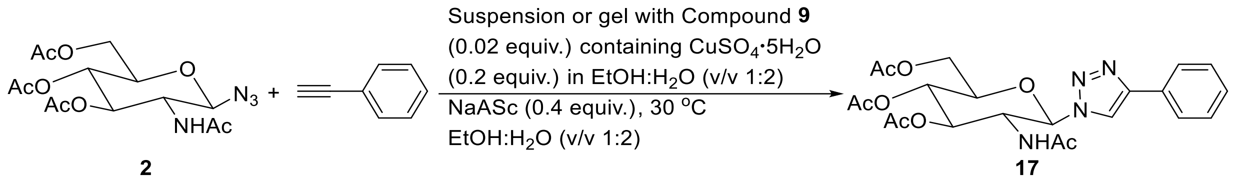

Scheme S2. Synthesis of compound **17** using compound **9** at different conditions

*Synthesis of triazole **17** in suspension (as control):* Sugar azide **2** (50 mg, 0.13 mmol, 1.0 equiv.), phenylacetylene (14 µL, 0.23 mmol, 1.2 equiv.) and L-ascorbate sodium salt (4 mg, 0.026 mmol, 0.2 equiv.) dissolved in 1 mL of EtOH/H_2O (v/v 1/2) was added to the suspension. The reaction mixture was then placed in a shaker set as 60 rpm and the reaction occurred at 30 °C. ^{1}H NMR was utilized to monitor reaction conversion. Reaction was checked at 30 minutes, 1 hour, 2 hours and 3 hours. At 3 hours, ^{1}H NMR indicated 95.8% conversion, then the reaction was removed from the shaker and solvent was removed under vacuum to afford the crude product, which was purified by column chromatography using pure DCM to 3% MeOH/DCM to give a white solid (54.2 mg, 85.1%) as the desired product.

*Synthesis of triazole **17** in gel:* Sugar azide **2** (50 mg, 0.13 mmol, 1.0 equiv.), phenylacetylene (14 µL, 0.23 mmol, 1.2 equiv.) and L-ascorbate sodium salt (4 mg, 0.026 mmol, 0.2 equiv.) dissolved in 1 mL of EtOH/H_2O (v/v 1/2) was added on top of the gel. The reaction mixture was then placed in a shaker set as 60 rpm and the reaction occurred at 30 °C. ^{1}H NMR was utilized to monitor reaction conversion. Reaction was checked at 30 minutes, 1 hour, 2 hours and 3 hours. Full reaction conversion was observed at 2 hours and the reaction mixture was removed from the shaker and

solvent was removed under vacuum to afford the crude product, which was purified by column chromatography using pure DCM to 3% MeOH/DCM to give a white solid (59.6 mg, 94%) as the desired product.

The images of the reaction vials for the preparation of compound **17** using compound **9** as ligand at different condition are shown in Figure S7: (a-b) Reaction done in suspension with suspension; c-d) reactions done using gels of compound **9**; (c-d) reactions done using gels of compound **9**.

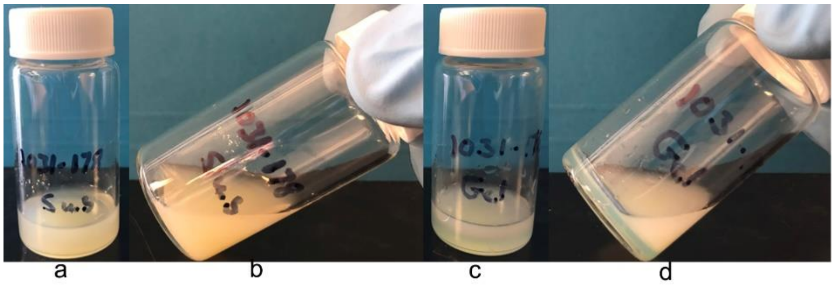

Figure S7. Sample vials for the synthesis of compound **17** using compound **9** as the ligand.

2.3. ^{1}H NMR spectra of the reaction monitoring and isolated final product using suspension

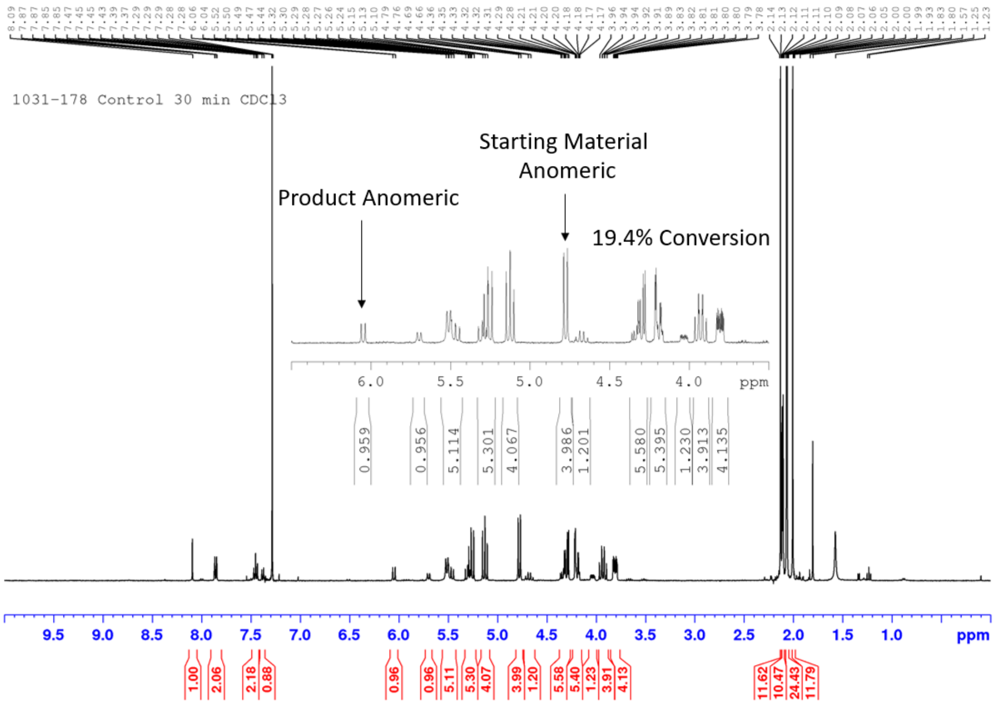

Figure S8. ^{1}H NMR spectrum ($CDCl_3$, 400 MHz) of the reaction using suspension at 30 minutes (19% conversion).

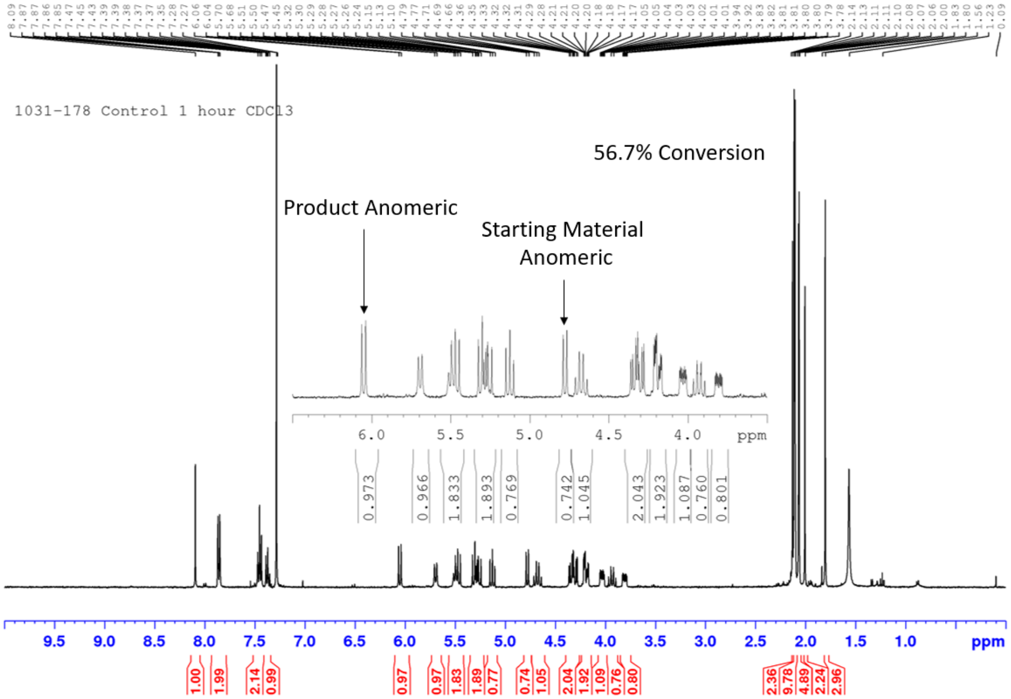

Figure S9. ^{1}H NMR spectrum ($CDCl_3$, 400 MHz) of the reaction using suspension at 1 hour (57% conversion).

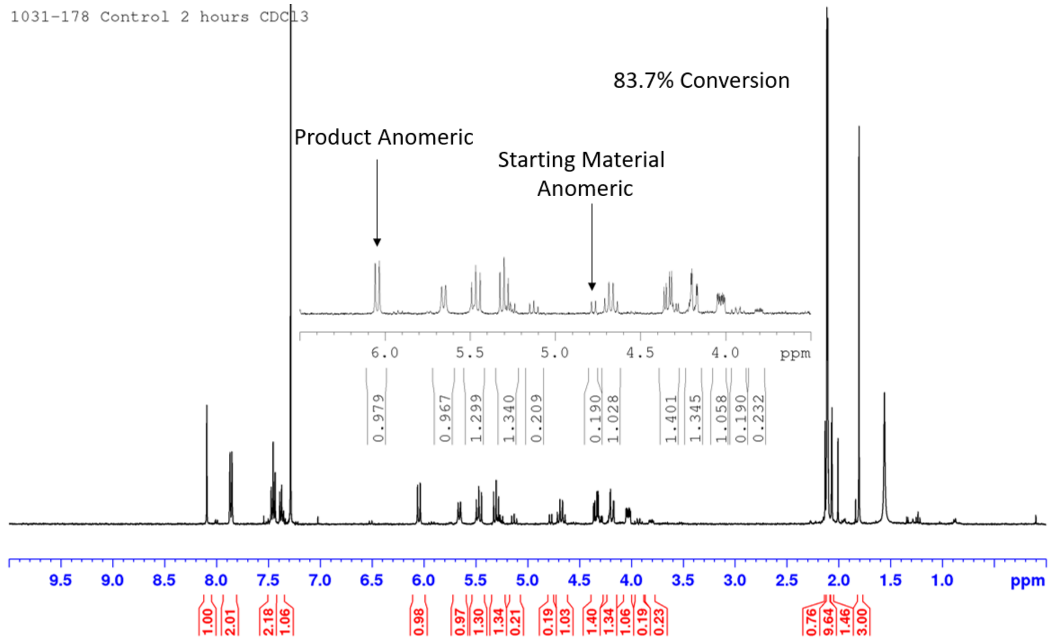

Figure S10. ^{1}H NMR spectrum ($CDCl_3$, 400 MHz) of the reaction using suspension at 2 hours (84% conversion).

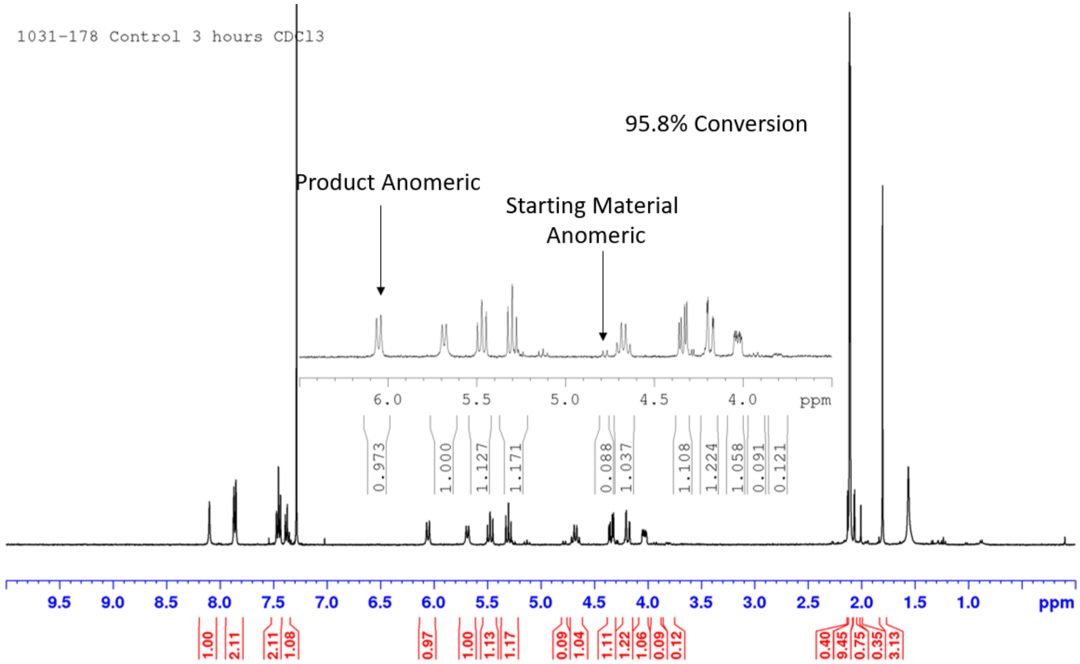

Figure S11. ^{1}H NMR spectrum ($CDCl_3$, 400 MHz) of the reaction using suspension at 3 hours (96% conversion).

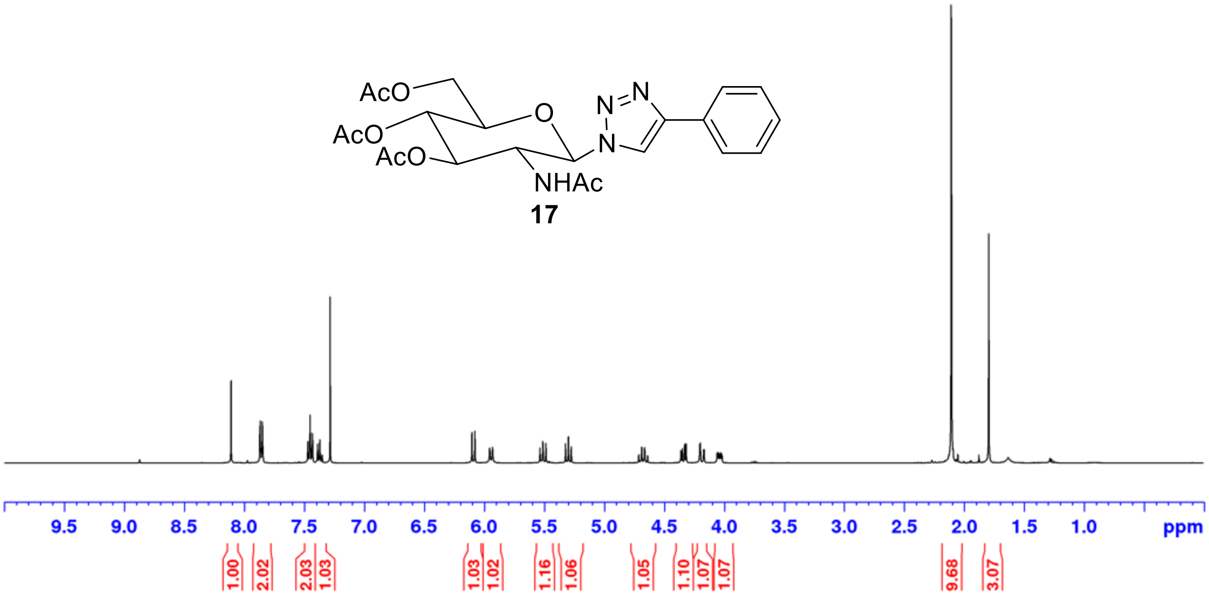

Figure S12. ^{1}H NMR spectrum ($CDCl_3$, 400 MHz) of compound **17** after column purification from the reaction using suspension.

2.4. ^{1}H NMR spectra of the reaction monitoring and isolated final product using gel

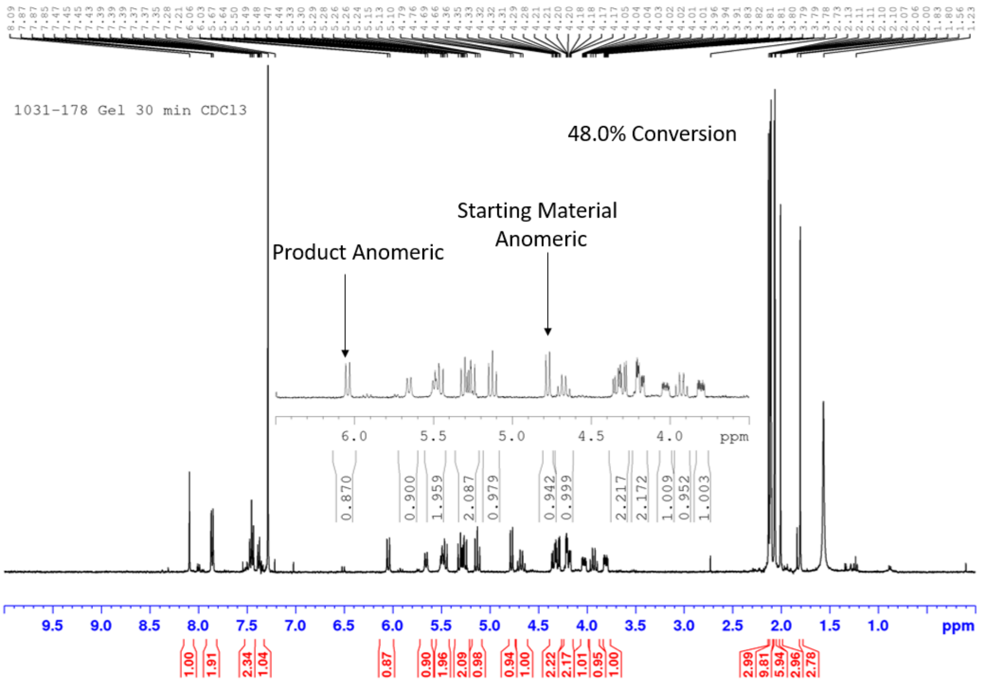

Figure S13. ^{1}H NMR spectrum ($CDCl_3$, 400 MHz) of the reaction using gel at 30 minutes (48% conversion).

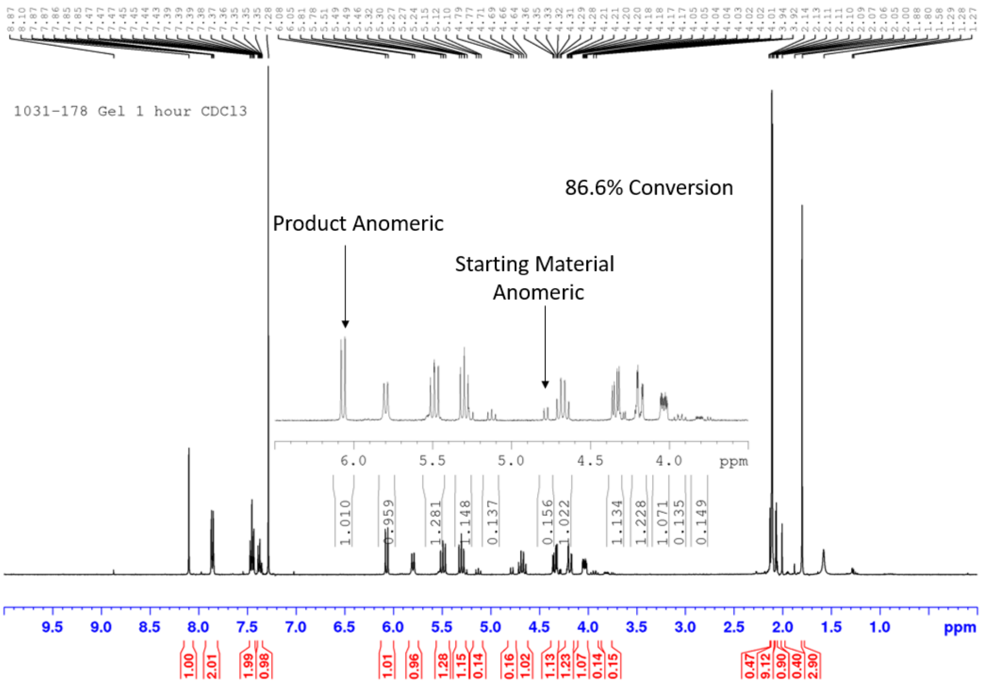

Figure S14. ^{1}H NMR spectrum ($CDCl_3$, 400 MHz) of the reaction using gel at 1 hour (87% conversion).

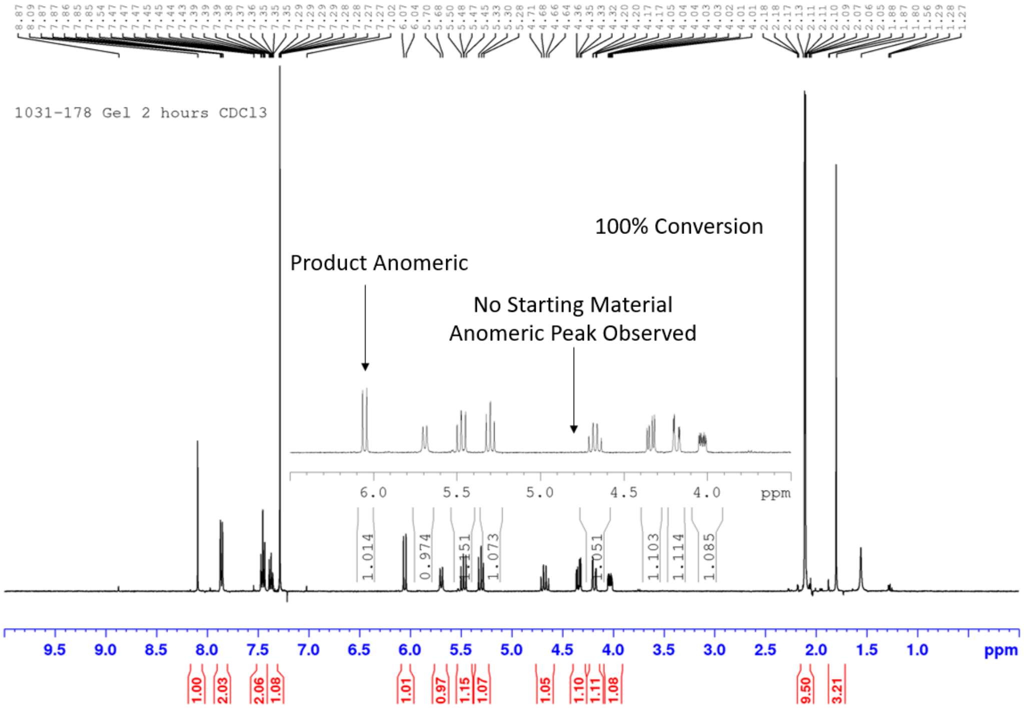

Figure S15. ^{1}H NMR spectrum ($CDCl_3$, 400 MHz) of the reaction using gel at 2 hours (full conversion).

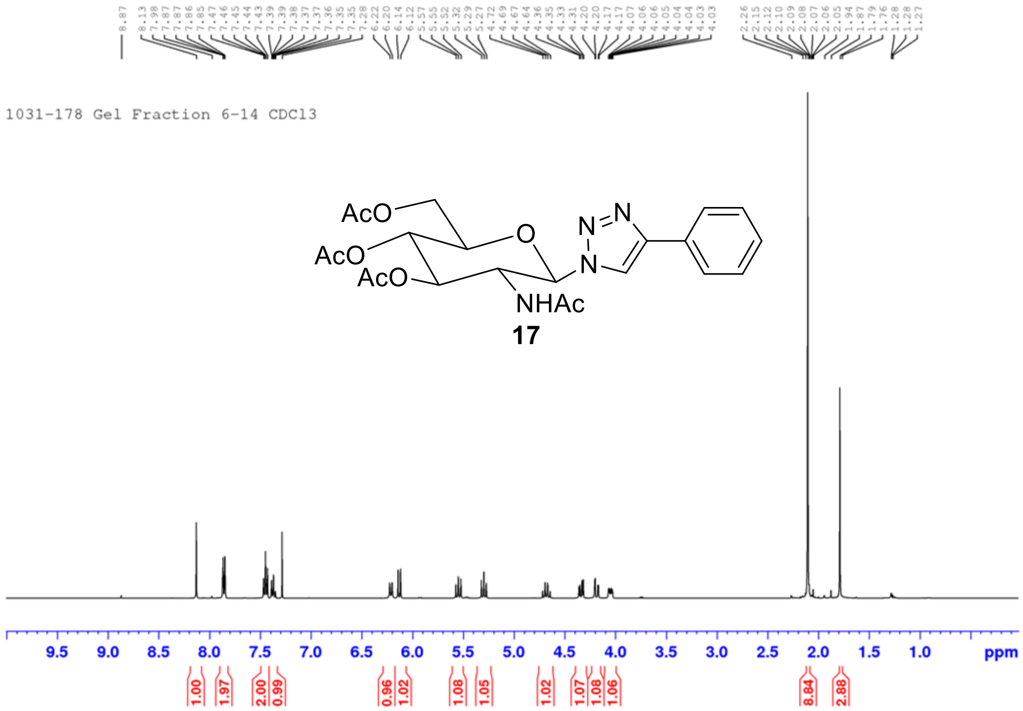

Figure S16. ^{1}H NMR spectrum ($CDCl_3$, 400 MHz) of compound **17** after column purification from the reaction using gel.

3. Experimental details of reactions on gel column of trimeric gelator **9**

3.1. Gel column preparation:

Compound **9** (10 mg, 0.008 mmol) was added to a 1-dram vial. 1.5 mL of EtOH/H_2O (v/v = 1/1) was added and the mixture was heated until the solid was completely dissolved. Upon cooling, stable gels formed. $CuSO_4$ pentahydrate (6 mg, 0.022 mmol) was then added on top of the gel and the vial was gently heated until the solids were dissolved. Upon cooling a stable clear gel was formed (Before the addition of copper, the gel was opaque). The gel was then re-heated into solution phase and added to an inverted syringe with a plug. Upon cooling a gel formed within the inverted syringe.

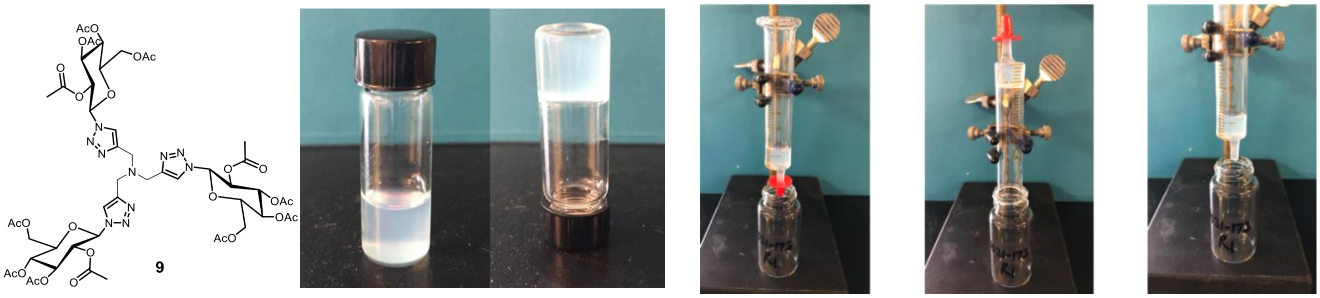

(a) (b) (c) (d) (e)

Figure S17. a) Gel made of compound **9** (10 mg) containing $CuSO_4$ pentahydrate (6 mg) in 1.5 mL of EtOH/H_2O (v/v = 1/2) ; b) reversed gel vial to show the stability of gel; c) gel column A made of compound **9** (10 mg) containing $CuSO_4$ pentahydrate (6 mg, 0.022 mmol) in 1.5 ml of EtOH/H_2O (v/v = 1/2) with the cap on, d) reversed gel column to show the stability of gel with a cap on; e) gel column without a cap on.

3.2. Synthesis of trizole compoound **19** using gel column A

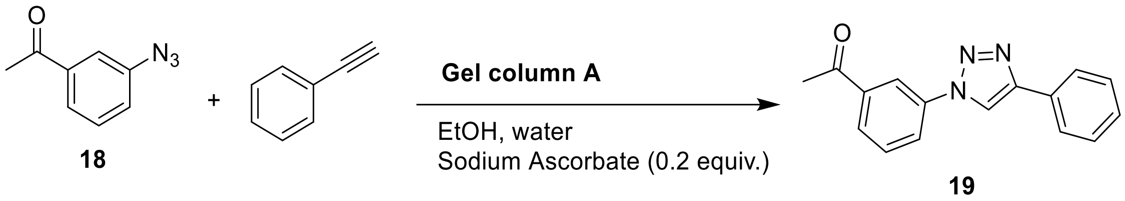

Scheme S3. Synthesis of compound **19** using the copper metallogel column made by compound **9**

A solution of phenylacetylene (30 µL, 0.27 mmol, 1.2 equiv.), 3-acetyl phenylazide (35 mg, 0.22 mmol, 1 equiv.) and L-ascorbate sodium salt (9 mg, 0.044 mmol, 0.2 equiv.) dissolved in EtOH/water (1 mL, v/v = 1/1) was added on top of the gel column. The reaction mixture was allowed to sit on top of the gel column for 1 hour. The cap was then removed, and elution occurred over a 15-minute period of time. After the elution was complete, EtOH/H_2O (1 mL, v/v = 1/1) was added to the top of the gel to push the compound off the gel column. 0.5 mL of EtOH/H_2O (v/v = 2/1) was then added to the gel column and allowed to elute, followed by another addition of 0.5 mL of EtOH/H_2O (v/v = 1/1) to prepare the column for the next round of reaction mixture. After five rounds of reaction mixtures, the gel column was flushed with 3 mL of EtOH. Crude products was collected in a scintillation vial and solvent was removed under reduced pressure producing a yellow solid. The crude products were then dissolved in DCM and pushed through a SiO_2 plug using DCM. The DCM was removed under reduced pressure leaving a light-yellow solid. The weight of the product after each round (in total 5 rounds) and the flush round is 34.1 mg, 40.1 mg, 43.2 mg, 38.9 mg, 37.8 mg and 63.3 mg, respectively. Combined weight is 257.4 mg, therefore the yield of this reaction is 88.9%.

The following section includes the images of gel column for each cycle, followed by ^{1}H NMR spectra of product **19** (all in $CDCl_3$, 400 MHz) before and after SiO_2 plug treatment. Total five rounds or cycles of reagent loading, and one cycle of final flush were carried out.

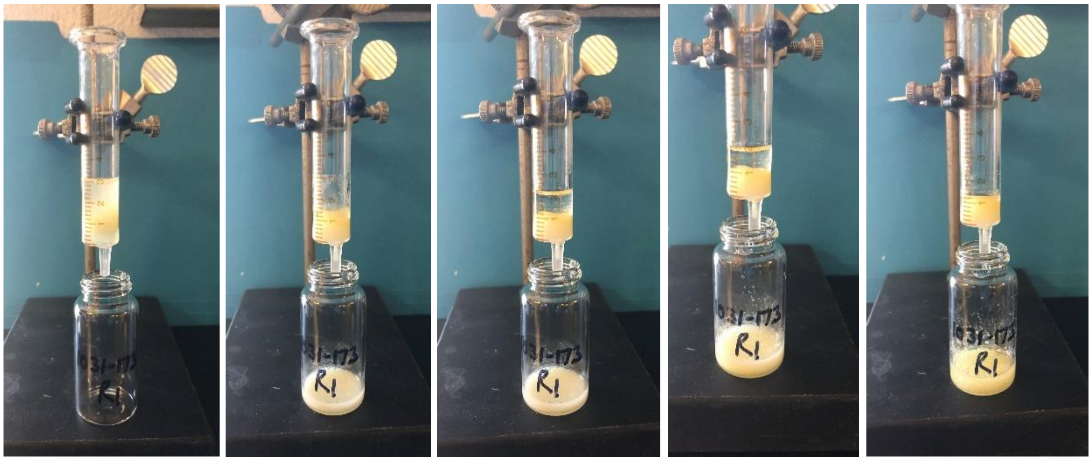

a b c d e

Figure S18. The first cycle of the experiment of compound **19** synthesis using gel column A. a) Reagents loaded to the gel column before the elution; b) gel column after the elution; c) 1 mL of EtOH/H_2O (v/v = 2/1) to wash the gel column; d) 0.5 mL of EtOH/H_2O (v/v = 1/1) to wash the gel column; e) gel column after round 1 experiment completed.

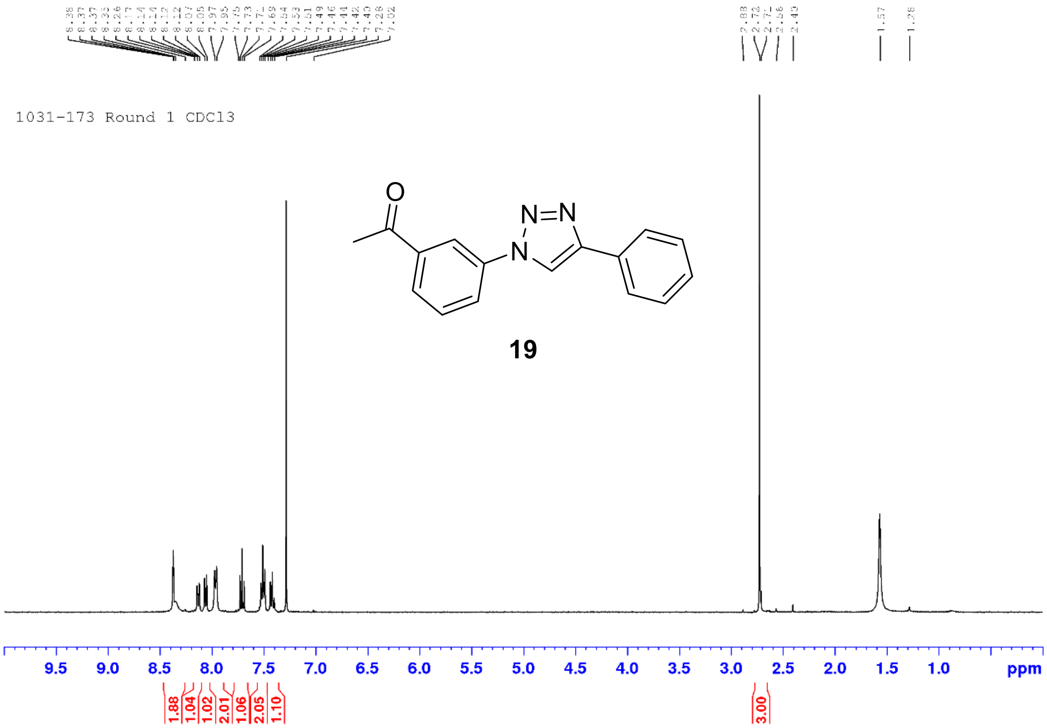

Figure S19. ^{1}H NMR spectrum ($CDCl_3$, 400 MHz) of crude product (**Round 1**).

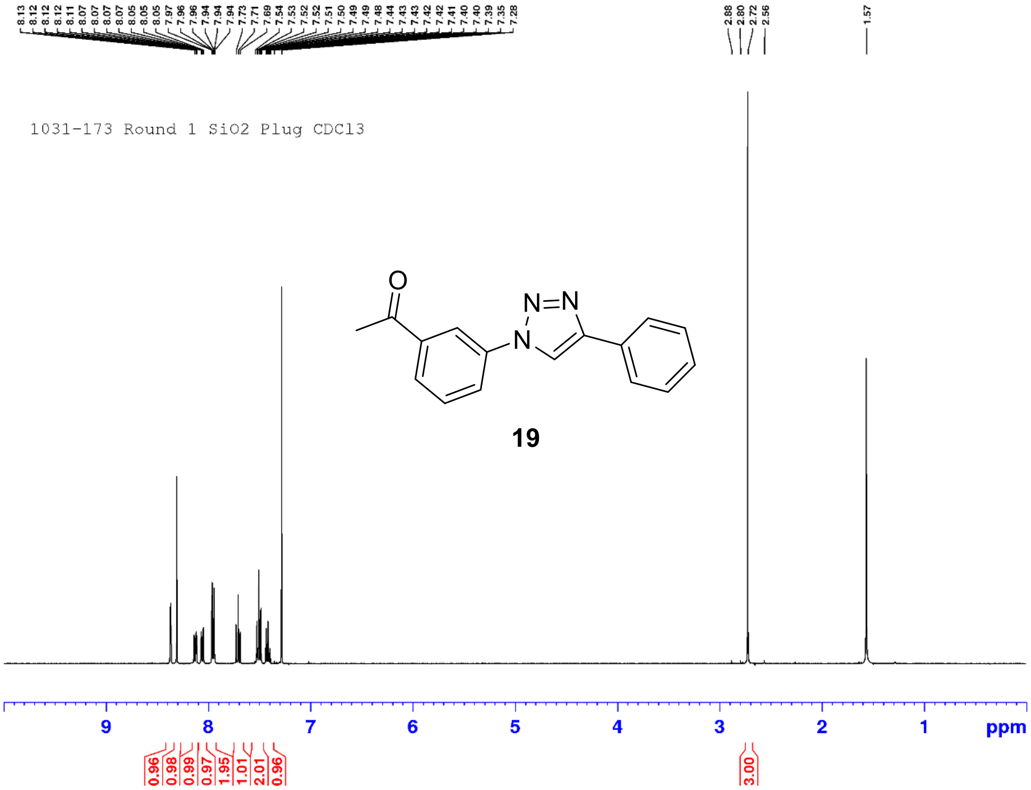

Figure S20. ^{1}H NMR spectrum ($CDCl_3$, 400 MHz) of compound **19** after SiO_2 plug treatment (**Round 1**).

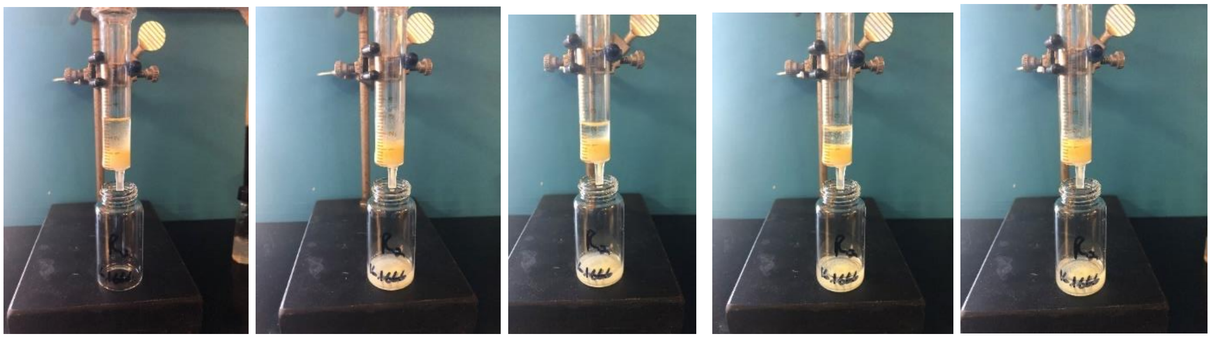

a b c d e

Figure S21. The second cycle of the experiment of compound **19** synthesis using gel column a) Reagents loaded to the gel column before the elution; b) gel column after the elution; c) 1 mL of EtOH/H_2O (v/v = 2/1) to wash the gel column; d) 0.5 mL of EtOH/H_2O (v/v = 1/1) to wash the gel column; e) gel column after round 2 experiment completed.

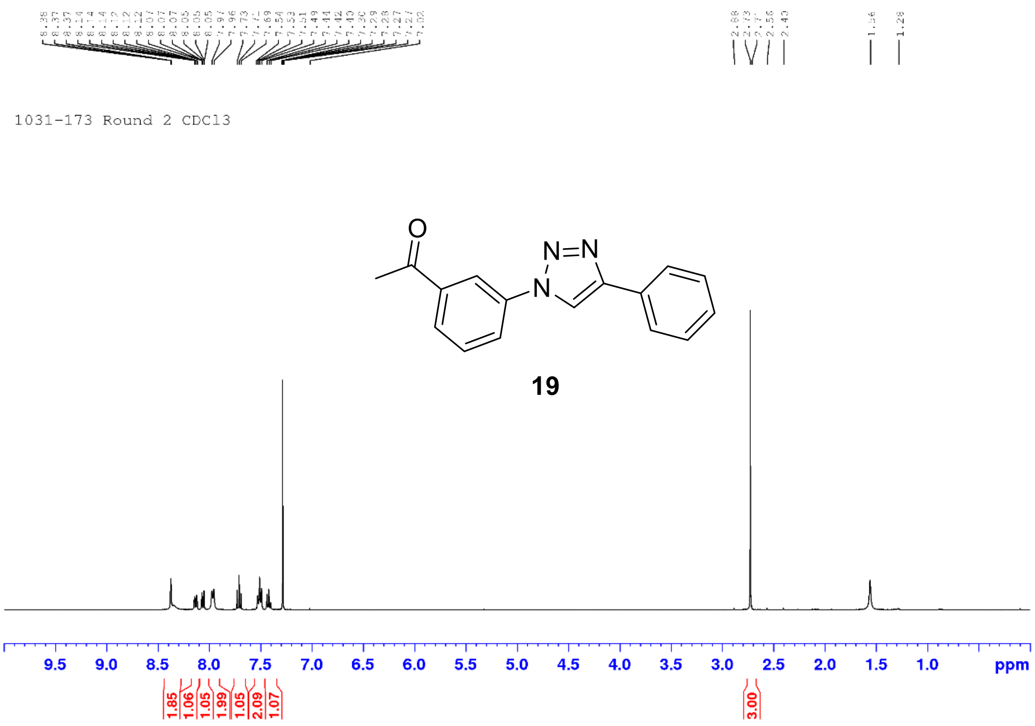

Figure S22. ^{1}H NMR spectrum ($CDCl_3$, 400 MHz) of crude product (**Round 2**).

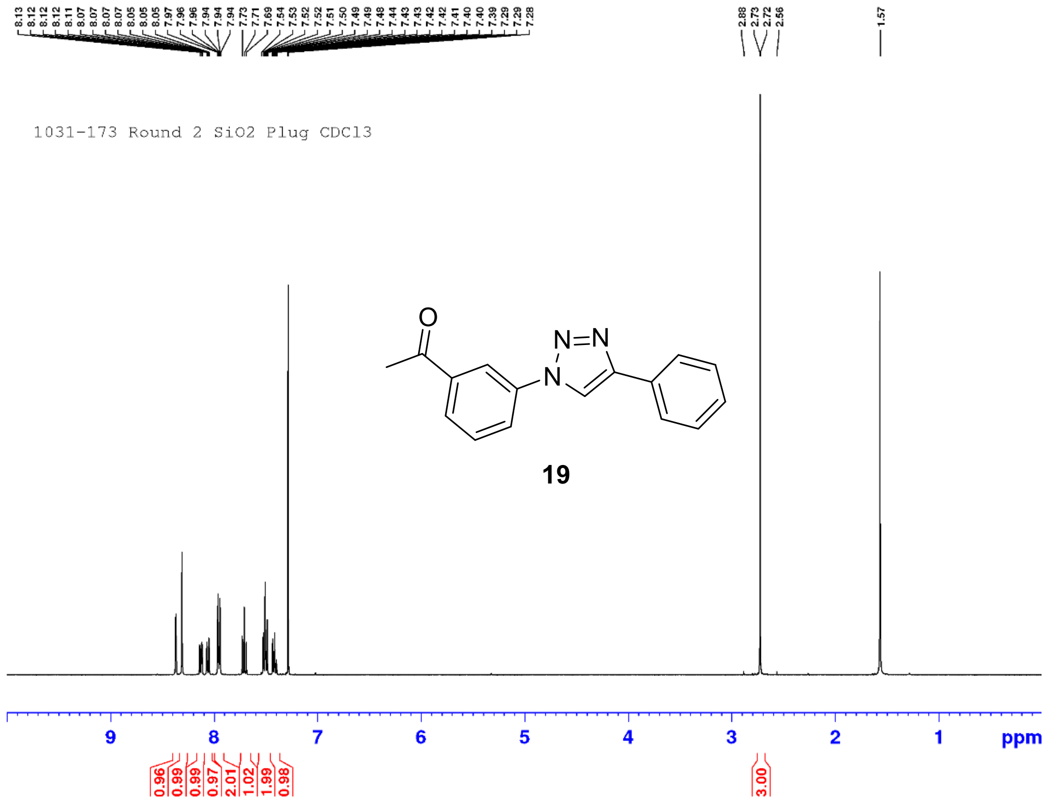

Figure S23. ^{1}H NMR spectrum ($CDCl_3$, 400 MHz) of compound **19** after SiO_2 plug treatment (**Round 2**).

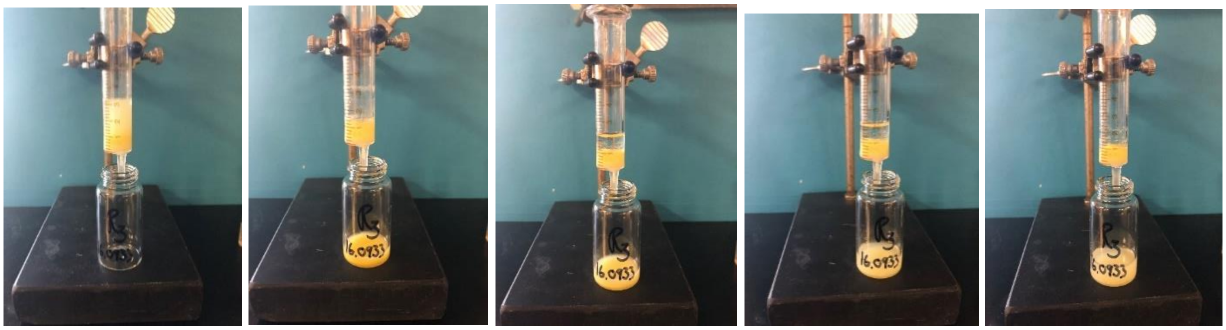

a b c d e

Figure S24. The third cycle of the experiment of compound **19** synthesis using gel column a) Reagents loaded to the gel column before elution; b) gel column after the elution; c) 1 mL of EtOH/H_2O (v/v = 2/1) to wash the gel column; d) 0.5 mL of EtOH/H_2O (v/v = 1/1) to wash the gel column; e) gel column after round 3 experiment completed.

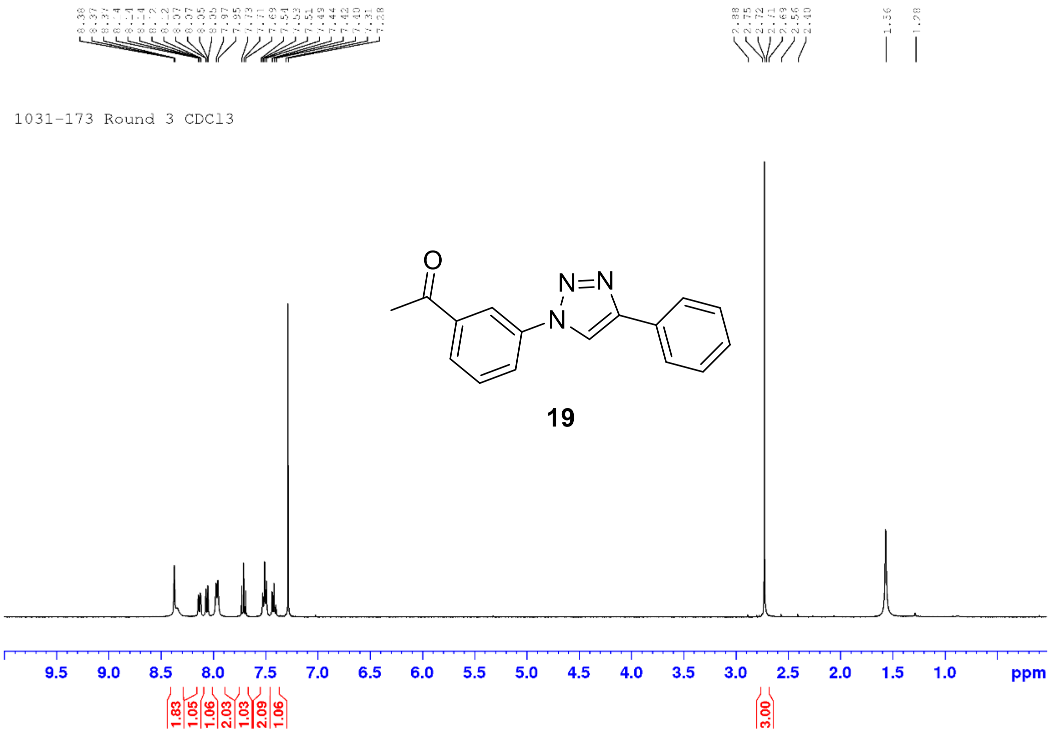

Figure S25. ^{1}H NMR spectrum ($CDCl_3$, 400 MHz) of crude product (**Round 3**).

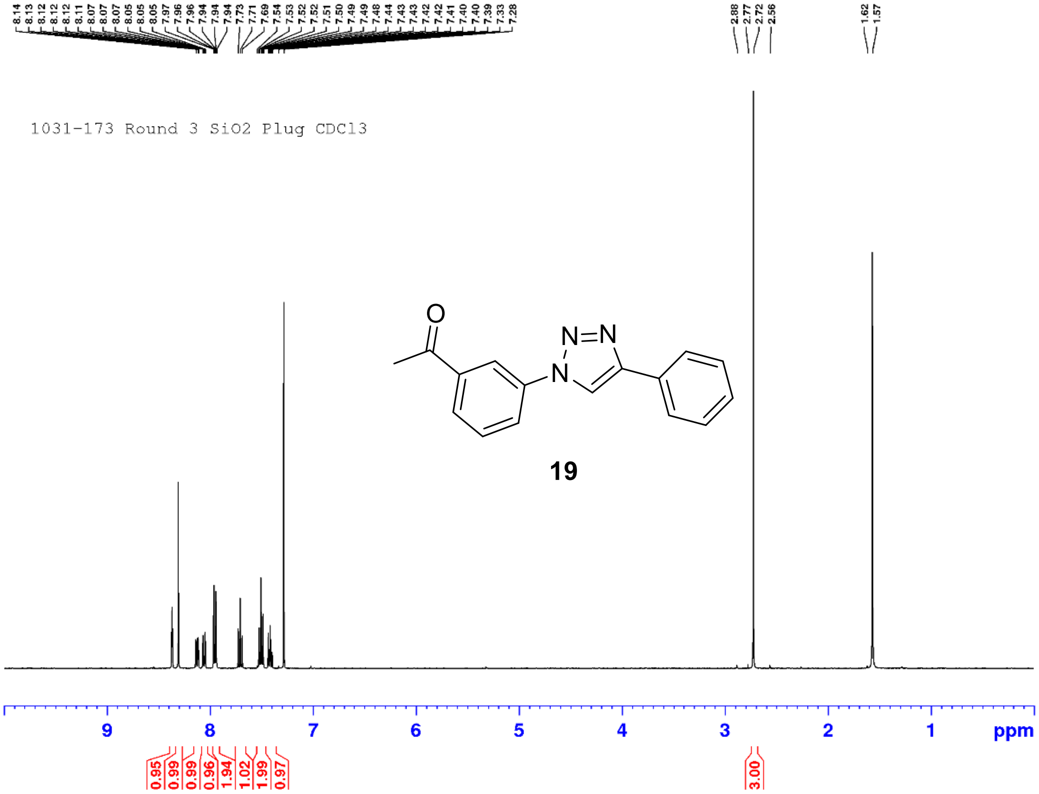

Figure S26. ^{1}H NMR spectrum ($CDCl_3$, 400 MHz) of compound **19** after SiO_2 plug treatment (**Round 3**).

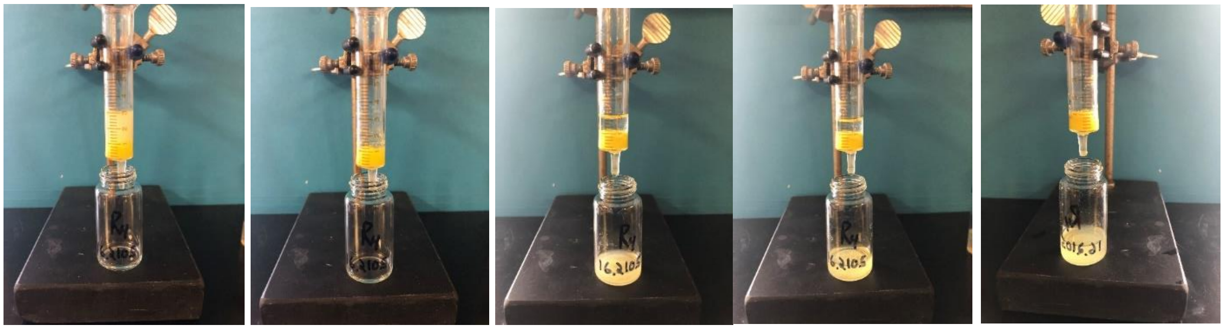

a b c d e

Figure S27. The fourth cycle of the experiment of compound **19** synthesis using gel column a) Reagents loaded to the gel column before the elution; b) gel column after the elution; c) 1 mL of EtOH/H_2O (v/v = 2/1) to wash the gel column; d) 0.5 mL of EtOH/H_2O (v/v = 1/1) to wash the gel column; e) gel column after round 4 experiment completed.

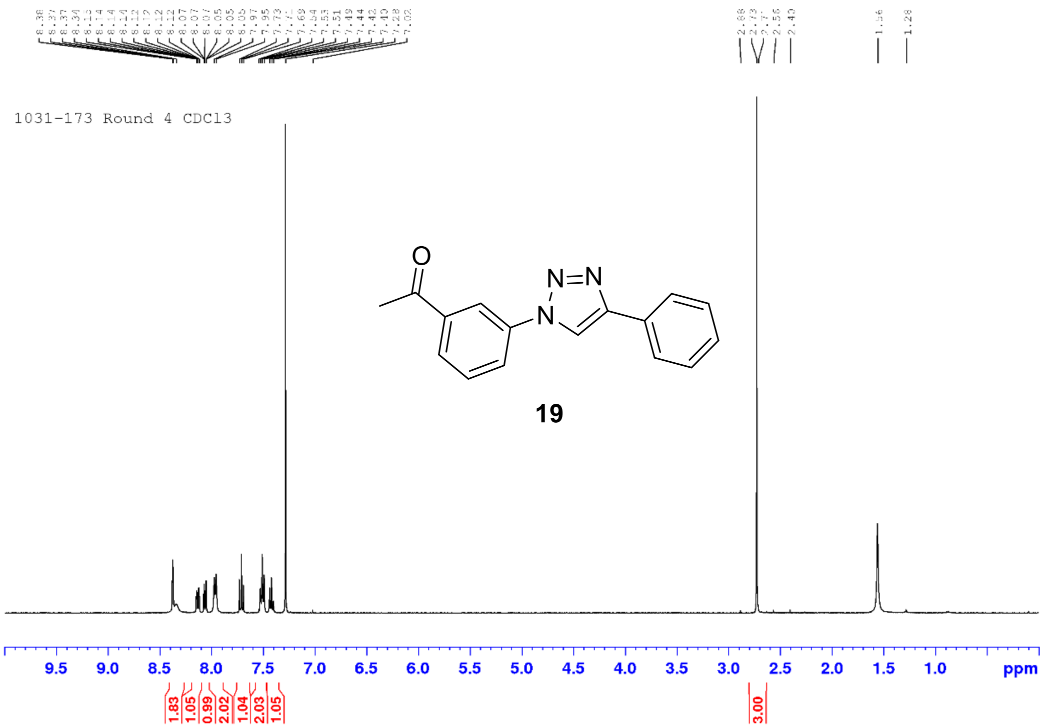

Figure S28. ^{1}H NMR spectrum ($CDCl_3$, 400 MHz) of crude product (**Round 4**).

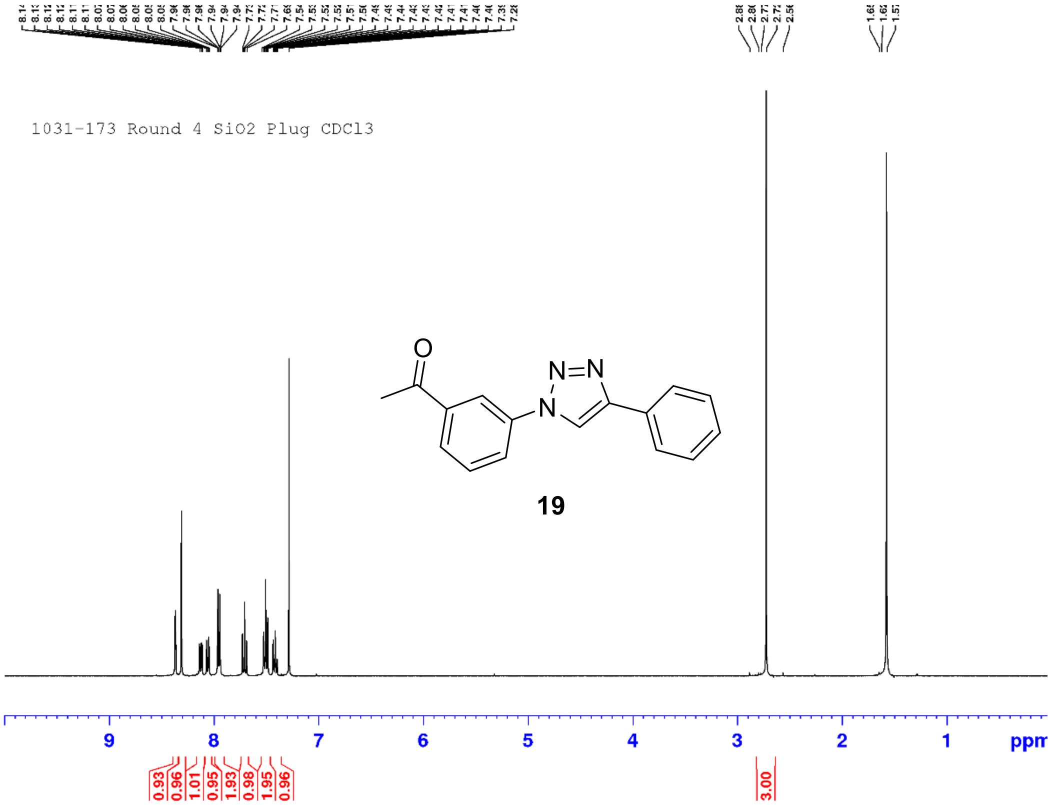

Figure S29. ^{1}H NMR spectrum ($CDCl_3$, 400 MHz) of compound **19** after SiO_2 plug treatment (**Round 4**).

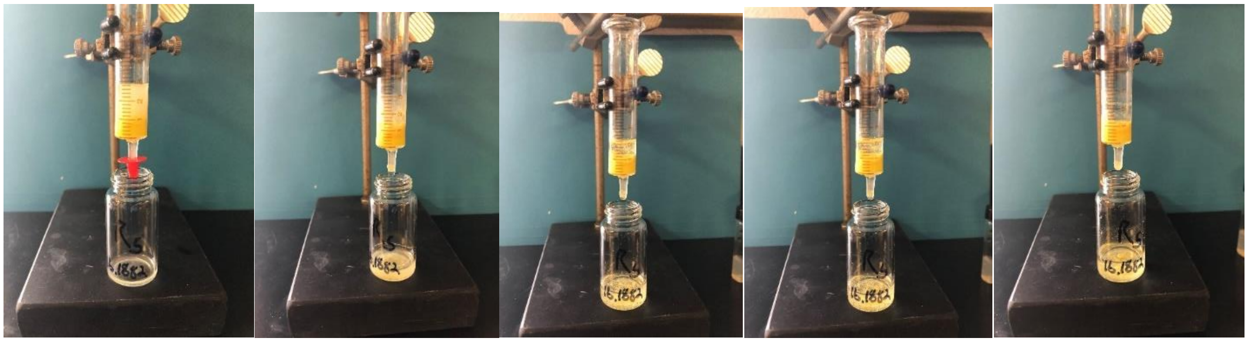

a b c d e

Figure S30. The fifth cycle of the synthesis of compound **19** using the gel column a) Reagents loaded to the gel column before the elution; b) gel column after the elution; c) 1 mL of EtOH/H_2O (v/v = 2/1) to wash the gel column; d) 0.5 mL of EtOH/H_2O (v/v = 1/1) to wash the gel column; e) gel column after round 5 experiment completed.

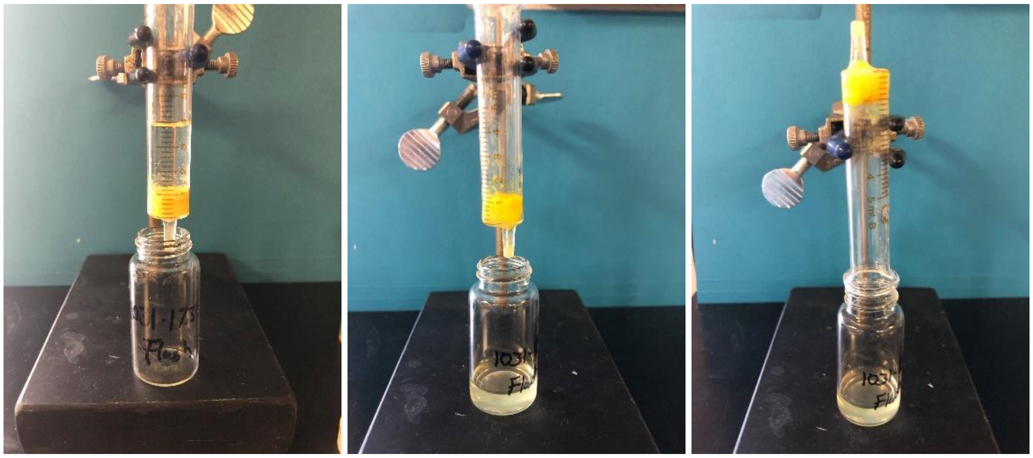

a b c

Figure S31. The final wash of the gel column after 5 cycles. a) 3 mL of EtOH loaded to the gel column before the elution; b) gel column after the elution; c) inverted gel column after the flush.

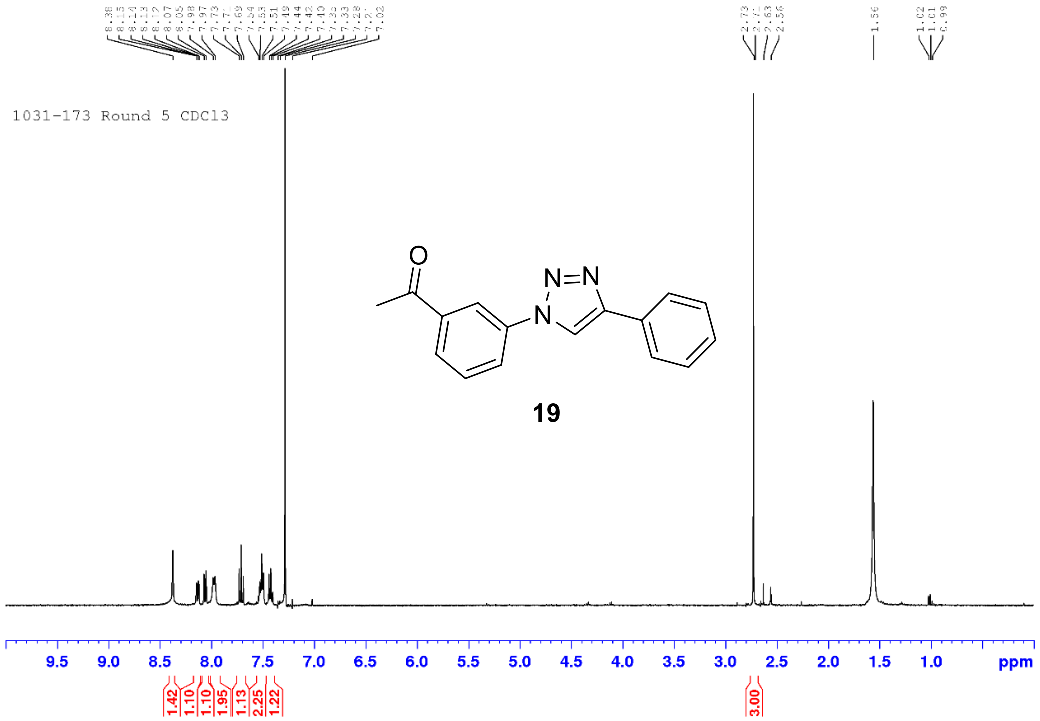

Figure S32. ^{1}H NMR spectrum ($CDCl_3$, 400 MHz) of crude product (**Round 5**).

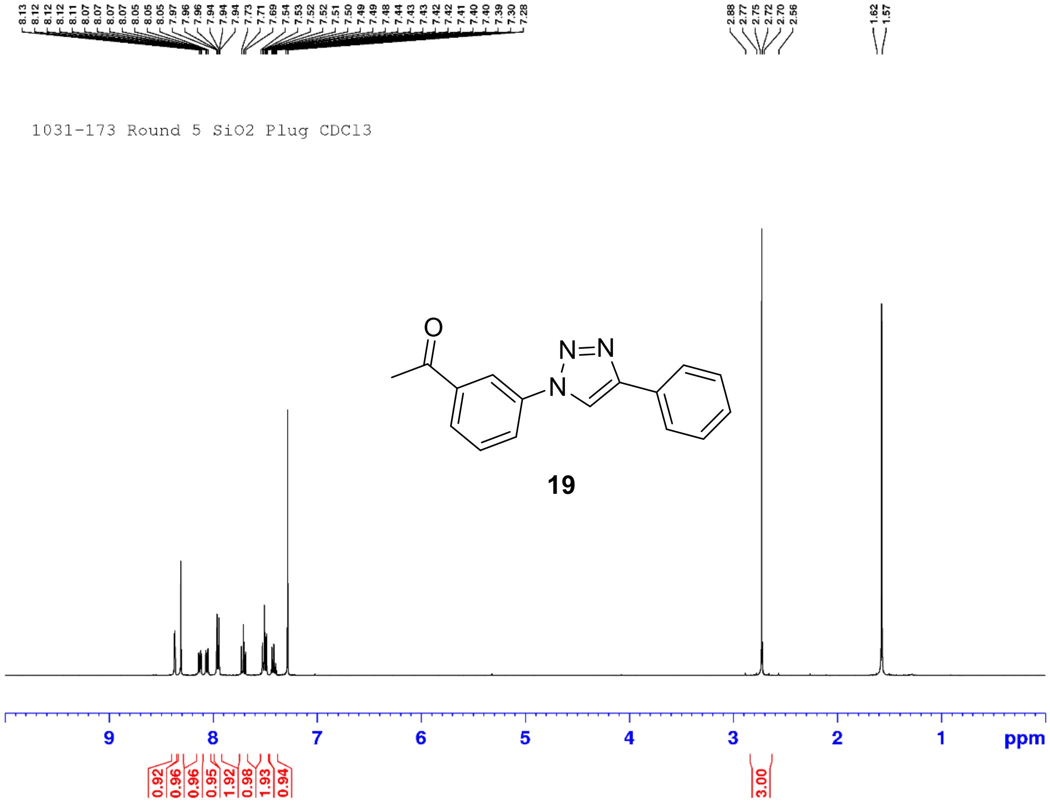

Figure S33. ^{1}H NMR spectrum ($CDCl_3$, 400 MHz) of compound **19** after SiO_2 plug treatment (**Round 5**).

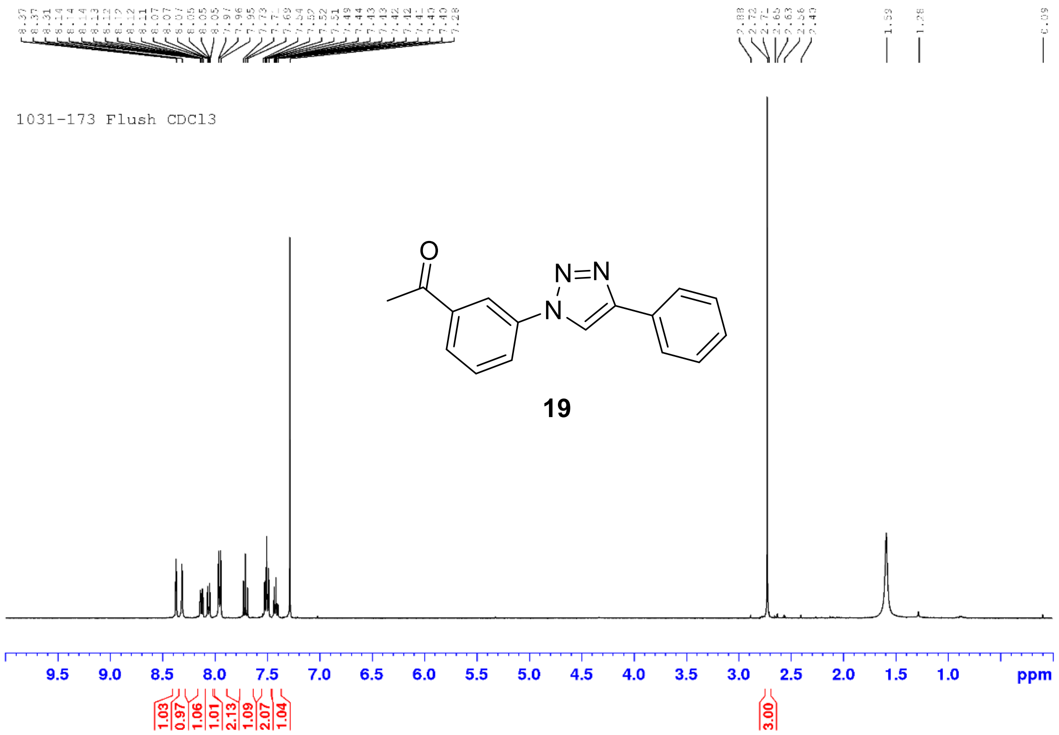

Figure S34. ^{1}H NMR spectrum ($CDCl_3$, 400 MHz) of crude product after final wash.

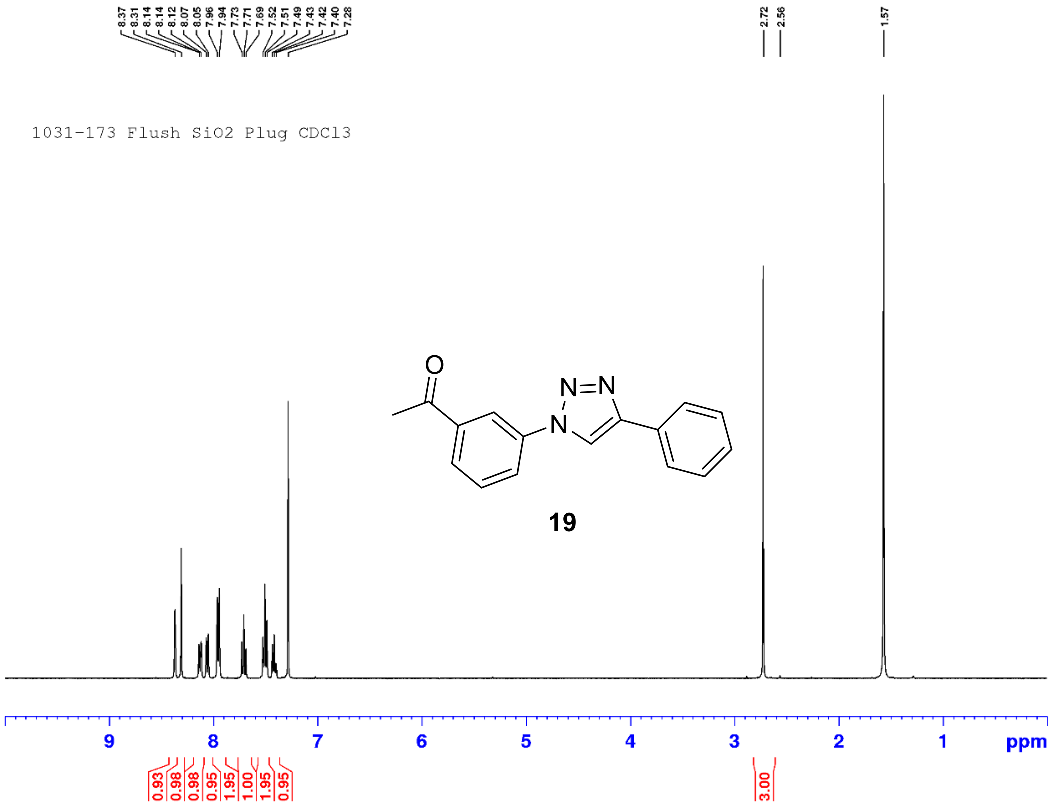

Figure S35. ^{1}H NMR spectrum ($CDCl_3$, 400 MHz) of compound **19** after SiO_2 plug treatment.

3.3. Synthesis of compoound **20** using the gel column - Reusability of the gel column catalyst

To test reusability of the gel column, another click reaction using sugar azide **1** and 1-octyne was carried out using the same gel column after the synthesis of compound **19** as shown in Figure S31e.

Experimental procedure of triazole **20** synthesis using gel column A:

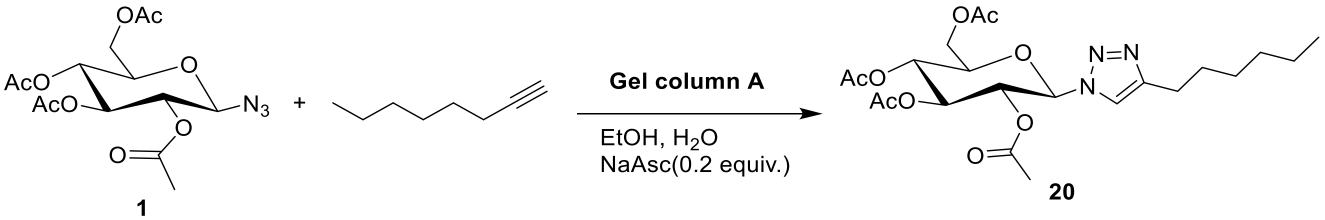

Scheme S4. Synthesis of compound **20** using the gel column A.

A stock solution of sugar azide **1** (100 mg, 0.268 mmol, 1.0 equiv.), 1-octyne (24 µL, 0.322 mmol, 1.2 equiv.) and L-ascorbate sodium salt (12 mg, 0.054 mmol, 0.2 equiv.) in 4 mL of EtOH/H_2O (v/v 1/1) was prepared. 2 mL of this solution, which contains sugar azide **1** (50 mg, 0.134 mmol, 1.0 equiv.), 1-octyne (12 µL, 0.161 mmol, 1.2 equiv.) and L-ascorbate sodium salt (6 mg, 0.027 mmol, 0.2 equiv.) was added on top of the gel column. The reaction mixture was allowed to sit on top of the gel column for 1 hour. The cap was then removed, and elution occurred over a 15-minute period of time. After the elution was complete, 1.0 mL of EtOH/H_2O (v/v = 1/1) was added to the top of the gel to push the compound off the gel column. 0.5 mL of EtOH/H_2O (v/v = 2/1) was then added to the gel column and allowed to elute, followed by another addition of 0.5 mL of EtOH/H_2O (v/v = 1/1) to prepare the column for the next round of reaction mixture. After the second experiment, 5 mL of pure EtOH was used to flush the gel column. Crude product was collected in scintillation vials and solvent was removed under reduced pressure. Crude mixtures were dissolved in 1% MeOH/DCM and pushed through a SiO_2 plug. For each sample, about 30 mL of 1% MeOH/DCM was required to fully get the sample off the SiO_2 plug. The weight of the product

after each round (in total 2 rounds) and the flush round is 47.1 mg, 49.7 mg and 25.4 mg respectively. Combined weight is 122.2 mg, therefore the yield of this reaction is 94.3%.

The following section includes the photos of gel column for the synthesis of compound **20** and the ^{1}H NMR spectra (all in $CDCl_3$, 400 MHz) before and after SiO_2 plug treatment of 2 cycles and 1 flush experiments.

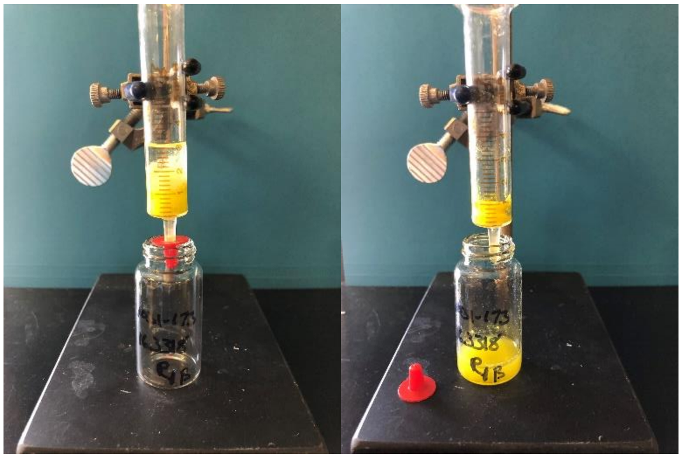

a b

Figure S36. The first cycle of the synthesis of compound **20** on the gel column A. a) Reagents loaded to the gel column before the elution; b) gel column after round 1 experiment completed.

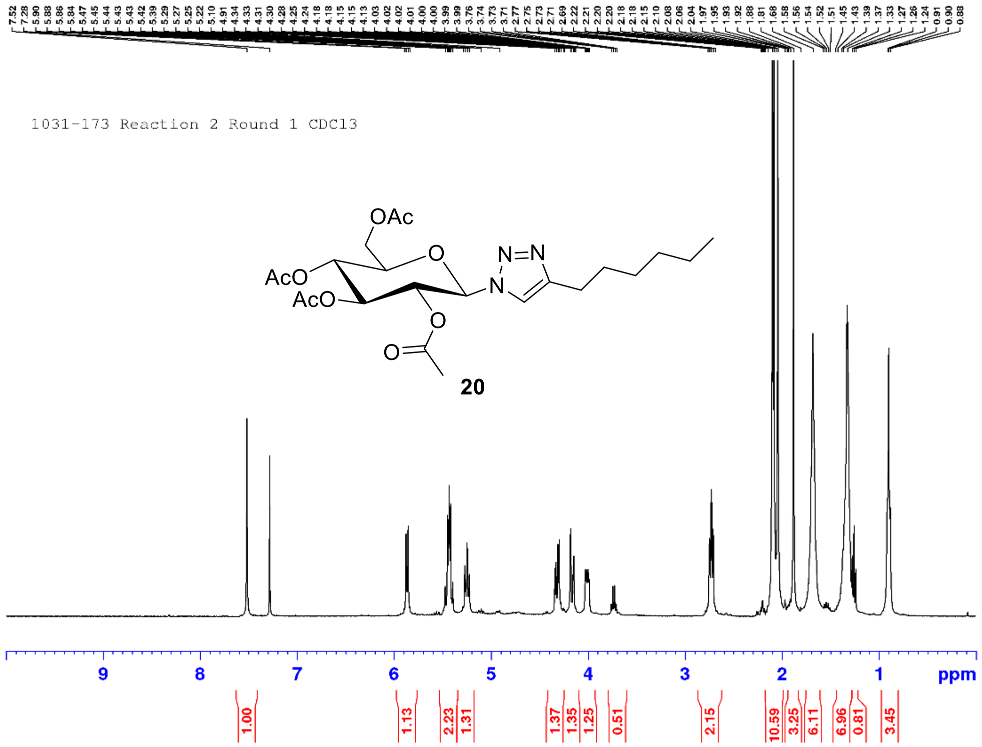

Figure S37. ^{1}H NMR spectrum ($CDCl_3$, 400 MHz) of crude product (**Round 1**).

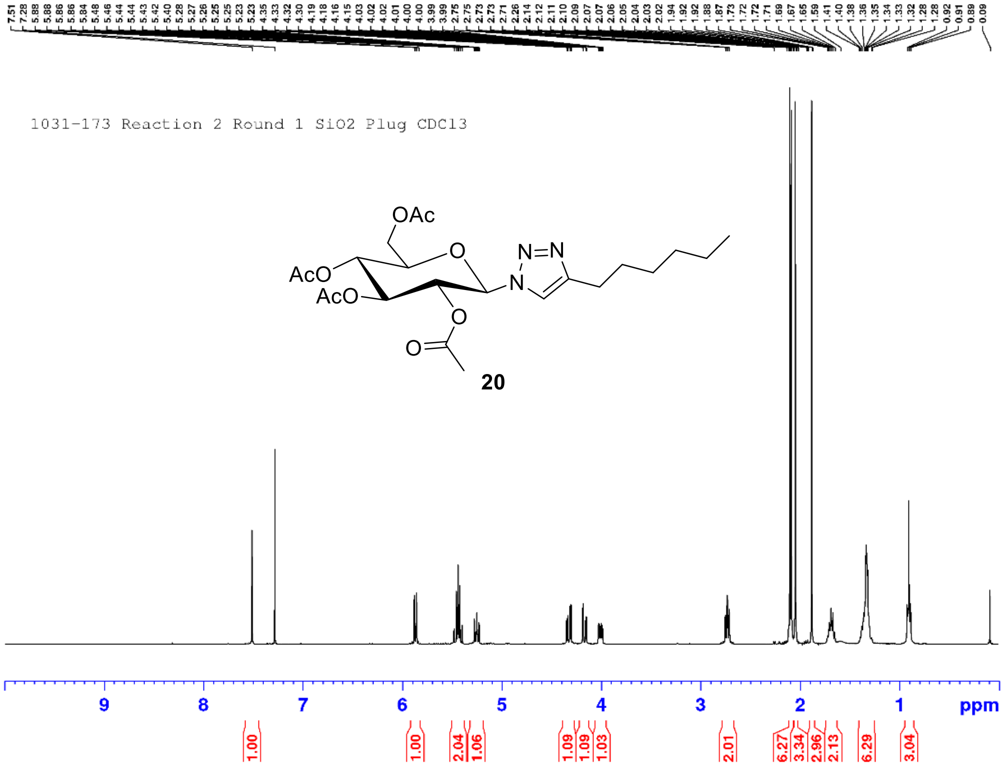

Figure S38. ^{1}H NMR spectrum ($CDCl_3$, 400 MHz) of compound **20** after SiO_2 plug treatment (**Round 1**).

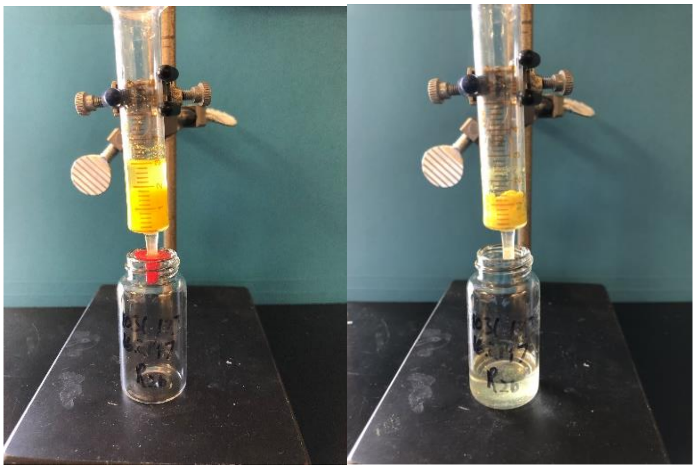

a b

Figure S39. The second cycle of the synthesis of compound **20** using the gel column: a) Reagents loaded to the gel column before the elution; b) gel column after round 2 experiment completed.

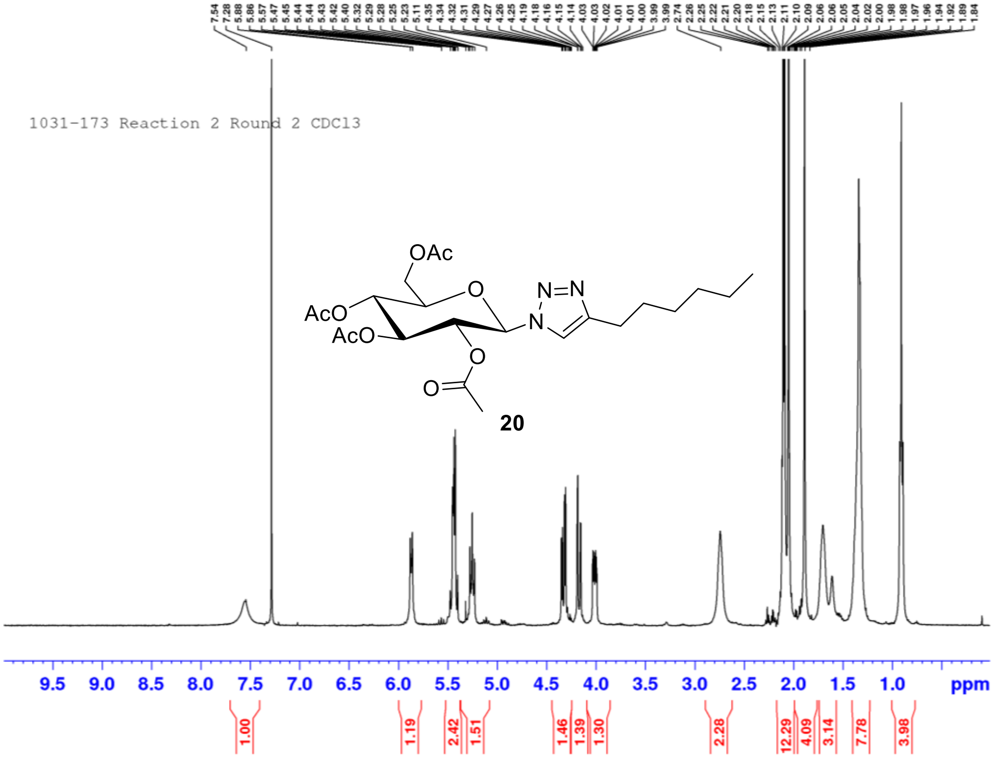

Figure S40. ^{1}H NMR spectrum ($CDCl_3$, 400 MHz) of crude product (**Round 2**).

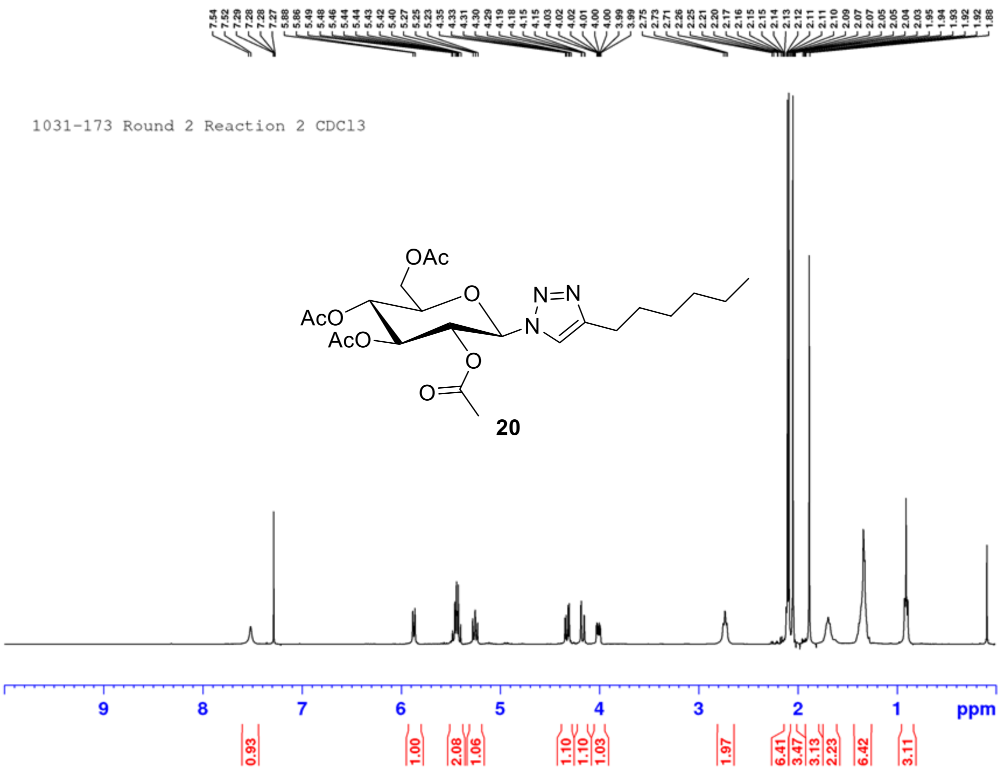

Figure S41. ^{1}H NMR spectrum ($CDCl_3$, 400 MHz) of compound **20** after SiO_2 plug treatment (**Round 2**).

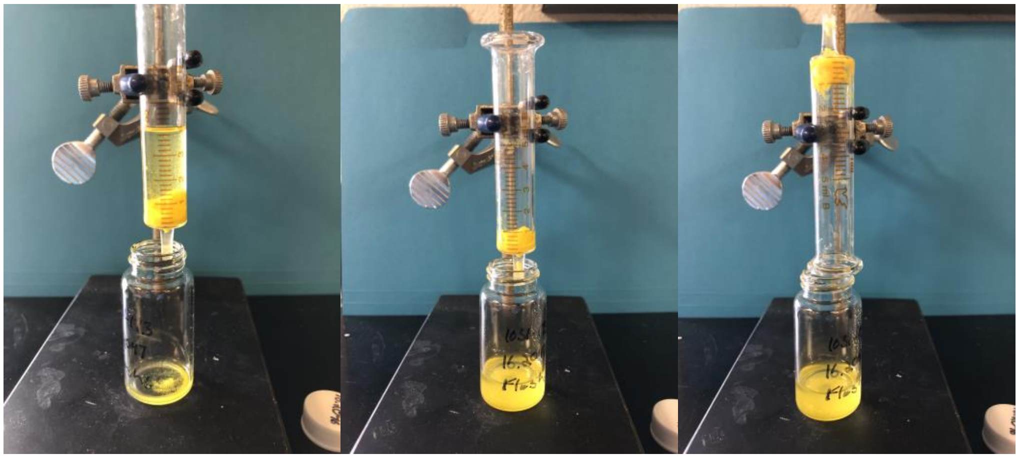

a b c

Figure S42. The images of the gel column after cycle 2 and followed by ethanol wash: a) 5 mL of EtOH loaded to the gel column before the elution; b) gel column after the elution; c) inverted gel column after flush. The ^{1}H NMR spectra are shown in Figures S43-44.

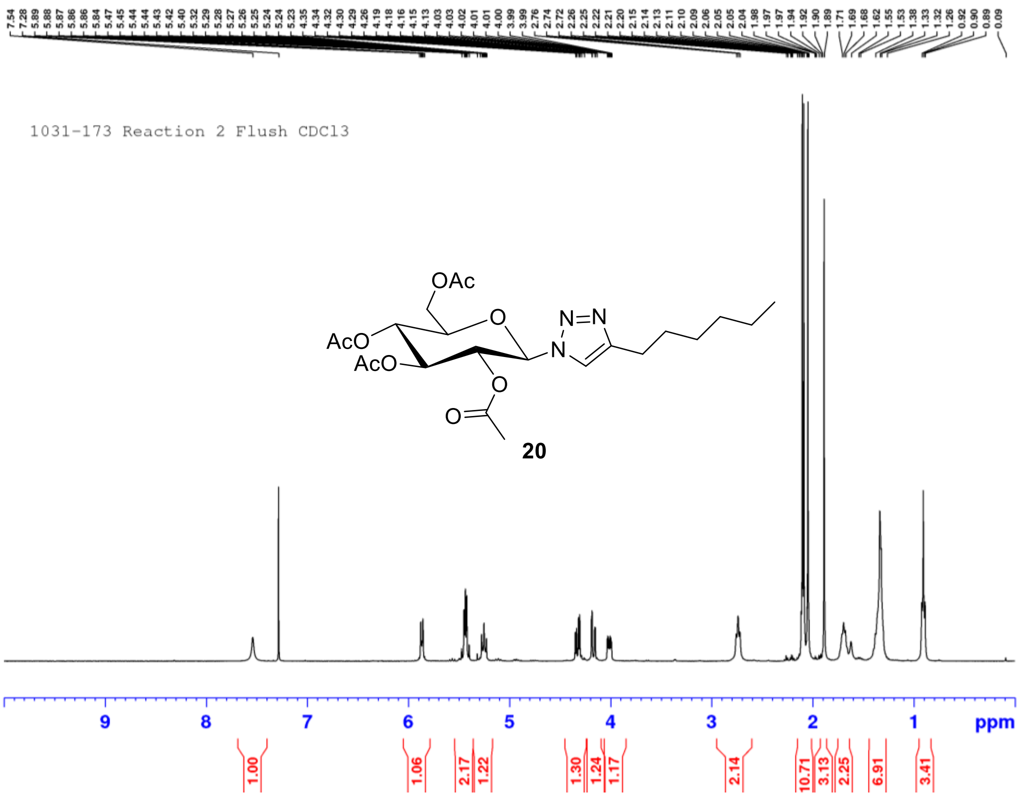

Figure S43. ^{1}H NMR spectrum ($CDCl_3$, 400 MHz) of crude product collected by flushing.

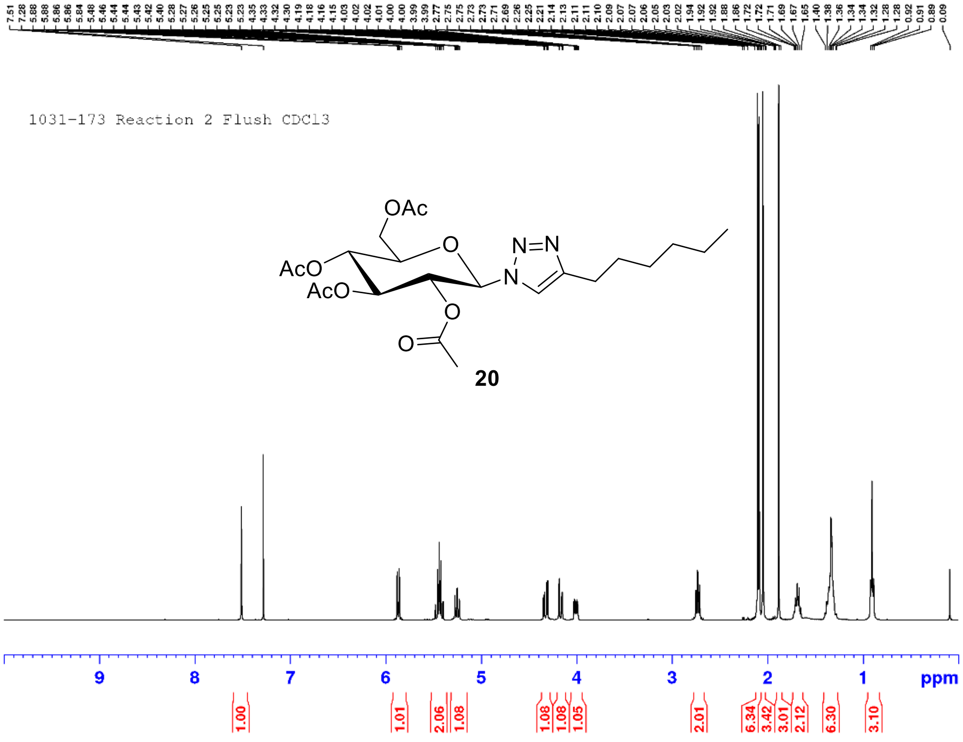

Figure S44.^{1}H NMR spectrum ($CDCl_3$, 400 MHz) of compound **20** after SiO_2 plug treatment.

Part III. ^{1}H and ^{13}C NMR spectra of compounds 8-16

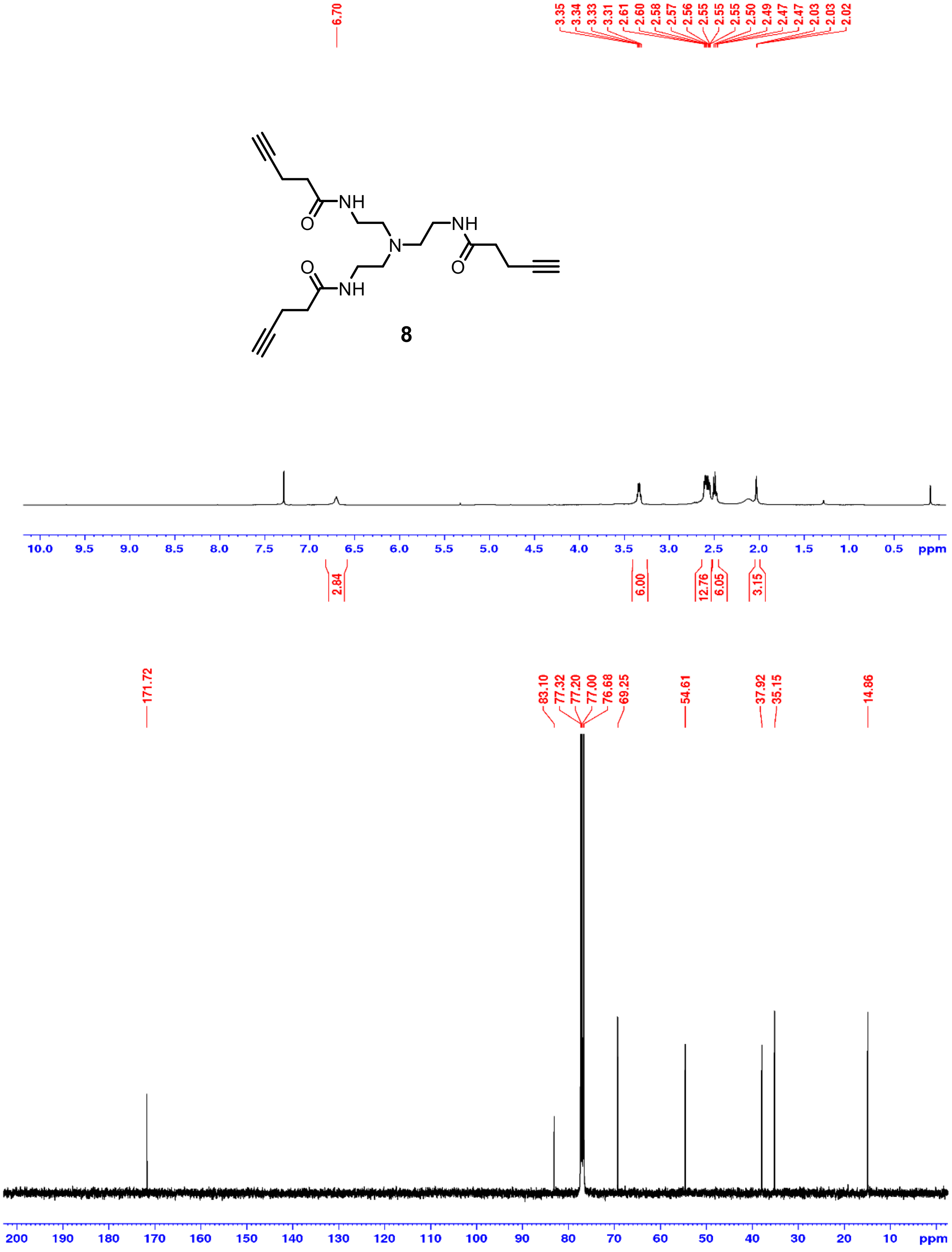

Figure S45. ^{1}H ($CDCl_3$, 400 MHz) and ^{13}C ($CDCl_3$, 100 MHz) NMR spectra of compound **8**.

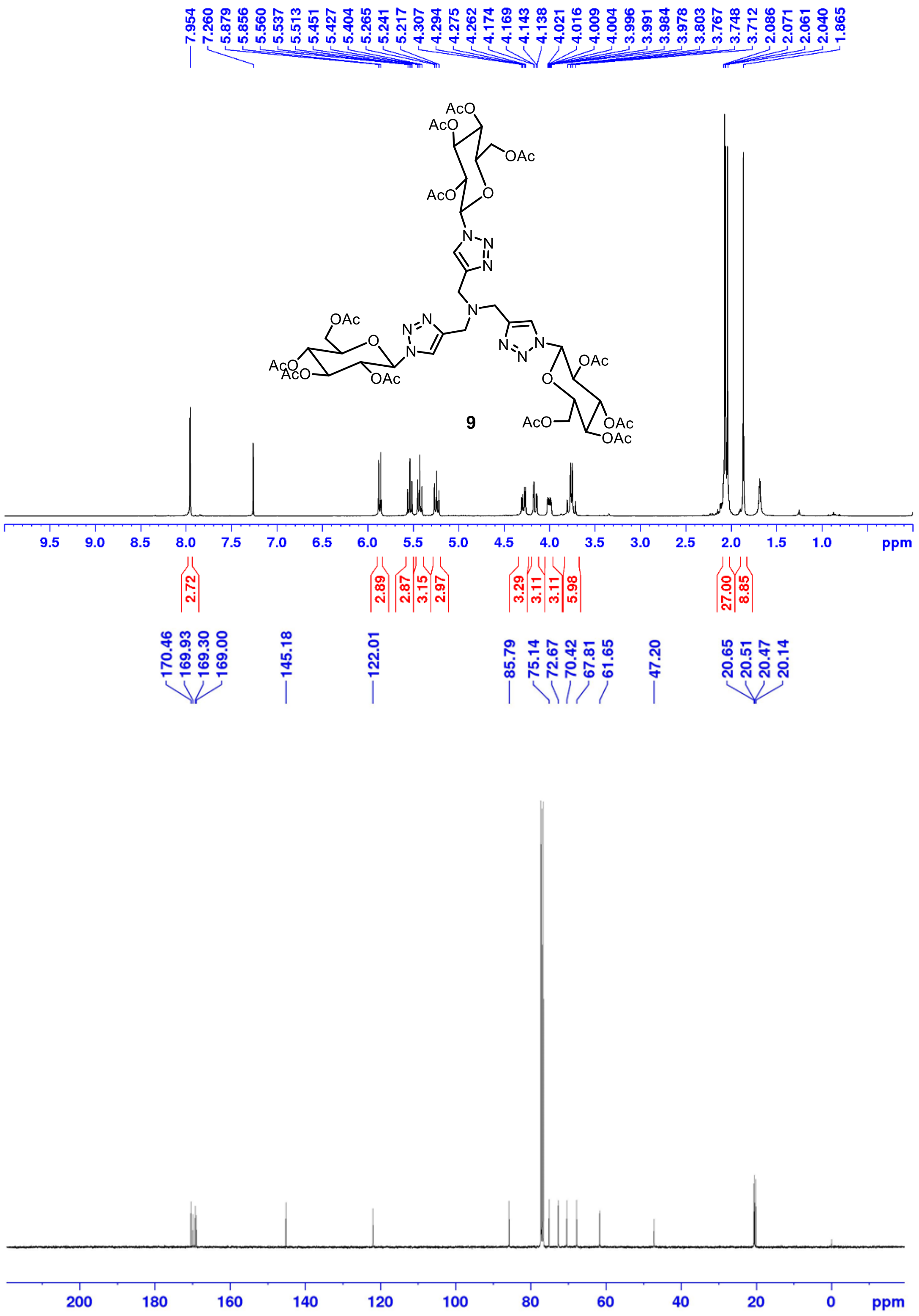

Figure S46. ^{1}H ($CDCl_3$, 400 MHz) and ^{13}C ($CDCl_3$, 100 MHz) NMR spectra of compound **9**.

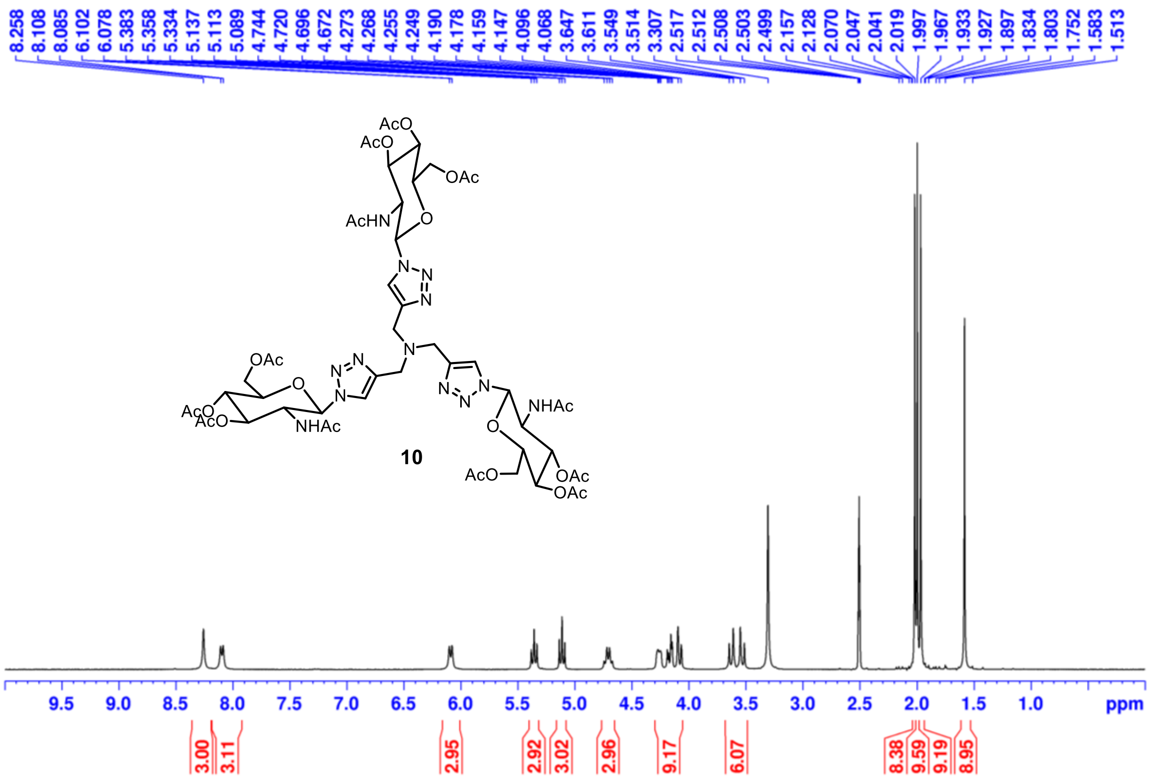

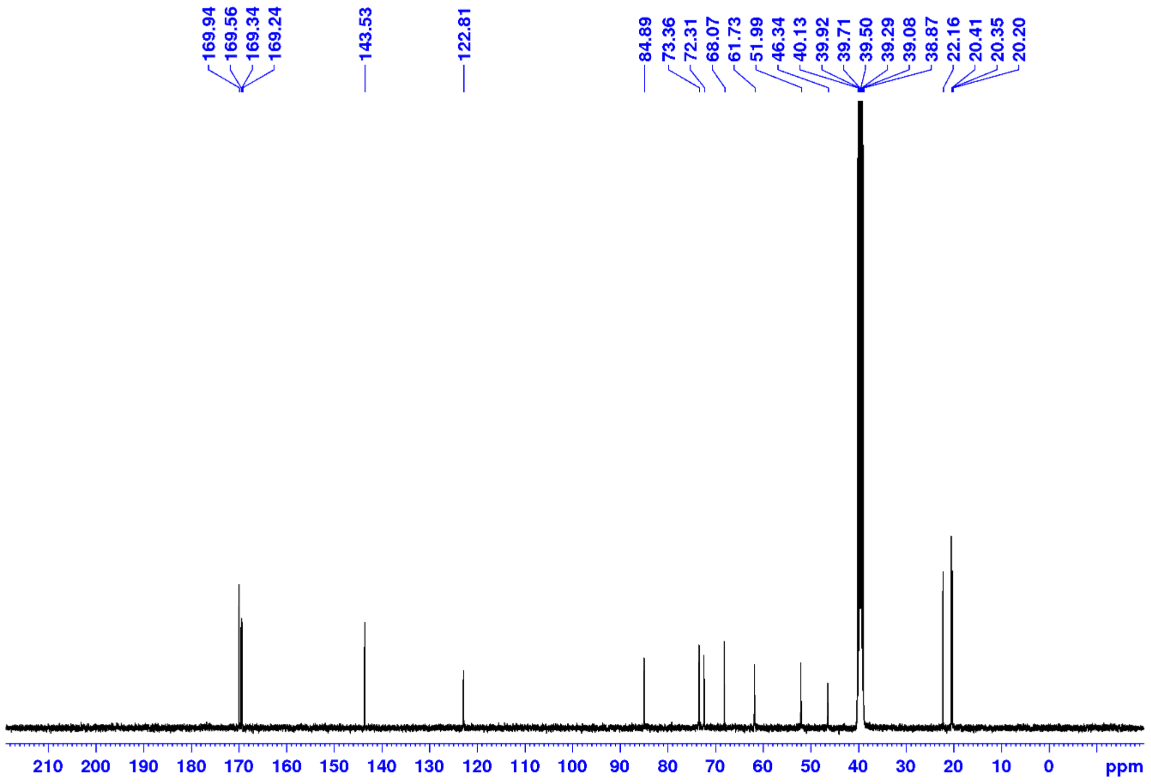

Figure S47. ^{1}H (d_6-DMSO, 400 MHz) and ^{13}C (d_6-DMSO, 100 MHz) NMR spectra of compound **10**.

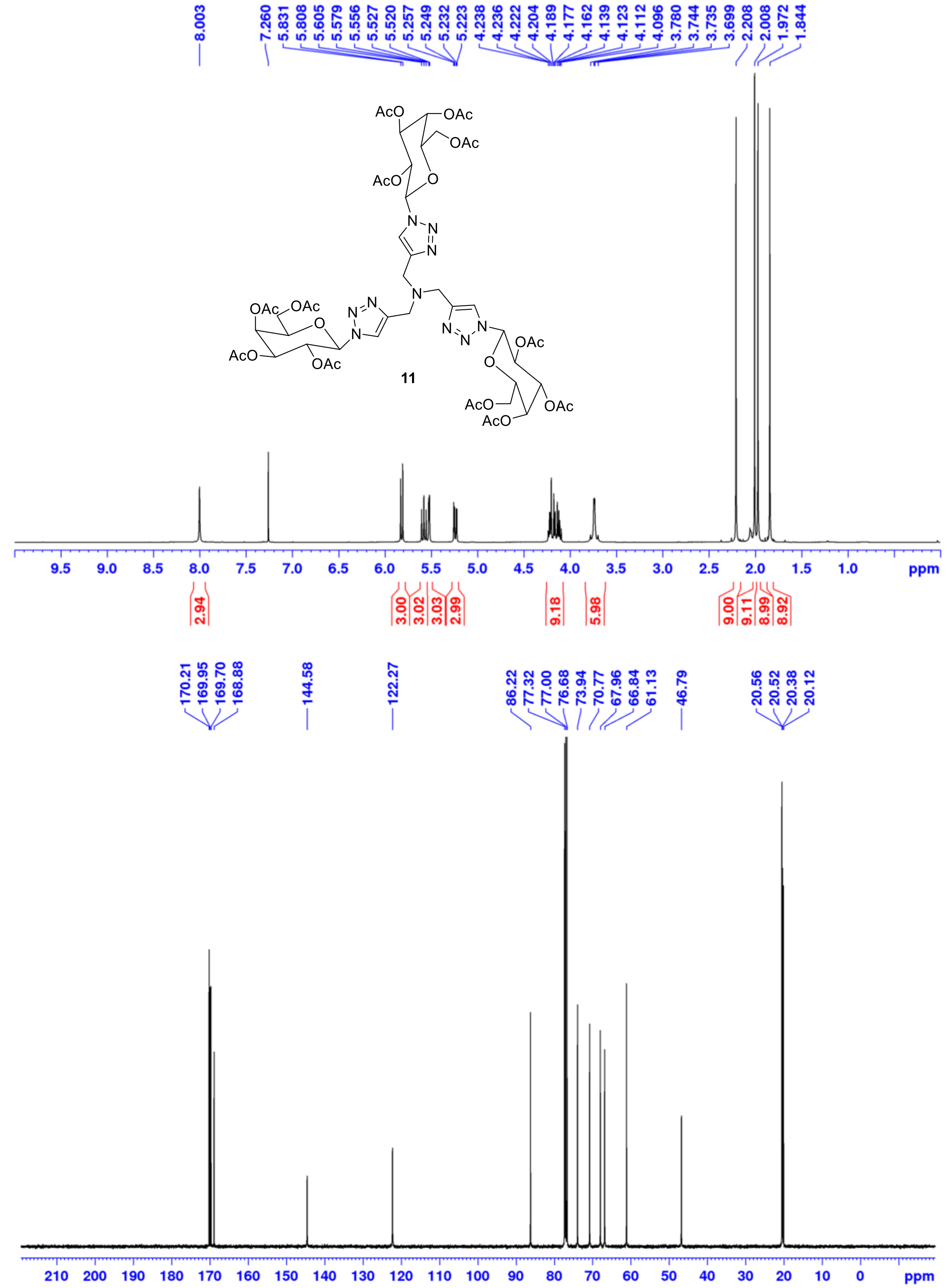

Figure S48. ^{1}H ($CDCl_3$, 400 MHz) and ^{13}C ($CDCl_3$, 100 MHz) NMR spectra of compound **11**.

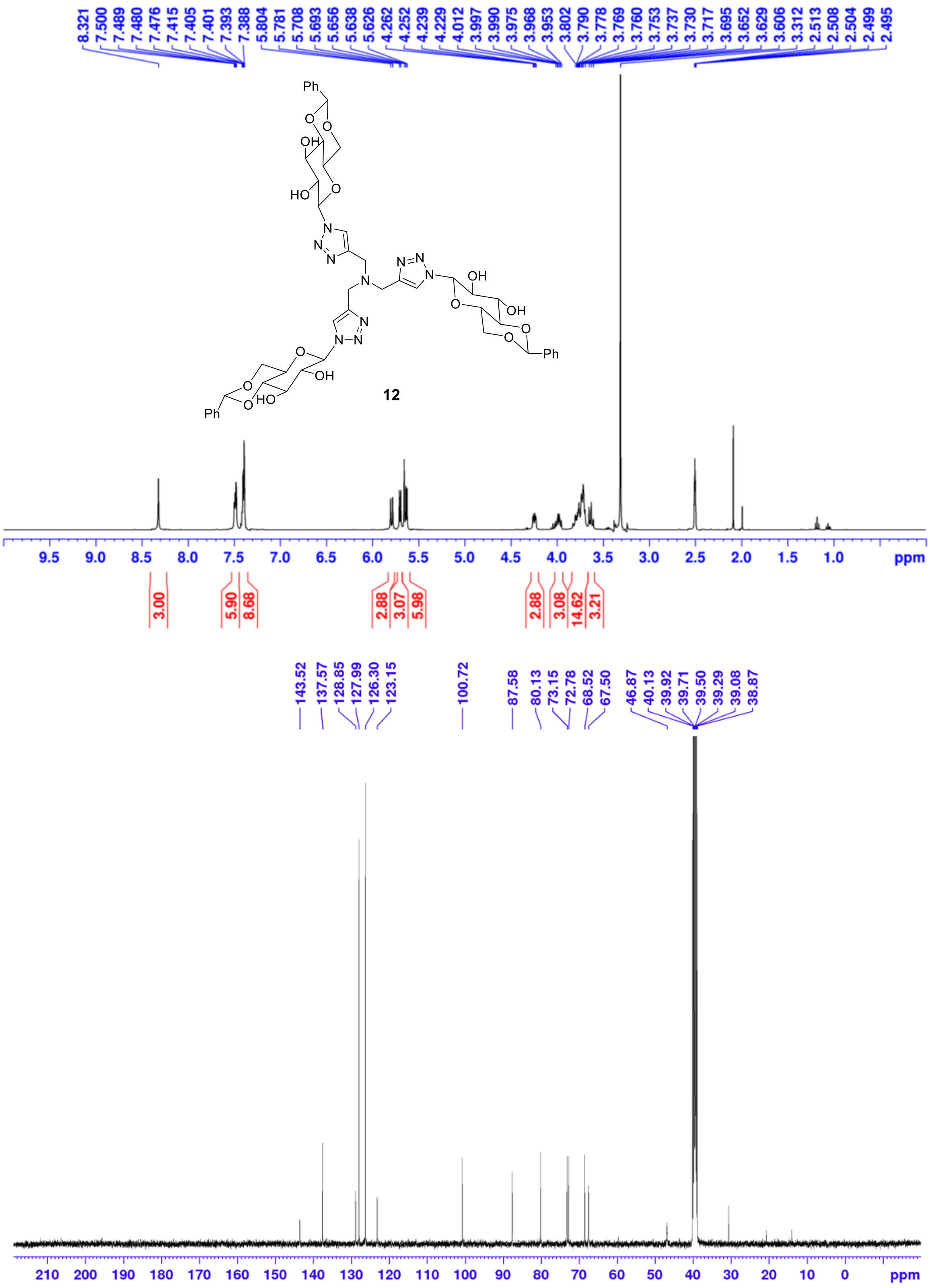

Figure S49. ^{1}H (d_6-DMSO, 400 MHz) and ^{13}C (d_6-DMSO, 100 MHz) NMR spectra of compound **12**.

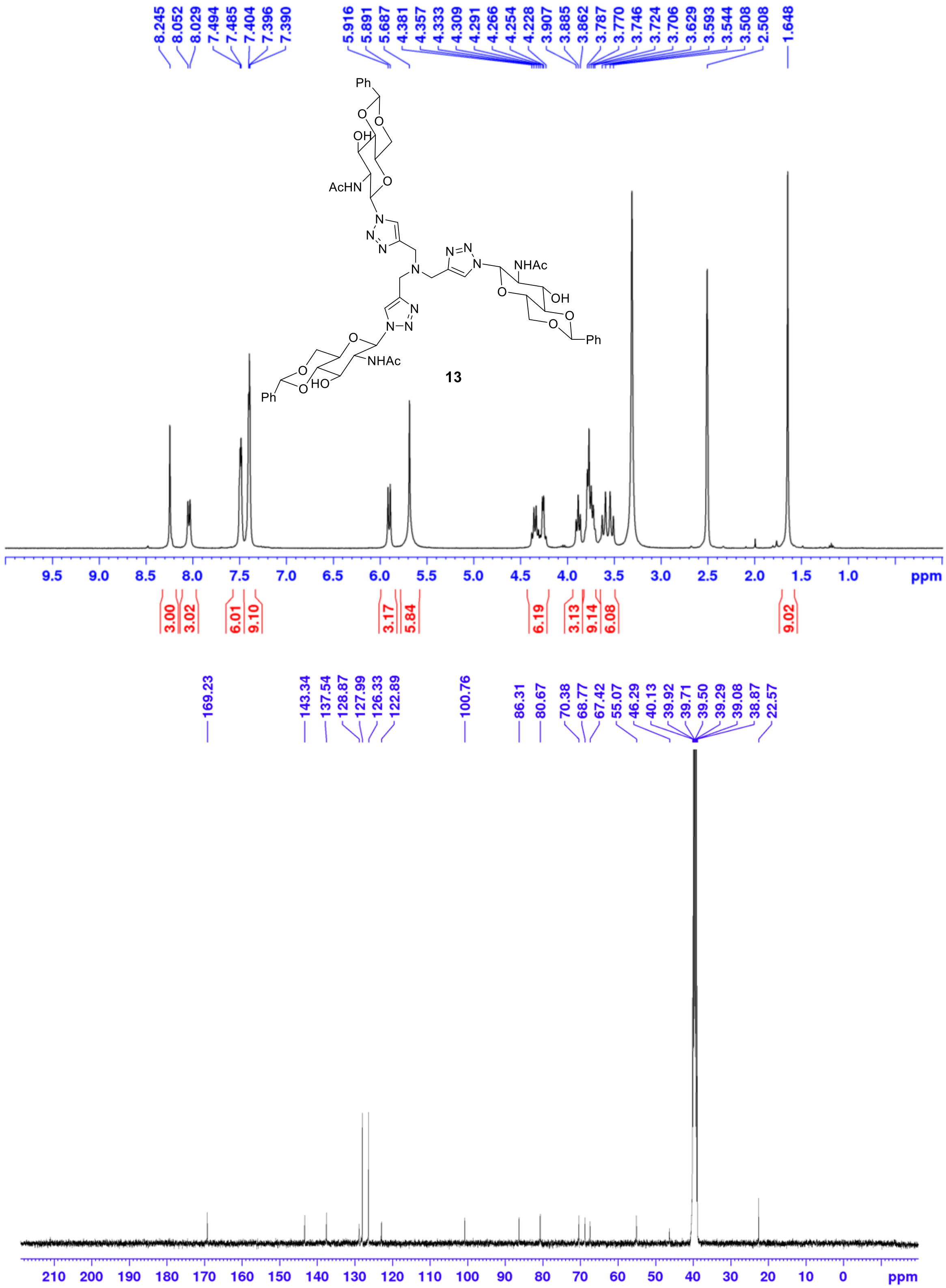

Figure S50. ^{1}H (d_6-DMSO, 400 MHz) and ^{13}C (d_6-DMSO, 100 MHz) NMR spectra of compound **13**.

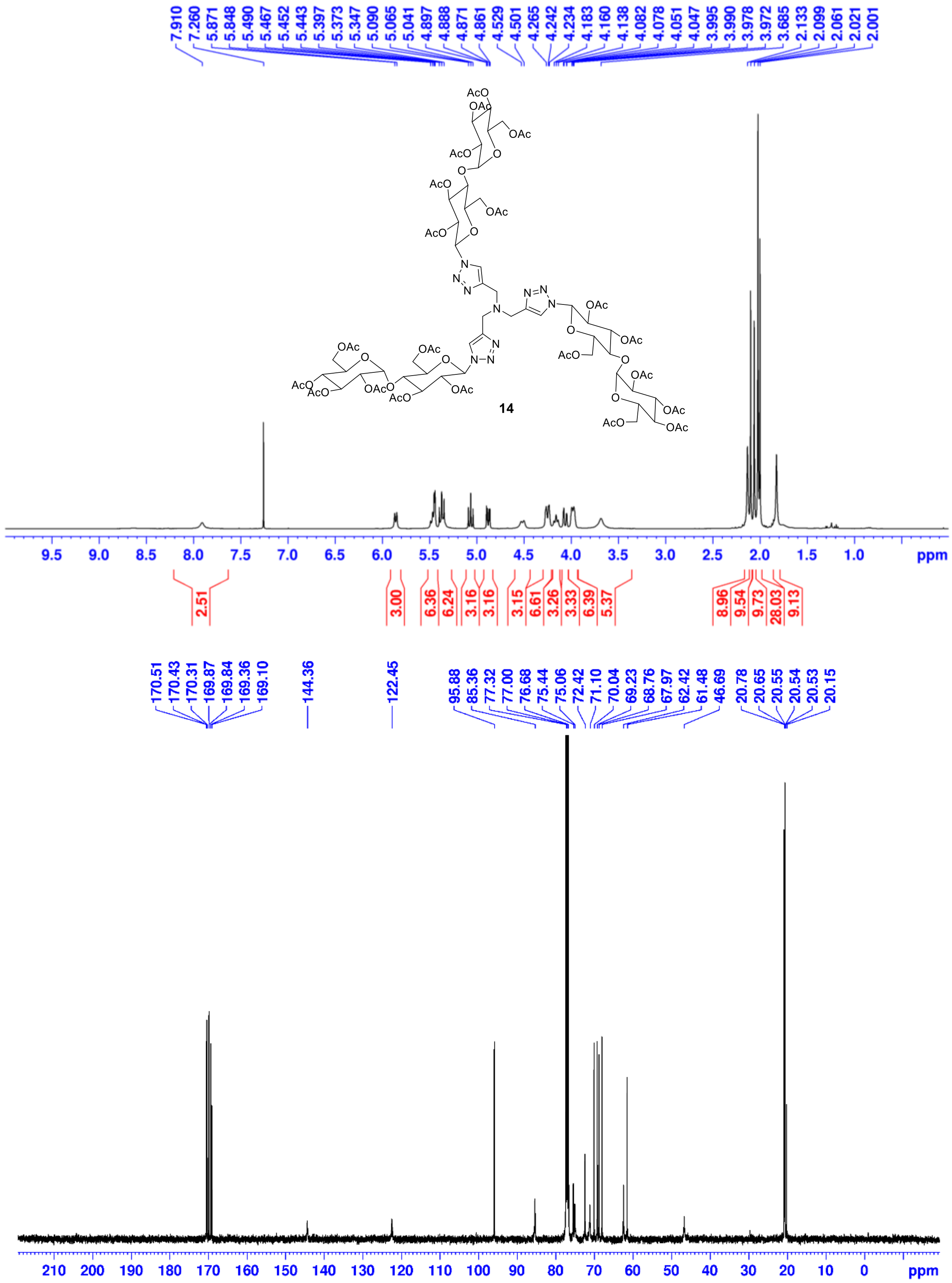

Figure S51. ^{1}H ($CDCl_3$, 400 MHz) and ^{13}C ($CDCl_3$, 100 MHz) NMR spectra of compound **14**.

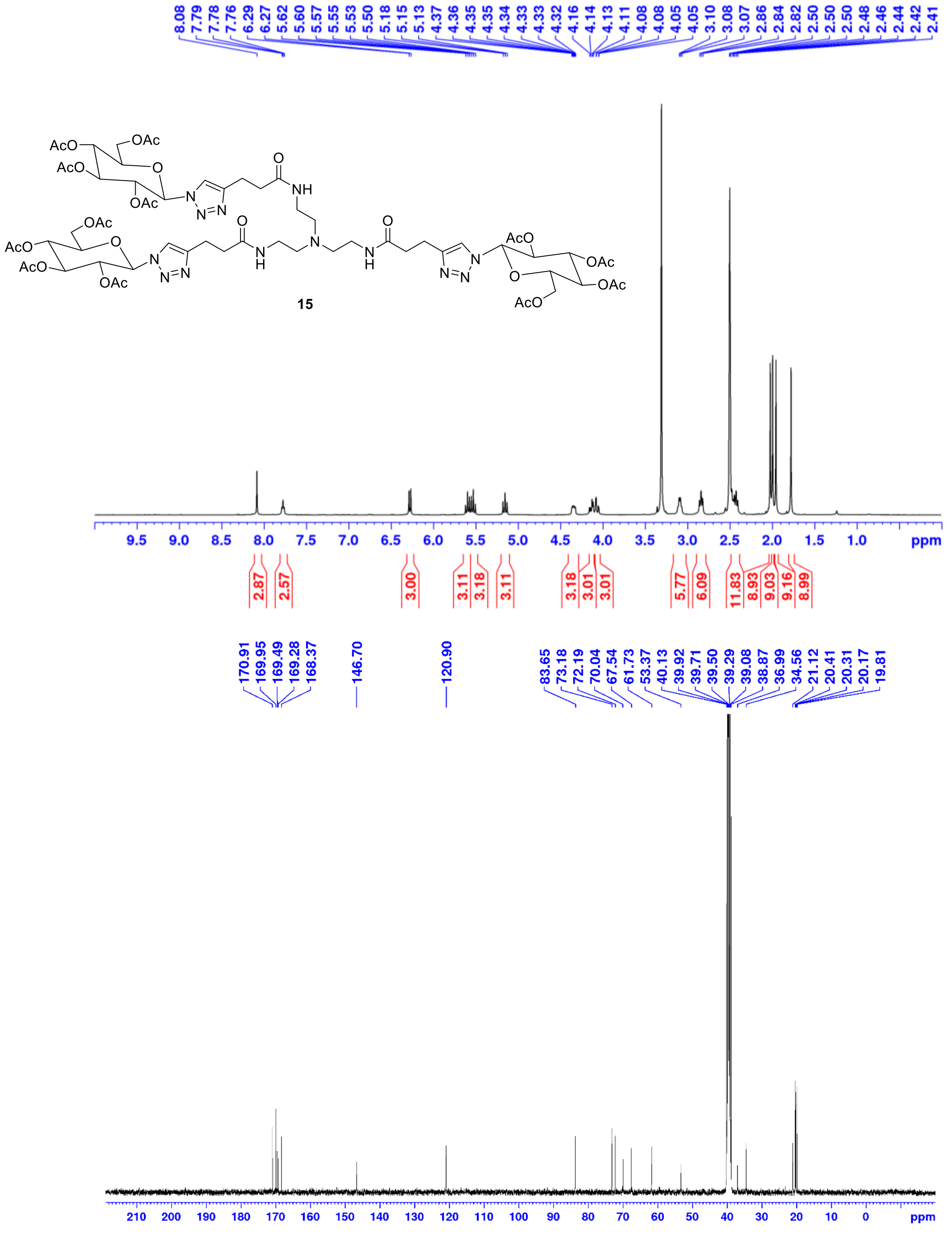

Figure S52. ^{1}H (d_6-DMSO, 400 MHz) and ^{13}C (d_6-DMSO, 100 MHz) NMR spectra of compound **15**.

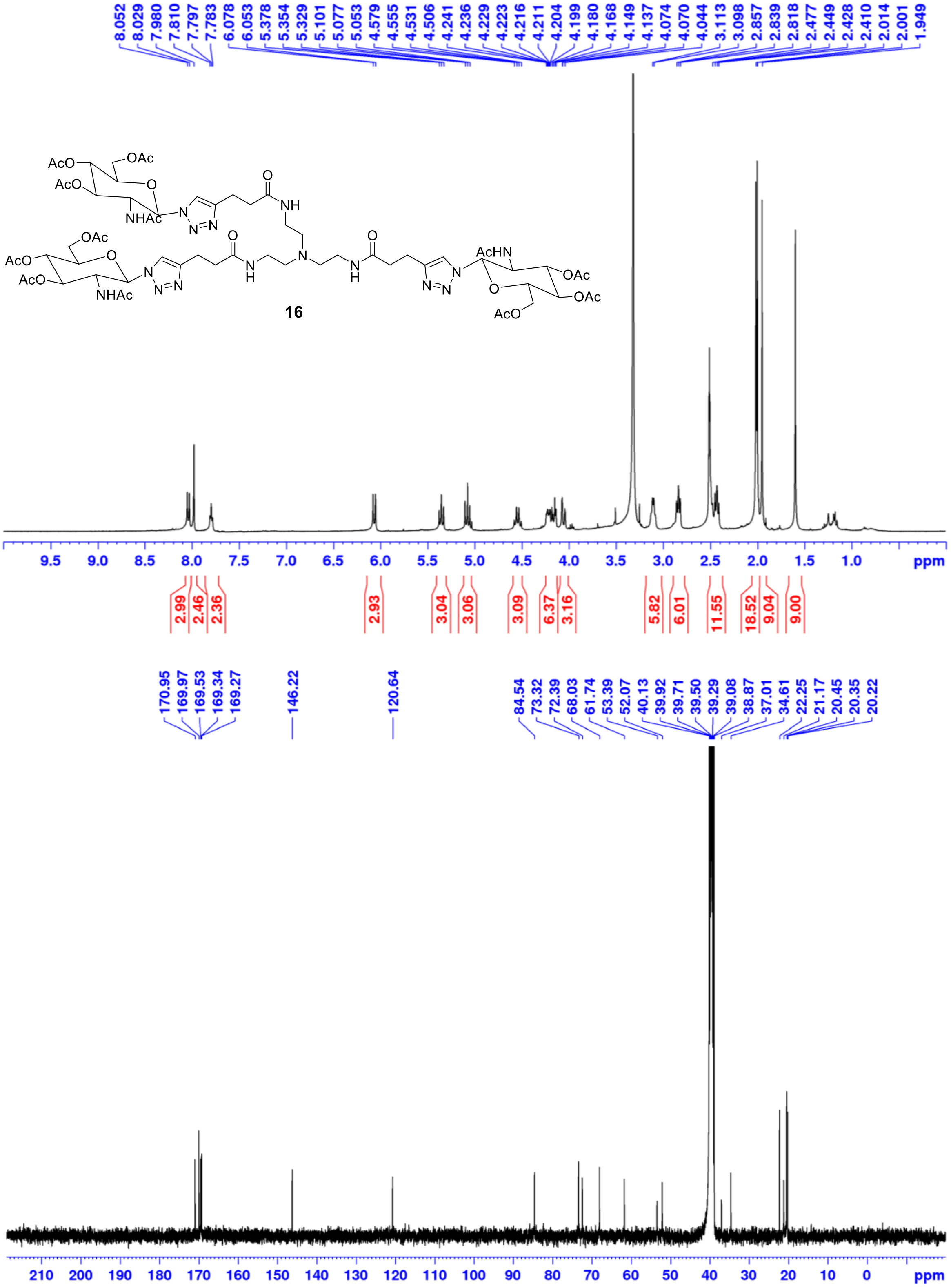

Figure S53. ^{1}H (d_6-DMSO, 400 MHz) and ^{13}C (d_6-DMSO, 100 MHz) NMR spectra of compound **16**.

Part IV. 2D NMR spectra of compounds 9-12 and 14-15

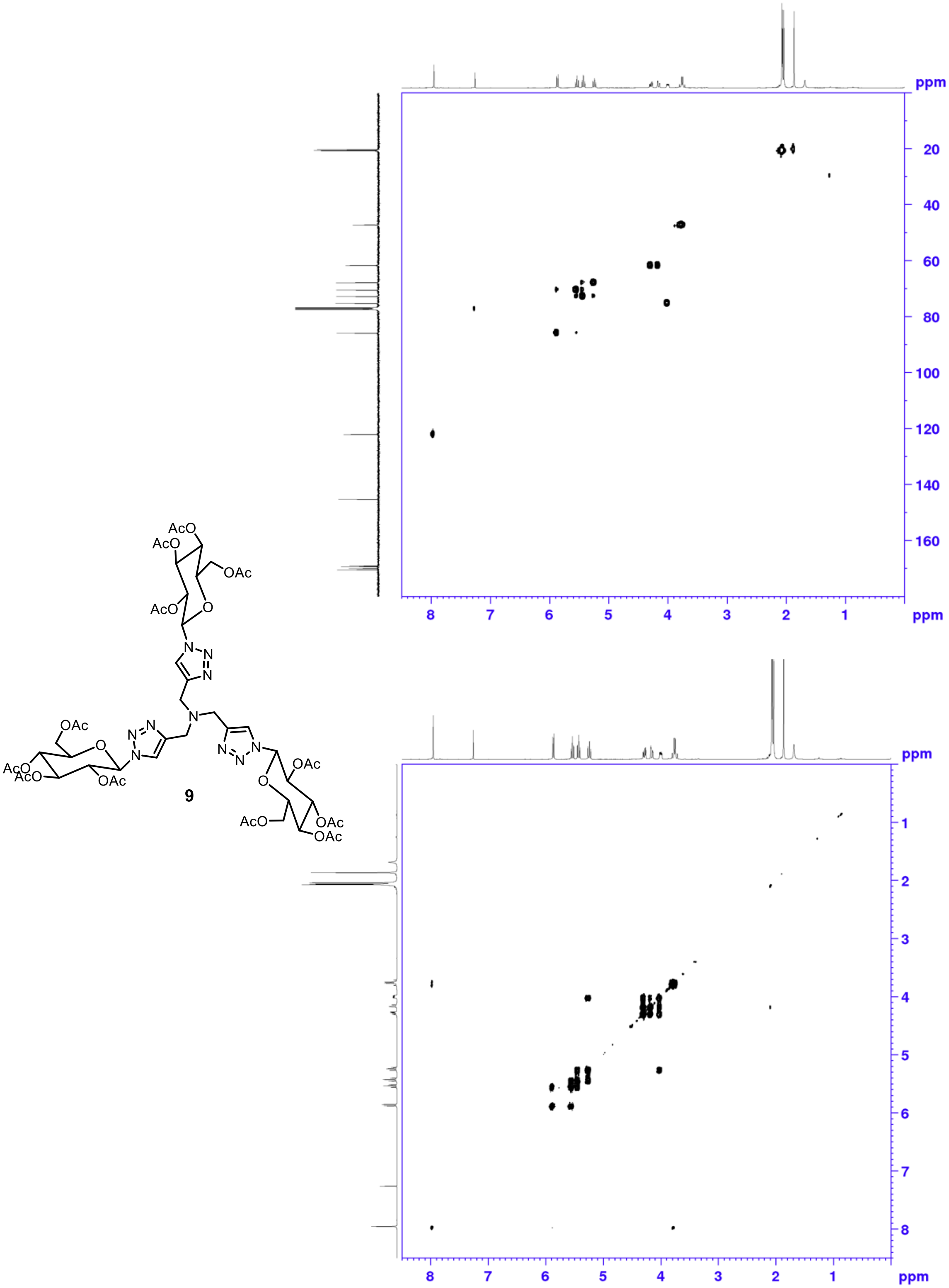

Figure S54. HSQC (top) and COSY (bottom) spectra of compound **9** in $CDCl_3$.

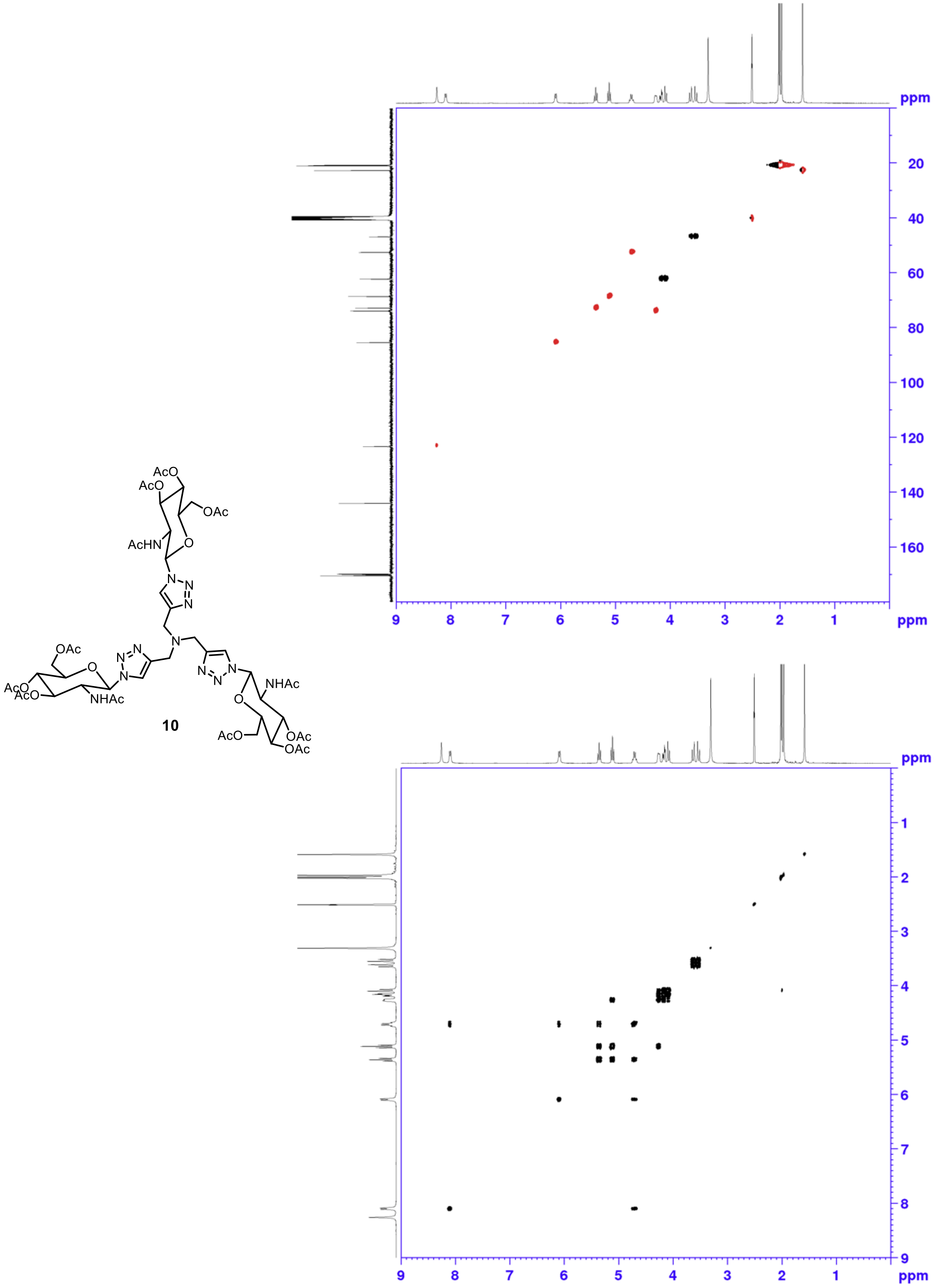

Figure S55. HSQC (top) and COSY (bottom) spectra of compound **10** in d_6-DMSO.

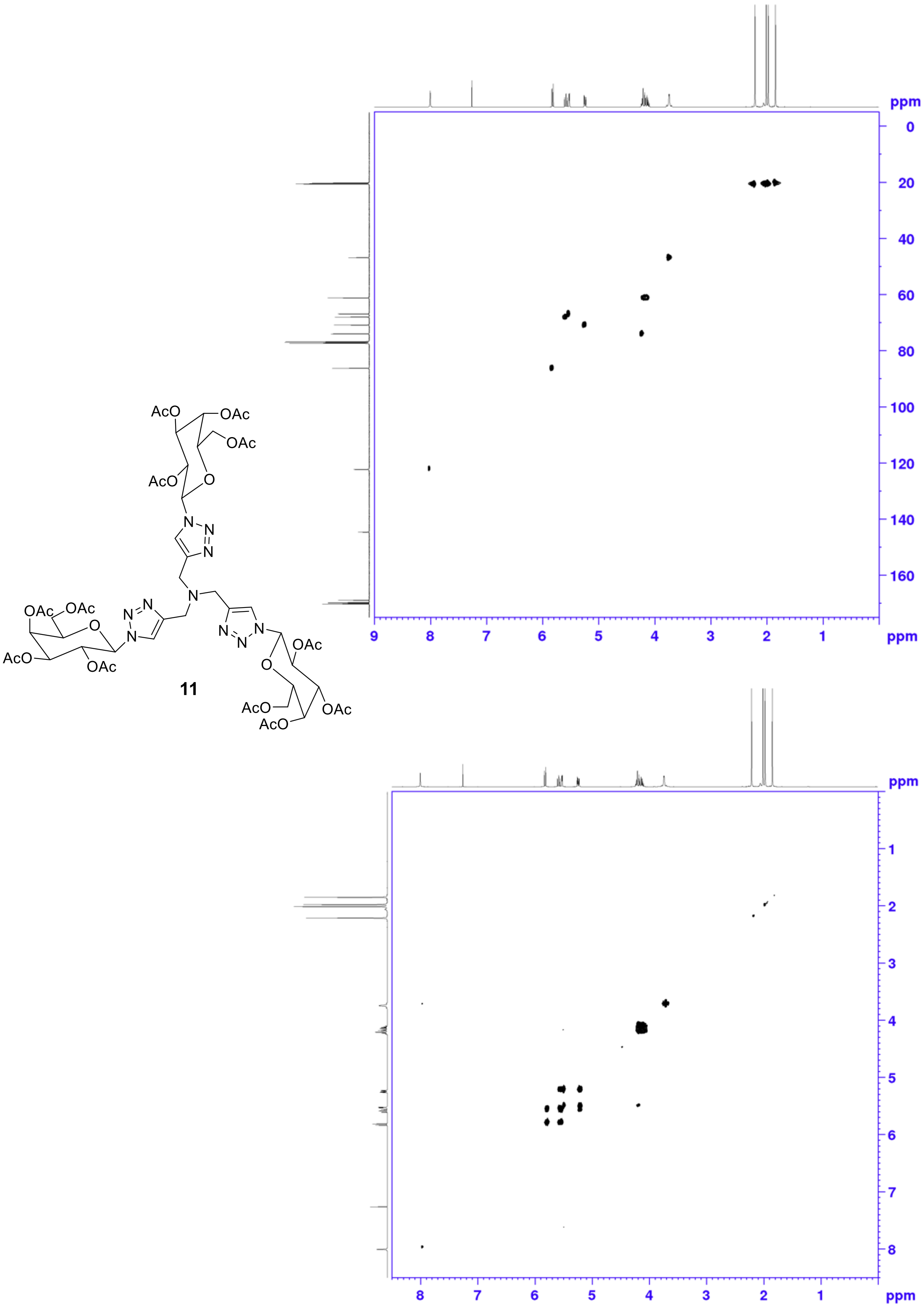

Figure S56. HSQC (top) and COSY (bottom) spectra of compound **11** in $CDCl_3$.

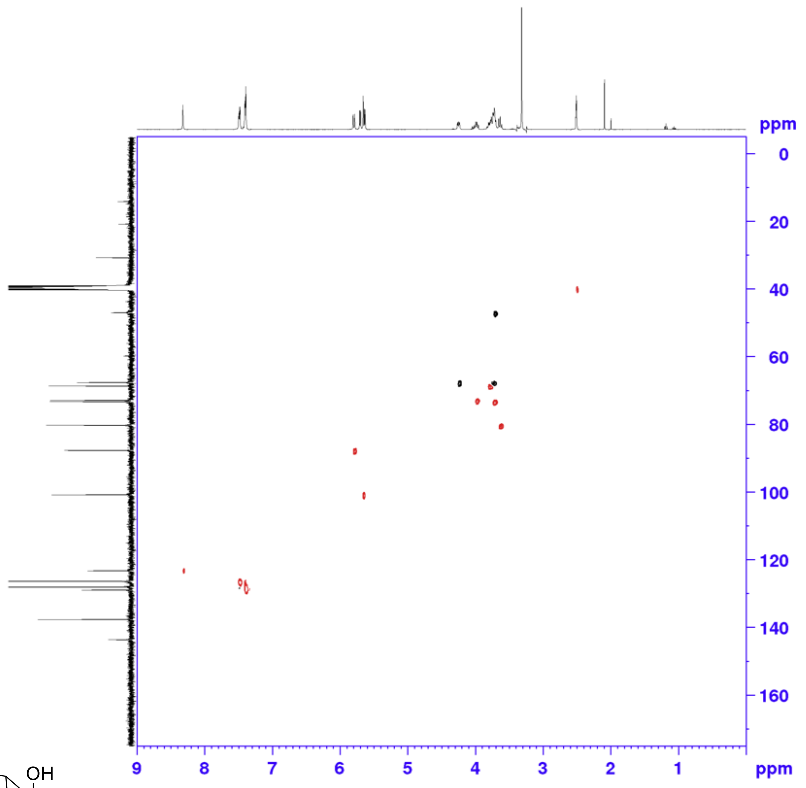

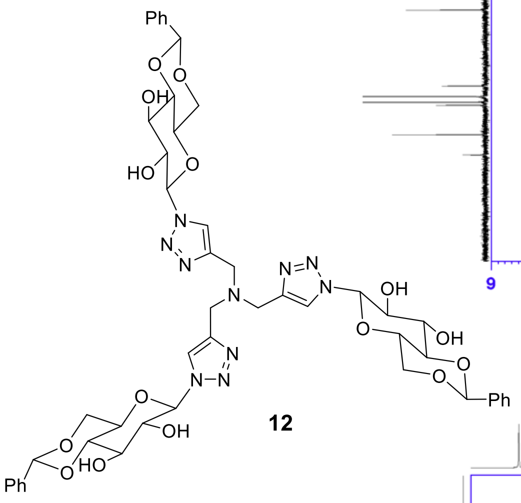

12

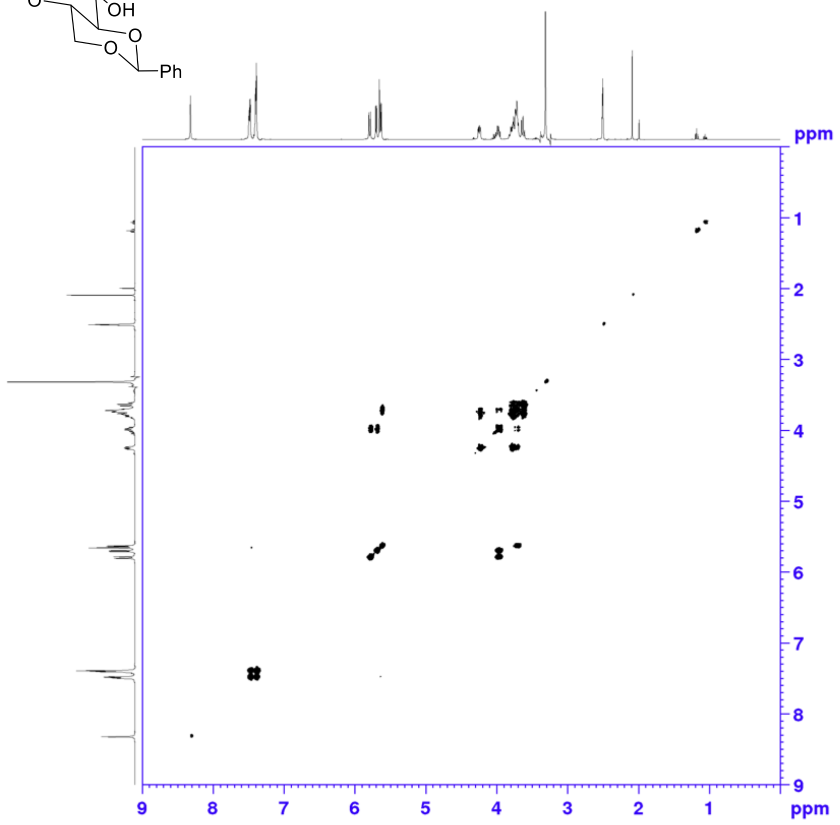

Figure S57. HSQC (top) and COSY (bottom) spectra of compound **12** in d_6-DMSO.

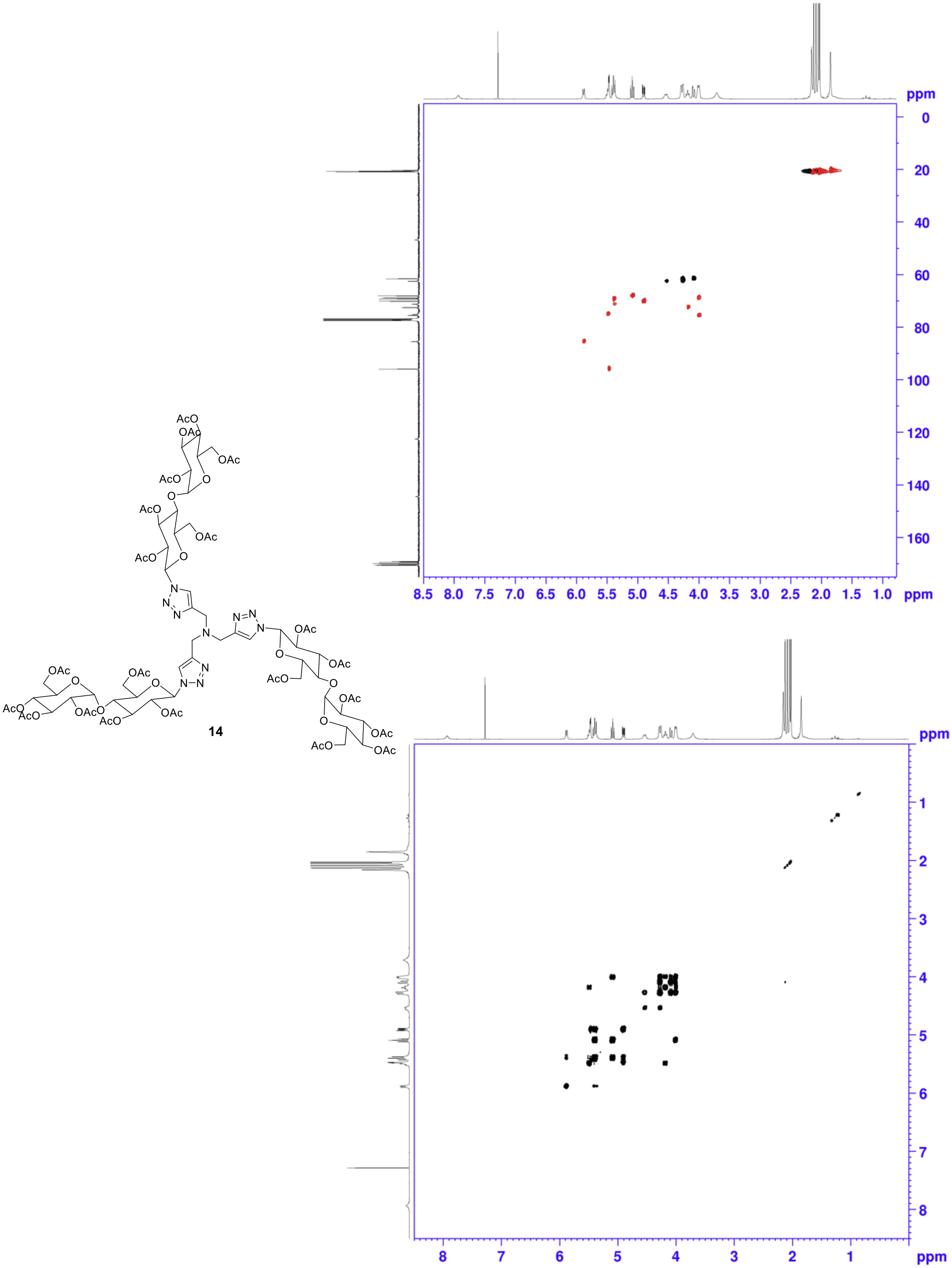

Figure S58. HSQC (top) and COSY (bottom) spectra of compound **14** in $CDCl_3$.

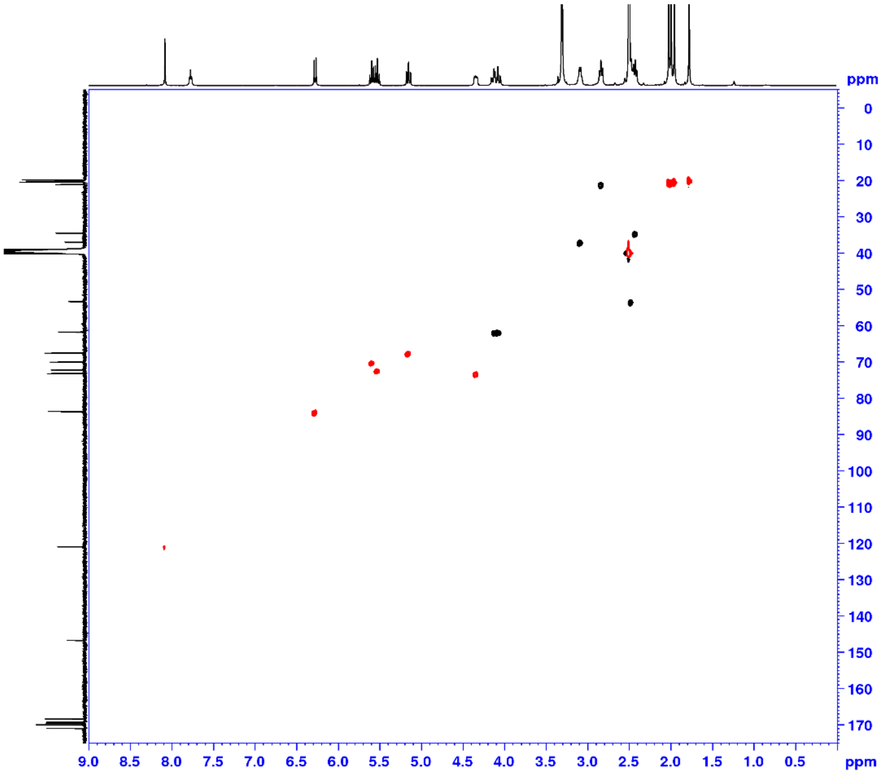

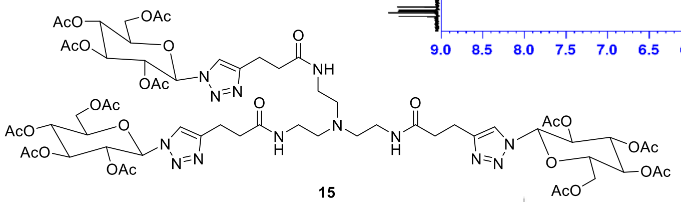

15

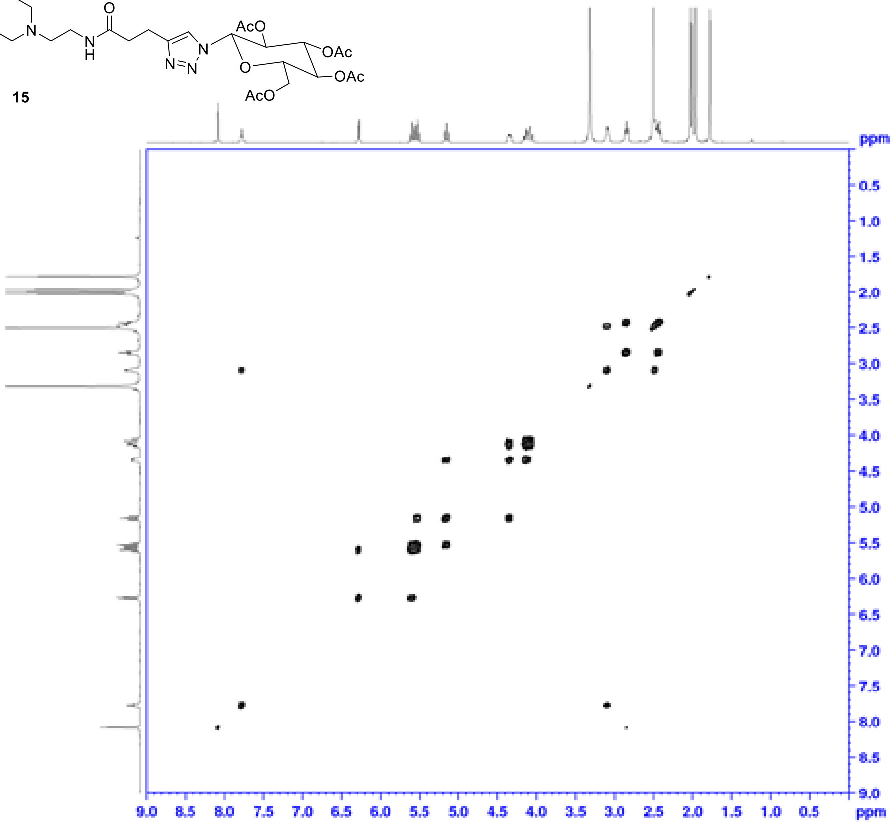

Figure S59. HSQC (top) and COSY (bottom) spectra of compound **15** in d_6-DMSO.
